# A high-speed attention network for MHC-bound peptide identification and 3D modeling

**DOI:** 10.1016/j.crmeth.2026.101364

**Published:** 2026-03-31

**Authors:** Coos A.B. Baakman, Giulia Crocioni, Cunliang Geng, Daniel T. Rademaker, David Frühbuß, Yannick J.M. Aarts, Li C. Xue

**Affiliations:** 1 Medical BioSciences Department, Radboud University Medical Center, 6525 GA Nijmegen, the Netherlands; 2 Netherlands eScience Center, 1098 XH Amsterdam, the Netherlands; 3 Biosystems Data Analysis, University of Amsterdam, 1090 GE Amsterdam, the Netherlands; 4 HIMS-Biocat, University of Amsterdam, Science Park 904, 1098 XH Amsterdam, the Netherlands; 5 Amsterdam Machine Learning Lab, University of Amsterdam, Science Park 900, 1098 XH Amsterdam, the Netherlands; 6 Adaptation Physiology Group, Department of Animal Sciences, Wageningen University and Research, PO Box 338, 6700 AH, Wageningen, the Netherlands

**Keywords:** cancer immunotherapy, peptide-MHC complexes, attention neural networks, binding prediction, 3D modeling, physics-informed AI, task-specific AI

## Abstract

We developed SwiftMHC, an ultra-fast and accurate structure-based framework for peptide-MHC (pMHC) modeling and binding affinity prediction. Using task-specific deep learning trained on physics-derived synthetic data, SwiftMHC predicts pMHC binding affinities in 0.009 s per case on a single A100 GPU when running in batch mode, offering improved speed compared with leading sequence-based tools such as netMHCpan and MHCflurry while maintaining competitive accuracy. In addition, SwiftMHC generates all-atom 3D pMHC structures with a median Cα-RMSD of 1.32 Å against crystallographic benchmarks, matching or exceeding state-of-the-art methods such as AlphaFold2-finetune but at a lower computational cost. Optimized for HLA-A∗02:01 9-mer peptides but readily extensible to other alleles, SwiftMHC unites structural insight with high-throughput scalability to accelerate safe and effective epitope discovery in cancer immunotherapy.

## Highlights

• SwiftMHC predicts 3D pMHC structures and affinities in milliseconds
• A small, physics-informed model outperforms larger models in data-scarce tasks
• Potentially enables 3D TCR cross-reactivity assessment using structural models

## Motivation

Identifying tumor-derived peptides that stably bind patient MHC molecules and elicit T cell responses is fundamental to developing effective and safe immunotherapies. However, progress in this area remains limited by two key challenges: (1) the scarcity of experimentally characterized peptide-MHC (pMHC) complexes across diverse alleles and (2) the computational cost of accurate 3D modeling. Sequence-based predictors enable large-scale screening but are limited in their ability to generalize beyond well-studied alleles or infer structural determinants of immunogenicity. Structure-based methods, by contrast, can directly capture the physicochemical features that shape TCR recognition, yet their use is restricted by slow inference times. Bridging this gap between speed and structural accuracy is essential not only for scalable peptide-MHC binding prediction but also for understanding how subtle 3D surface differences between self- and non-self-peptides influence immune recognition and therapy safety.

## Graphical abstract

**Figure:**
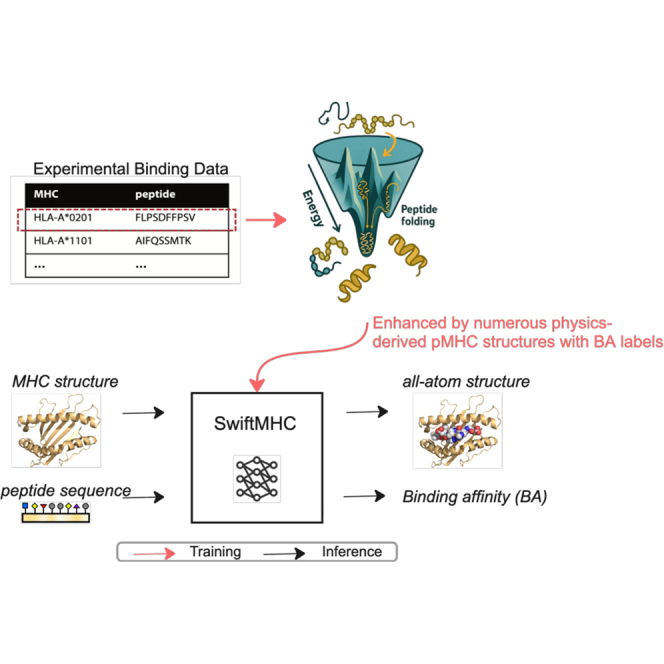

## Teaser

Baakman et al. introduce SwiftMHC, an ultra-fast AI method that predicts peptide-immune molecule interactions and their 3D structures. The framework enables rapid screening of potential cancer therapy targets, helping bridge the gap between computational speed and structural insight.

## Introduction

**Figure 1:**
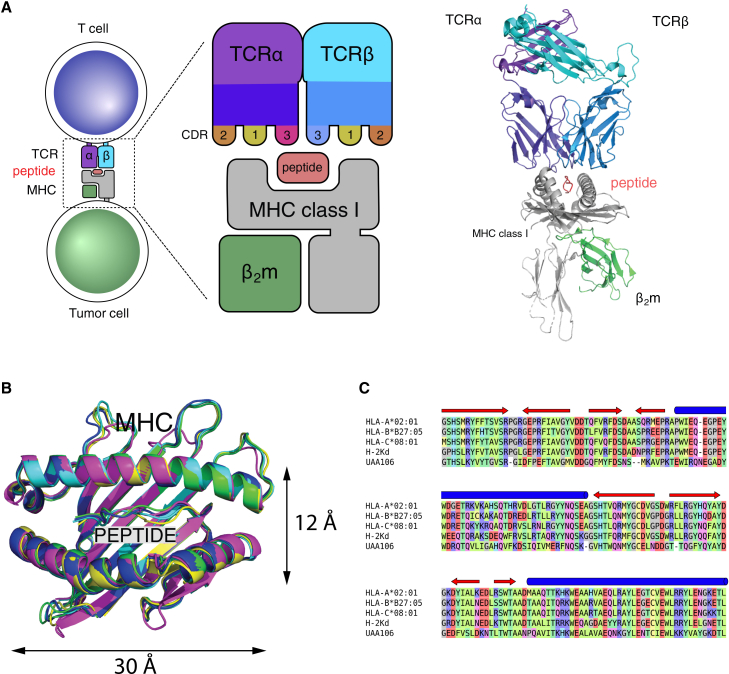
The TCR-peptide-MHC class I (TCR:pMHC-I) complex and neoantigens: their central role in immune surveillance and T cell-mediated immune attacks on tumor cells (A) TCR nomenclature and the TCR:pMHC-I complex. A TCR has two chains (*α* and *β* chains), each having three loops (CDR1, CDR2, and CDR3), where CDR3 plays the primary role in interacting with the peptide. The image on the right shows the structural representation of a TCR:pMHC-I complex based on PDB: 7RTR. (B) Structural alignment of five different MHC alleles: HLA-A∗02:01 (*Homo sapiens*, PDB: 5HHN), HLA-B∗27:05 (*Homo sapiens*, PDB: 5IB2), HLA-C∗08:01(*Homo sapiens*, PDB: 4NT6), H-2Kd (*Mus musculus*, PDB: 1VGK), and UAA106 (*Ctenopharyngodon idella*, bony fish, PDB: 6LBE). The MHC G-domains all consist of two α-helices and a β-sheet. The peptides are bound in the MHC-binding groove. The root-mean-square deviation (RMSD) values for the structural alignment of these G-domains are all below 0.8 Å, demonstrating highly conserved MHC structures across different alleles and species. (C) Sequence alignment of MHC G-domains. The sequence alignment of the MHC G-domains from the five alleles shown in (B) demonstrates high diversity in the MHC sequences. The alignment was generated using Clustal Omega^4^ and visualized in MRS,^5^ with colors representing the charge, hydrophobicity, size, and shape of amino acid side chains. Blue cylinders above the sequences indicate α-helices, while red arrows denote β-strands.

Advances in understanding T cell immunity have led to breakthroughs in cancer immunotherapy, where the immune system is trained to recognize and destroy cancer cells, offering a promising alternative to traditional treatments with fewer side effects.^1^ T cells are activated when the T cell receptor (TCR) recognizes tumor-specific peptides presented on the tumor cell surface by the class I major histocompatibility complex (MHC-I) proteins (Figure 1A for molecular details). However, these therapies face challenges, including high costs, long development time, and toxicity risks.^1^^,^^2^ Currently, the limited number of available target peptides is one of the major bottlenecks of cancer immunotherapies.^3^

Many predictive methods have been developed to identify MHC-binding peptides,^6^^,^^7^ which have recently made significant contributions to cancer immunotherapy designs.^8^ State-of-the-art (SOTA) tools, such as MHCflurry 2.0^9^ and netMHCpan 4.1,^10^ use both the sequence of the MHC-binding groove and the peptide sequence as input. However, these sequence-based approaches have notable limitations: they require large training datasets due to their reliance on sequence information and have limited generalizability on unseen alleles,^11^ they overlook critical structural features of peptide-MHC (pMHC) interactions, and they struggle to handle peptides of varying lengths. Moreover, they do not provide 3D structural models, which are essential for understanding immunogenicity and guiding TCR design.

Alternatively, 3D structure-based approaches offer several compelling advantages: (1) they naturally handle peptide length variability in 3D space, (2) they are sensitive to mutations in both the spatial and energy landscapes, (3) they are potentially more robust for rare alleles due to the high conservation of MHC structures^11^ (Figures 1B and C), and (4) 3D structures provide fundamental insights into the mechanisms of immunotherapies. However, experimental methods like X-ray crystallography, NMR, and cryo-EM are labor intensive and cannot keep up with the diversity of pMHCs. To date, only ∼1,000 pMHC structures are available in the Protein DataBank (PDB, www.rcsb.org),^12^ in contrast to the high diversity of human leukocyte antigens (HLAs, over 40,000 variants identified).^13^ Therefore, complementary 3D modeling techniques are valuable tools.

Physics-based homology modeling tools such as PANDORA^14^ and APE-Gen 2.0^15^ can generate multiple MHC-I/II models and provide energy scores indicative of binding affinity (BA). However, they remain computationally expensive (seconds to minutes per case). AlphaFold,^16^^,^^17^ a powerful deep learning (DL) framework for 3D modeling, also faces limitations: it relies on computationally heavy modules to process multiple sequence alignments (MSAs), which are often unavailable for short peptides lacking evolutionary depth. AlphaFold2-FineTune,^18^ a variant adapted for pMHC BA prediction, bypasses MSA searches by using query-to-template alignments but still depends on the costly Evoformer module—a limitation also reported by Mikhaylov et al.^19^

Other methods attempt to address these challenges. MHCfold^20^ uses convolutional neural networks to predict 3D pMHC structures and then uses multi-headed attention on the predicted structures to predict BAs. However, it requires additional tools (e.g., MODELLER^21^ or SCWRL4^22^) for side-chain reconstruction. Importantly, recent studies^11^ have shown that 3D model-based BA predictors have better generalizability than sequence-only methods on unseen alleles (6%–16%), but they typically follow a two-step pipeline: (1) generating 3D pMHC models (seconds to minutes per case) and (2) applying geometric deep learning (GDL) networks for BA estimation. This workflow is time consuming and poorly scalable, particularly for large-scale screening of patient-derived mutations. Thus, there is a clear need for a time-efficient system capable of simultaneously predicting 3D structures and BAs in a single step.

**Figure 2:**
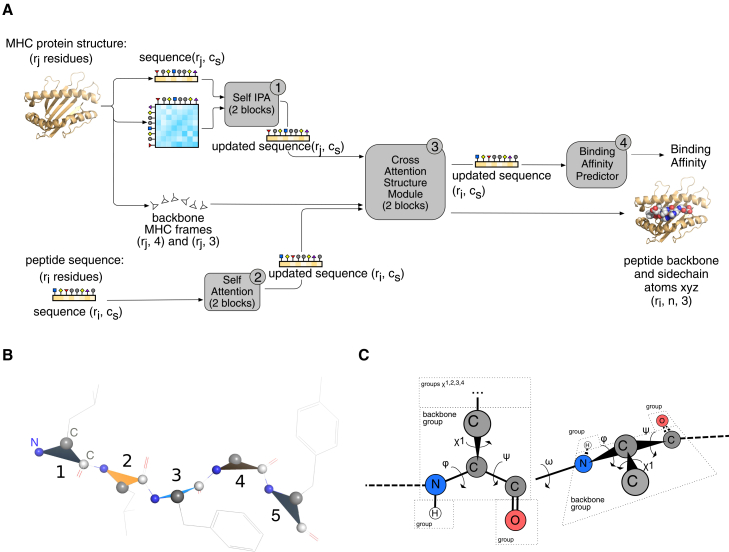
SwiftMHC architecture (A) Flow diagram of SwiftMHC. The inputs are the structure of the MHC G-domain, consisting of *r*_*j*_ residues and a peptide sequence of *r*_*i*_ residues. The model comprises four key modules: (1) the MHC Self Invariant Point Attention (IPA) module, which processes the MHC structure (for details, see Figure S5); (2) the Peptide Self Attention module, which processes the peptide sequence (Figures S6A and S6B); (3) the Cross-Attention Structure module, which captures interactions between MHC and peptide residues (Figure S7); and (4) the BA Predictor module. Residue distances in the MHC structure are encoded in a proximity matrix, calculated as 1/(1 + *d*_*ij*_), where *d*_*ij*_ is the shortest distance between heavy atoms of residue pairs. The MHC backbone is described using local frames (Figures S4A and S4B). Both MHC and peptide sequences are represented as one-hot encoded tensors with dimensionality *c*_*s*_, and the structures have maximally *n* atoms per residue. (*c*_*s*_ = 32 and *n* = 14 in this study). (B) Local frames for a peptide composed of five residues. Backbone local frames are sequentially numbered. Triangles mark the atoms involved in determining the frame orientation (N, Cα, and C). (C) The torsion angle representation of a peptide with two residues. Different peptide conformations can be derived by rotating torsion angles. Each torsion angle corresponds to a rigid group. Every residue has a backbone rigid group and several side-chain rigid groups The atoms in the rigid group are repositioned as the torsion angle is modified. The backbone torsion angles are φ, ᴪ, and ω. The ω torsion angles have an empty rigid group, but the rigid groups for φ and ᴪ both contain one atom, though hydrogens are actually only added in the end and only if OpenMM is used. The side-chain torsion angles are *χ*_1_, *χ*_2_, *χ*_3_, and *χ*_4_. Their corresponding rigid groups contain side-chain atoms.

Here, we present SwiftMHC, a transformer that simultaneously predicts pMHC-I BA and generates corresponding 3D structures in milliseconds (Figure 2A). By leveraging the conserved architecture of MHC proteins and avoiding computationally intensive MSA-based modules like AlphaFold’s Evoformer, SwiftMHC ensures rapid and accurate predictions. To address the challenge of modeling peptide conformations without evolutionary constraints, we enhance the training dataset with physics-based 3D models and biochemistry-derived BA data, enabling synergistic improvements in both structural and affinity predictions through residue-to-residue attention mechanisms.

With SwiftMHC, we demonstrate the feasibility of designing task-specific AI models that can perform on par with large general-purpose AI systems such as AlphaFold, while being substantially faster. Importantly, SwiftMHC provides both the binding prediction and 3D structural models, crucial for downstream applications such as TCR design. Focusing on HLA-A∗02:01 9-mers—given its clinical relevance, prevalence, and the availability of data—this study provides a robust proof-of-concept. This work highlights the promise of building specialized DL systems on physics-derived 3D models, especially in data-scarce domains such as CDR3 loop modeling for TCRs and antibody-antigen interactions, where evolutionary information is absent. Finally, we outline the current limitations of SwiftMHC and potential avenues for future improvement.

Key contributions of this work include the following:

1. Showcasing the power of task-specific small AI models trained on physics-derived synthetic data in a data-scarce domain, achieving remarkable speed and precision over general-purpose big AI systems such as AlphaFold.
2. Establishing SwiftMHC’s performance for HLA-A∗02:01 9-mer peptides, one of the most prevalent alleles in the human population,^23^^,^^24^^,^^25^ enabling large-scale screening of patient tumor genomes and expanding the repertoire of candidate target peptides.
3. Overcoming the speed bottleneck of structure-based MHC-peptide AI predictors. While structure-based predictors are shown to have better generalizability than the popular sequence-base approaches, their limited speed has hindered scalability.^11^^,^^26^
4. Enabling 3D model prediction for safer therapies. By predicting pMHC 3D structures in milliseconds, SwiftMHC can help identify tumor-specific peptides distinct from self-peptides, paving the way for safer and more precise cancer immunotherapies.

## Results

### SwiftMHC architecture

The SwiftMHC network utilizes a residue-to-residue attention mechanism to learn to predict BAs and structural features based on interactions between neighboring amino acids (Figure 2A). Specifically, we apply the Self Attention networks on MHC structures and peptide sequences, respectively, to update each residue’s representations with its neighborhood information. Then, we apply cross-attention networks to learn the attention weights between MHC and peptide residues, which are expected to encode the interaction information between the peptide and the MHC. This information is used to predict the 3D models and the BAs.

Modeling flexible peptides requires efficient representation of residues and their movement in space. The bond angle between nitrogen (N), alpha carbon (Cα), and carbonyl carbon (C) is relatively fixed due to the constraints imposed by the molecular geometry and electronic structure of these atoms. Following AlphaFold2’s approach,^17^ we model each residue backbone as a rigid-body triangle with the bond angle N–Cα–C of 109°, allowing free rotation and translation in space while reducing the sampling space (Figure 2B). We use unit quaternions to represent the rotations of a peptide residue as they are more efficient and compact than 3 × 3 rotation matrices (requiring only 4 parameters vs. 9) and offer better numerical stability. Thus, the movement of each residue backbone is modeled as a local frame, which encodes the rotation (i.e., a unit quaternion represented as a vector of 4 × 1) and translation (a 3 × 1 vector) of each residue relative to a global coordinate system (supplemental information, Subsection 1.2.1). Additionally, the peptide bond torsion angle (ω) is typically 180° or 0°, adding rigidity to the backbone (Figure 2C). We take advantage of this with an auxiliary loss on ω, along with other structure losses (supplemental information, Subsection 2.4.3; Figure S4CD). See STAR Methods “Computational Efficiency” for additional design choices improving computational efficiency.

The algorithm is structured into four primary modules (Figure 2A).

1. MHC Self Invariant Point Attention (Algorithm 2 in the supplemental information): the purpose of this module is to encode each MHC residue based on its amino acid type and its structural neighbors. This module iteratively applies Self Attention to the MHC structure, drawing inspiration from the AlphaFold2 invariant point attention (IPA) technique to make the residues update each other. It was designed to represent and process interactions between amino acids. For attention weight calculation of each pair of the *r*_*j*_ MHC residues, it utilizes the one-hot-encoded sequence representation of the pairing MHC amino acid types and an *r*_*j*_ *× r*_*j*_ *× 1* proximity matrix that captures their geometric distances. These attention weights are used for updating the MHC residue features; these *r*_*j*_ updated feature vectors are the output of this Self Attention submodule.
2. Peptide Self Attention (Algorithm 3 in the supplemental information): the purpose of this module is to encode each peptide residue based on its amino acid type and its sequence neighbors. It applies Self Attention iteratively (twice, can be configured) to the peptide sequence, allowing the *r*_*i*_ residues to be notified of each other by means of residue-to-residue updating. Relative positional encoding was used to provide residue positions to the attention network. Updating allows residues to know their position in the peptide, allowing the network to distinguish between them. This peptide Self Attention submodule outputs a sequence of *r*_*i*_ updated peptide residue feature vectors.
3. Cross-Attention Structure (Algorithm 4 in the supplemental information): the purpose of this module is to update each peptide residue based on the interaction between it and the MHC residues. The updated peptide encoding is used to predict peptide structure and used as input of the BA prediction module. This module iteratively performs cross-attention between the *r*_*i*_ peptide residues and *r*_*j*_ MHC residues, employing a ross IPA on the previously updated residue feature vectors and their geometric distances. Throughout these iterations, the module not only updates the peptide features but also refines the peptide’s structural representation from an initial starting point at the center of the MHC groove. The MHC features are kept fixed in this module. The first step in this structural refinement process is the prediction of backbone frames (residue positions + orientations), followed by side-chain torsion angle prediction and finally placement of atoms in the cartesian space.
4. BA Prediction (Algorithm 8 in the supplemental information): to allow SwiftMHC to accommodate different peptide lengths, we designed a residue-wise BA predictor. The contribution of each residue to BA was predicted by a multi-layer perceptron (MLP), which processes updated peptide residue features produced by the structural module. The end result of the BA predictor is the sum of the MLP outputs over all peptide residues. This BA value is trained to approach 1 − log50000(Kd) or 1 − log50000(IC50), where K_d_ and IC_50_ are experimentally determined binding constants.

The SwiftMHC network is trained by a loss function with multiple terms on BA, frame-aligned point error (FAPE), torsion angle, and other structural violations (supplemental information, Subsection 2.6). The BA loss quantifies the difference between the predicted and true BAs between the peptide and the MHC. The FAPE measures the discrepancy in the positions of peptide atoms (both backbone and side chain) between the true and predicted structures. The torsion angle loss assesses differences in torsion angles between the true and predicted structures. Structural violations, which include abnormal bond lengths, bond angles, or atomic clashes, are incorporated only during the fine-tuning phase of training (see STAR Methods, Training).

By default, SwiftMHC predicts BAs and 3D models without running OpenMM refinement. For BA predictions, users may skip writing predicted 3D models to disk to avoid performance slowdowns, referred to as SwiftMHC-BA. Alternatively, enabling short OpenMM refinement produces high-quality models, referred to as SwiftMHC-OpenMM.

### SwiftMHC archives SOTA-level BA prediction at sequence-based speeds

In this study, we focused on HLA-A∗02:01, one of the most prevalent MHC alleles, observed in 95.7% of Caucasians and 94.3% of Native Americans^23^ and extensively studied in clinical trials.^3^ Additionally, we focused on 9-mer peptides, the most frequent binding peptide length for MHC-I

We assessed the BA prediction performance of SwiftMHC by using 7,726 BA data points obtained from the Immune Epitope Database (IEDB).^27^ To objectively evaluate SwiftMHC’s performance on unseen peptides, we clustered the data into ten groups and conducted leave-one-cluster-out cross-validation (see STAR Methods). Additionally, we compared SwiftMHC with four SOTA prediction methods: NetMHCpan 4.1,^10^ MHCflurry 2.0,^9^ AlphaFold2-FineTune,^18^ and MHCfold.^20^

**Figure 3:**
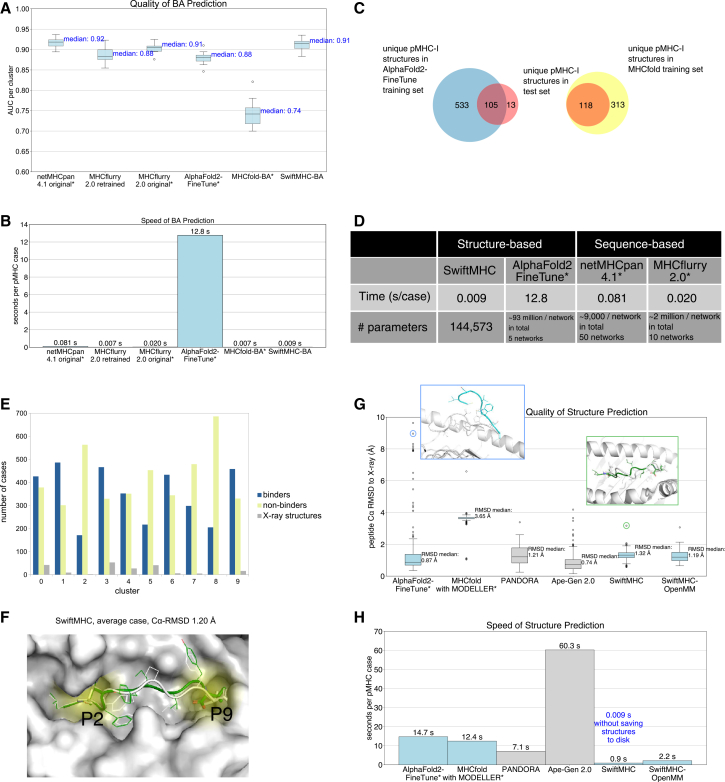
Comparison of BA prediction quality, speed, and structure prediction quality of SwiftMHC and SOTA methods (the asterisks (∗) denote the methods that were not retrained and have seen the test data, potentially leading to inflated performance estimates). (A) BA prediction quality. The boxplots display the distribution of the AUC for each of the 10 HLA-A∗02:01 9-mer peptide clusters. A perfect model has an AUC of 1, while a model with no discrimination has an AUC of 0.5. Boxplots show the median (center line), interquatile range (IQR, box; 25th–75th percentiles), and the whiskers extending to 1.5 IQR; points beyond whiskers represent outliers. (B) BA prediction speed. (C) The overlap between the unique pMHC-I structures in the training set of AlphaFold2-FineTune and MHCfold and the X-ray structures used as the test set. (D) Speed and estimated number of trainable parameters for two structure-based and two SOTA sequence-based methods. Note that though the number of parameters in netMHCpan 4.1 is small, no GPU usage was observed. (E) Data distribution of binders, non-binders, and X-ray structures across clusters. Binders, K_d_ or IC_50_ < 500 nM; non-binders, K_d_ or IC_50_ > 500 nM. (F) Example SwiftMHC-generated structure (PDB ID: 1HHK; Cα-RMSD: 1.20 Å), overlaid with its X-ray structure. P2 and P9 pockets are labeled yellow. (G) 3D modeling accuracy. Peptide Cα-RMSDs are reported against X-ray structures. Light blue: DL-based methods. Gray: Physics-based methods. SwiftMHC (with and without OpenMM refinement) performs comparably to SOTA methods. The light blue circle points to an outlier by AlphaFold2-FineTune (PDB ID: 3MRG and RMSD; 8.96 Å). The greed circle points to an outlier by SwiftMHC (PDB ID: 2GTW) where different anchors than in the X-ray structure are predicted, achieving the highest tested Cα-RMSD for SwiftMHC (3.04 Å). (H) Structure prediction speed. The bar plot displays the average modeling speed per case, measured in batch mode for each of the six structure prediction methods. SwiftMHC achieves 0.009 s/case without outputting structures in batch mode (batch size = 64). With file writing and OpenMM, SwiftMHC achieves 0.9–2.2 s/case.

To ensure objective evaluation, MHCflurry 2.0 was retrained on the same datasets as SwiftMHC. We noted that AlphaFold2-FineTune and MHCfold were not retrained and were evaluated using publicly available pretrained models due to computational restraints. As a result, at least some, if not all, of our test cases were included in the training data of these two methods (see STAR Methods and Figure 3C for details about the data overlap), which could lead to inflated performance results.

Our results demonstrate that SwiftMHC reliably predicts MHC-binding peptides, achieving a median AUC of 0.91, outperforming three SOTA methods: AlphaFold2-FineTune, MHCfold, and retrained MHCflurry 2.0 (Figure 3A). Beyond AUC, SwiftMHC also achieves superior results in terms of area under the precision-recall curve (AUPR) and Pearson’s correlation (Figures S2A and S2B). For numerical predictions, see Figure S2C.

Compared to NetMHCpan 4.1, SwiftMHC achieved nearly comparable accuracy: NetMHCpan 4.1 attained a median AUC of 0.92, a Pearson’s correlation of 0.81, and an AUPR of 0.89, whereas SwiftMHC reached 0.91, 0.80, and 0.88, respectively. Notably, NetMHCpan 4.1 and MHCflurry 2.0’s reported performances might have been slightly inflated, as some of its training data overlapped with our test set.

Where SwiftMHC clearly exceled is inference speed and structural modeling. It predicted binding in only 0.009 s per case on one A100 GPU card (when predicted structures are not written to disk), even faster than NetMHCpan 4.1 (0.081 s/case; Figure 3B) and MHCflurry 2.0 (0.020 s/case; Figure 3B). While MHCfold-BA was marginally faster (0.007s) when sidechain modeling was omitted, it exhibited a lower AUC (Figure 3A) and less accurate 3D models (Figure 3G). In contrast, SwiftMHC not only predicted BAs but also generated high-quality 3D pMHC models (see below), a critical feature for downstream TCR design.

SwiftMHC accurately predicted both high- and low-affinity binders. We evaluated SwiftMHC’s performance across different BA ranges by analyzing a confusion matrix (Figure S2E). The results show that SwiftMHC predicts most accurately for peptides with high (50–500 nM) and low (5,000–50,000 nM) binding affinities. This indicates that the model does not systematically overpredict binding and is capable of distinguishing non-binders effectively.

### SwiftMHC is robust to peptide single-point mutations

To assess SwiftMHC’s robustness to single-point mutations, we predicted BAs for 2,838 single amino acid variants derived from the original dataset of 7,726 peptides, using the network models trained on the wild-type cluster’s respective folds, and the MHC structure from PDB: 3MRD. For each mutant, we calculated the true change in ΔG (ΔΔG) relative to its wild-type counterpart and compared it to the predicted ΔΔG (Figure S2F). The overall Pearson’s correlation between predicted and true ΔΔG values was relatively low (r = 0.33). Focusing specifically on mutations that do not shift a peptide from the non-binder to binder status (IC_50_/K_d_ < 500 nM), or vice versa, SwiftMHC incorrectly classified only 11% of either the wild type or the mutant. This indicates that while fine-grained ΔΔG prediction remains challenging, SwiftMHC reliably captures whether a mutation is functionally impactful in terms of binding.

### SwiftMHC delivers angstrom-level accuracy in all-atom pMHC structure prediction with high computational efficiency

We evaluated SwiftMHC and four SOTA methods for modeling 3D pMHC-I structures: AlphaFold2-FineTune^18^ (DL-based), MHCfold^20^ (DL-based), PANDORA 2.0 (physics-based),^14^ and Ape-Gen 2.0 (physics-based)^15^ (Table 1).

**Table 1:** Methods evaluated for BA prediction and structure prediction

Methods	Type	Input	3D modeling	Numerical BA prediction	Binary BA classification
SwiftMHC	DL-based	MHC structure, peptide sequence	✔	✔	✔
AlphaFold2 -FineTune^a^	DL-based	pMHC sequence, other pMHC template structures	✔	✔	
MHCfold^a^	DL-based	pMHC sequence	✔	✔	
PANDORA	physics-based	peptide sequence, MHC allele name/sequence	✔		
Ape-Gen 2.0	physics-based	peptide sequence, MHC allele name	✔		
MHCflurry 2.0	DL-based	pMHC sequence		✔	
netMHCpan 4.1	DL-based	peptide sequence, MHC allele name		✔	

a These methods were used as provided without retraining on our own defined training sets, thereby risking train-test contamination and potential inflated performances of those methods.

To objectively evaluate the performance, we clustered 202 HLA-A∗02:01 9-mer X-ray structures together with the PANDORA models for the 7,726 IEDB entries based on the peptide sequence similarity (Figure 3E). We ensured that the X-ray structures and PANDORA 3D models from the same cluster were not included in the training datasets. PANDORA and Ape-Gen 2.0 rely on homology modeling, using X-ray structures as templates to predict new pMHC-I 3D conformations. To ensure unbiased evaluation, we excluded any X-ray structures containing the same peptide to prevent their use as templates by these methods.

SwiftMHC-OpenMM produced highly accurate pMHC-I 3D models, with a median Cα-RMSD of 1.19 Å (Figure 3G), comparable to three other SOTA methods (median Cα-RMSD: 0.74–1.21 Å), significantly outperforming MHCfold (3.65 Å). Although AlphaFold-FineTune has a lower median Cα-RMSD of 0.87 Å, this advantage likely reflects a substantial overlap between its training and our test data. Importantly, SwiftMHC produced far fewer outliers than AlphaFold-FineTune (Figure 3G), highlighting the benefit of augmenting training with task-specific, physics-derived 3D models.

In terms of computational efficiency, SwiftMHC processed each case in 0.9 s (0.009 s per case when excluding PDB file writing) and 2.2 s with OpenMM energy minimization (Figure 3H). When disk writing was bypassed, SwiftMHC performed up to thousands of times faster than existing SOTA 3D modeling approaches.

#### Outlier analysis

The peptide-binding groove of HLA-A∗02:01 has two primary deep pockets, P2 and P9, which are critical for peptide binding and stabilization.^28^ With a few exceptions (e.g., 2GTW, discussed below), the P2 and P9 pockets commonly anchor the 2nd and 9th residues of the peptide. Consistently, most of the 3D models from SwiftMHC have a backbone structure similar to their X-ray counterpart, with common anchor positions 2 and 9 (Figure 3F). The top outlier for SwiftMHC and PANDORA is 2GTW, of which the X-ray structure has anchor positions 1 and 9, but it is predicted with anchor positions 2 and 9 (Figure 3G). Given that PANDORA uses netMHCpan 4.1 for anchor prediction, and SwiftMHC was trained on PANDORA models, SwiftMHC likely inherits these netMHCpan-based prediction errors, reducing accuracy in such cases.

MHCfold exhibited a notably higher median Cα-RMSD (3.65 Å) than the other methods (0.87–1.32 Å). Although AlphaFold2-FineTune achieved a low median Cα-RMSD, it produced many outliers, including at least two 3D models in which one end of the peptides was positioned outside the MHC groove (Figure 3G).

### SwiftMHC-generated 3D models closely approximate the quality of X-ray structures

In addition to root-mean-square deviation (RMSD), we assessed model quality by evaluating van der Waals clashes, chirality, and backbone dihedral angles.

#### Number of van der Waals clashes

**Figure 4:**
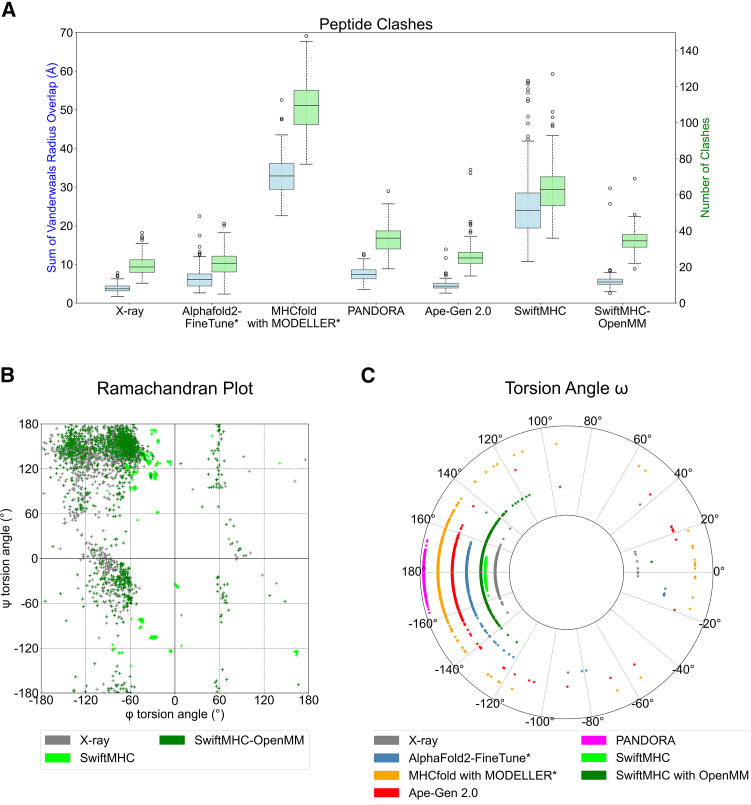
Structural characterizations of SwiftMHC-generated 3D models for the 202 X-ray test cases (A) Distribution of the number of clashes and the sum of their van der Waals radius overlaps per 3D model/structure. Boxplots show the median, interquartile range (IQR), and whiskers extending to the 1.5 IQR; points beyond whiskers represent outliers. (B) Ramachandran plot for the distribution of backbone φ and ψ torsion angles in both SwiftMHC 3D models and the X-ray structures. (C) Distribution of the ⍵ peptide bond torsion angle for all 3D models and X-ray structures.

The OpenMM energy minimization step in SwiftMHC effectively reduces van der Waals clashes (see definition in STAR Methods), which occur when atoms are positioned too closely. Across the 202 test cases, these reductions are comparable to the levels achieved by the other methods (Figure 4A), highlighting the importance of a final molecular dynamics step to ensure the physical realism of the 3D models.

#### Chirality

Chirality in amino acids refers to the property where an amino acid molecule is non-superimposable on its mirror image: L- and D-enantiomers. Chirality plays a crucial role in the structure and function of biomolecules, as the spatial arrangement of groups affects how amino acids interact with other molecules. Except for glycine, amino acids are chiral molecules and the vast majority of amino acids in proteins and enzymes of living organisms are in the L-form. In the 202 test X-ray cases, some amino acids lacked side-chain atoms, which were restored using OpenMM PDBfixer.^29^ However, in some instances, PDBfixer introduced D-form amino acids. Since both SwiftMHC and AlphaFold2-FineTune use these structures as input for model generation, we specifically examined D-form amino acids that were introduced in the models (Table 2). SwiftMHC maintains chirality by rotating and translating local frames of amino acids, as well as placing the chiral hydrogen before executing OpenMM. As a result, it produced L-form amino acids in the generated peptide structures. However, when OpenMM was used for energy minimization, some L-form amino acids were converted to D-form. APE-Gen 2.0, which also relies on OpenMM, exhibited the same issue, and even introduced D-form amino acids in the peptide structure. In contrast, AlphaFold2-FineTune and PANDORA, which do not use OpenMM, did not introduce any D-form amino acids. MHCfold, which predicts individual atomic positions around the chiral Cα atom, introduced a D-form amino acid in one of its models.

**Table 2:** Methods evaluated for amino acid chirality in the predicted structures

Methods	PDBfixed^a^ X-ray structures used as input	OpenMM used	Average number of chiral amino acids per model^b^	Number of D-amino acids introduced in the MHC	Number of D-amino acids introduced in the peptide
MHCfold with MODELLER			174	1	0
AlphaFold2-FineTune	✔		173	0	0
APE-Gen 2.0		✔	263	22	12
PANDORA			263	0	0
SwiftMHC	✔		174	0	0
SwiftMHC-OpenMM	✔	✔	174	9	0

a OpenMM PDBfixer^29^ was used to restore missing side-chain atoms in some of the 202 X-ray structures, which resulted in the introduction of 11 D-form amino acids.

b Some methods model the entire class I MHC protein structure, while others model only the G-domain, leading to differences in the total number of amino acids.

#### Dihedral angles of the peptide backbone

The backbone torsion angles, ϕ (phi) and ψ (psi), define the conformation of a protein’s backbone. These angles are constrained by steric clashes, such as repulsions between side-chain atoms or backbone atoms, which limit the range of energetically favorable conformations a protein can adopt. Validating computational 3D protein models includes verifying that the dihedral angles fall within the naturally observed regions of the Ramachandran plot, as deviations can signal errors in the model. To this end, we generated Ramachandran plots for the X-ray structures and the 3D models produced by all six methods (Figures 4B and S1). The φ and ᴪ torsion angles produced by the DL part of SwiftMHC were restricted to several small areas in the Ramachandran plot (Figure 4B). After energy minimization in OpenMM, these angles were more widespread, and the SwiftMHC distribution became more closely aligned with that of the experimentally determined X-ray structures.

#### *ω* torsion angles and *cis*/*trans* configurations

“*Trans*” and “*cis*” refer to the geometric configuration around peptide bonds, specifically involving the arrangement of the atoms adjacent to the peptide bond (the amide bond between the carbonyl carbon of one amino acid and the amide nitrogen of the next). The *trans* configuration is the most common configuration found in proteins, as it is generally more stable due to less steric hindrance between the side chains of the amino acids. In the *trans* configuration, the dihedral angle around the peptide bond is approximately 180°.

All five methods generated 3D models in which the ω torsion angles do not correspond to those observed experimentally in the X-ray structures (Figure 4C). For instance, in case of PDB: 5SWQ, all methods predicted an asparagine-glycine peptide bond with an ω angle ranging from 160.0° to 200.0° (*trans*), whereas the X-ray structure showed this angle as −0.6° (*cis*). Similarly, SwiftMHC predicted seven glutamate-proline peptide bonds with ω angles between 160.0° and 200.0° (*trans*), while the X-ray structures indicated that these angles fall between −20.0° and 20.0° (*cis*). Notably, OpenMM appears to shift these specific angles outside the *trans* range, approaching 140°. Furthermore, SwiftMHC predicted nearly all ω angles in the *trans* conformation, although the energy minimization step in OpenMM slightly altered these angles in some cases. An extreme case is the isoleucine-proline bond in the SwiftMHC 3D model for 5ENW. This ω angle was almost flipped by OpenMM from −178° (*trans*) to 7° (*cis*).

## Discussion

We introduce SwiftMHC, an attention-based neural network for the rapid and accurate identification of MHC-bound peptides and generating 3D structures. SwiftMHC delivers high-resolution all-atom 3D models with a median Cα-RMSD of 1.19 Å, as well as a remarkable efficiency by processing each case in just 0.009 s (AI mode) to 2.2 s (AI + OpenMM mode) in batch mode on a single A100 GPU. SwiftMHC effectively overcomes the speed bottleneck of structure-based BA predictors while maintaining exceptional accuracy in both BA prediction and 3D structure modeling.

The speed and accuracy of SwiftMHC are attributed to two key innovations: (1) removing the computationally demanding MSA attention module, since MHC structures are conserved and peptides lack evolutionary information, and (2) incorporating physics-derived pMHC-I 3D models into training. While the AI industry often focuses on large, general-purpose models like AlphaFold3,^16^ these approaches demand vast datasets, extensive compute resources, and high energy consumption, both during training and inference, while remaining difficult to optimize. In contrast, SwiftMHC demonstrates that smaller, task-specific networks—when augmented with extensive physics-based synthetic data—can achieve superior efficiency without sacrificing accuracy. This design highlights a sustainable path forward for domain-focused biomolecular modeling.

In addition, we explored whether cross-attention weights could provide interpretable signals for prediction confidence. By mapping attention scores onto the MHC surface (Figure S3), we observed distinct patterns between correct (1HHK, anchors at positions 2 and 9) and incorrect (2GTW, anchors at positions 1 and 9 but predicted as 2 and 9) anchor assignments. In the “correct” case, the model concentrated attention on the P2 pocket, while in the incorrectly predicted case, such localized focus was absent. This suggests that attention distributions may serve as indicators of anchor misprediction, although systematic validation will be required to establish their utility.

As the fastest pMHC-I structure predictor, SwiftMHC facilitates large-scale peptide screening for vaccine development and immunotherapy research. Its high efficiency enhances the ability to identify novel neoantigens from the diverse range of tumor-derived peptide fragments, while providing detailed 3D models for studying molecular interactions and T cell recognition. Furthermore, SwiftMHC’s speed offers the potential to construct the first comprehensive 3D self-peptide-HLA (human MHC) library. Such a library could be pivotal for identifying TCR-therapy peptide targets that differ from self-peptides on T cell-exposed surfaces, ultimately contributing to the development of safer and more effective therapeutic strategies.

This proof-of-concept study focuses on HLA-A∗02:01 9-mers, selected for their prevalence and clinical relevance. Their abundant data and fixed peptide length make it a relatively straightforward case for sequence-based predictors used in comparison. A key direction for future work is to expand SwiftMHC training set to include additional alleles and peptides of varying lengths. Such expansion is expected to enhance SwiftMHC’s generalizability, aligning with the reported 6%–16% performance gains of structure-based methods over sequence-based methods.^11^

Lastly, while optimized for pMHC complexes, our SwiftMHC strategy can be adapted for broader applications, including protein-peptide interactions and CDR3 loop modeling for antibody-antigen and TCR:pMHC-I complexes. The flexibility and the lack of evolutionary signal of peptides and CDR3 loops pose significant challenges for accurate modeling, even for advanced methods like AlphaFold.^30^ Capturing peptide and CDR3 conformational changes upon binding is critical for advancing therapeutic TCR and antibody design as well as our understanding of immune recognition and defense.

### Limitations of the study

This proof-of-concept study has several limitations. First, SwiftMHC is currently restricted to HLA-A∗02:01 9-mer peptides. However, its algorithm is designed to easily incorporate training sets for additional alleles and peptides of varying lengths.

Second, for computational efficiency, we chose to design SwiftMHC to use fixed MHC orientations, not allowing random orientation options. This limitation could be addressed by redesigning the network to incorporate rotation-translation equivariance, such as using backbone frames based on the MHC protein’s intrinsic orientation rather than identity frames.

Third, SwiftMHC does not yet account for peptides with post-translational modifications (PTMs) such as phosphorylation, glycosylation, methylation, and acetylation. Addressing this would require training on datasets with sufficient examples of post-translationally modified structures. While SwiftMHC cannot currently model PTMs directly, it can generate reasonable starting conformations for downstream molecular dynamics (MD) simulations of modified peptides.

Finally, the speed evaluation of SwiftMHC in this study was conducted using a maximum batch size of 64 pMHC cases, optimized for large datasets (∼8,000 cases, as in the BA test datasets), which efficiently filled most batches. However, SwiftMHC’s efficiency may decline when processing very small datasets with only a handful of cases per batch. Additionally, the impact of software initialization time, which may reduce efficiency for smaller datasets, was not explored. Further investigation into time consumption for larger datasets could offer additional insights into its performance efficiency.

## Resource availability

### Lead contact

Requests for further information and resources should be directed to and will be fulfilled by the lead contact, Dr. Li C. Xue (li.xue@radboudumc.nl).

### Materials availability

This study did not generate new unique reagents.

### Data and code availability

• Data: All input data (including PANDORA 3D models, BA data, preprocessed datasets), network models, and results (including torsion angles, clash counts, chiralities, and RMSD and BA predictions) have been deposited at Zenodo and are accessible at https://doi.org/10.5281/zenodo.14968655. This includes utility scripts that have been used to process the data.
• Code: All code for data preprocessing, evaluation, and prediction is available at https://doi.org/10.5281/zenodo.18001720
• Additional information: Any additional information required to reanalyze the data reported in this paper is available from the lead contact upon request.

## Acknowledgments

This project was supported by the 10.13039/501100023452Hanarth Fonds 2022 (NL), the Kika foundation (grant no. 454, NL), NWO-XS (OCENW.XS23.2.130, NL), and the NLeSc grant for the 3D-Vac project (grant ID: NLESC.OEC.2021.008). The computation was partly supported by the 10.13039/100007065NVIDIA Academic Grant Program. We thank SurfSara in the Netherlands for their generous GPU and CPU computing resources (grant numbers EINF2380, EINF-10427, and EINF-11930). We also thank Dr. Dario Marzella for providing the dataset and helpful discussions.

## Author contributions

C.A.B.B. contributed to the design and development of software, experimentation, and writing of the manuscript; G.C. performed manuscript review and editing; C.G. performed manuscript review and code optimization; D.T.R. reviewed and edited the manuscript and participated in consultation on DL-related questions; D.F. reviewed and edited the manuscript and tested the code; Y.J.MA. performed manuscript review and editing and examined possible chirality problems in AlphaFold3-generated models; L.C.X. designed and supervised the project, in addition to reviewing and editing of the manuscript. All authors have reviewed and edited this manuscript.

## Declaration of interests

The authors declare no conflict of interest.

## Declaration of generative AI and AI-assisted technologies in the writing process

During the preparation of this work, the author(s) used ChatGPT (https://chatgpt.com) in order to improve the writing style. After using this tool, the authors reviewed and edited the content as needed and take full responsibility for the content of the publication.

## STAR★Methods

### Key resources table

**Table:** 

REAGENT or RESOURCE	SOURCE	IDENTIFIER
**Deposited data**		

7,726 BA data entries, originating from IEDB with cluster numbers added. In archive: input_data/IEDB-BA-with-clusters.csv	Zenodo	https://doi.org/10.5281/zenodo.14968656
202 X-ray structures originating from PDB, chain identifiers modified, missing atoms added, cluster numbers added. In archive: input_data/PDB-xray-clusters.csv input_data/xray-pdbfixed/	Zenodo	https://doi.org/10.5281/zenodo.14968656
Results of structural analysis of 202 X-ray structures, originating from PDB, chain identifiers modified, missing atoms added, cluster numbers added. In archive: input-data/xray-pdbfixed/clashes.csv input-data/xray-pdbfixed/ramachandran.csv input-data/xray-pdbfixed/omegas.csv input-data/xray-pdbfixed/chirality.csv	Zenodo	https://doi.org/10.5281/zenodo.14968656
Results of comparing all 202 X-ray structures against each other in terms of structural variation. In archive: input-data/xray-pdbfixed/TMalign-all-vs-all-rmsd.csv	Zenodo	https://doi.org/10.5281/zenodo.14968656
The result from Gibbs clustering the 7,726 BA data entries from IEDB into ten clusters. In archive: input_data/cluster-data/	Zenodo	https://doi.org/10.5281/zenodo.14968656
The results from checking the overlap between the 202 X-ray structures in the test set and the training data from AlphaFold2-FineTune and MHCfold. In archive: input-data/overlap/	Zenodo	https://doi.org/10.5281/zenodo.14968656
2,838 single point mutants, found by comparing 7,726 BA data entries from IEDB. In archive: input_data/mutants/	Zenodo	https://doi.org/10.5281/zenodo.14968656
SwiftMHC masks and reference structure, used for preprocessing. In archive: input-data/swiftmhc/HLA-A0201-CROSS.mask input-data/swiftmhc/HLA-A0201-GDOMAIN.mask input-data/swiftmhc/reference-3MRD.pdb	Zenodo	https://doi.org/10.5281/zenodo.14968656
7,726 PANDORA models used for training the 10 SwiftMHC models. In archive: input-data/swiftmhc/pandora-models-for-training-swiftmhc/	Zenodo	https://doi.org/10.5281/zenodo.14968656
7,726 BA data entries, originating from IEDB split by 10 folds (train, validation, test) according to 10 clusters. This was the input data for MHCflurry 2.0 10-fold cross validation. In archive: input-data/mhcflurry/train-fold?csv input-data/mhcflurry/valid-fold?csv input-data/mhcflurry/BA-cluster?csv (test sets)	Zenodo	https://doi.org/10.5281/zenodo.14968656
MHCflurry 2.0 retraining hyperparameters In archive: input-data/mhcflurry/mhcflurry-hyperparameters.yaml	Zenodo	https://doi.org/10.5281/zenodo.14968656
7,726 BA data entries, originating from IEDB, converted to fasta format, representing the peptide sequences. This was the input to netMHCpan 4.1 for evaluating BA prediction quality. In archive: input-data/netmhcpan/IEDB-BA.fasta	Zenodo	https://doi.org/10.5281/zenodo.14968656
7,726 BA data entries, originating from IEDB, in fasta format. This is the input data to MHCfold for evaluating BA prediction quality. In archive: input-data/mhcfold/ba.fa	Zenodo	https://doi.org/10.5281/zenodo.14968656
202 X-ray structures, originating from PDB, in fasta format. This is the input data to MHCfold for evaluating structure prediction quality. In archive: input-data/mhcfold/X-ray.fa	Zenodo	https://doi.org/10.5281/zenodo.14968656
6,057 pMHC-I complexes, preprocessed in a format for AlphaFold2-FineTune to work with, representing 7,726 BA data entries from IEDB. In archive: input_data/alphafold2-finetune/alignments-BA/ input_data/alphafold2-finetune/targets-BA.tsv)	Zenodo	https://doi.org/10.5281/zenodo.14968656
202 pMHC-I complexes preprocessed in a format for AlphaFold2-FineTune to work with, representing 202 X-ray structures from PDB. In archive: alphafold2-finetune/alignments-xray/ alphafold2-finetune/targets-xray.tsv	Zenodo	https://doi.org/10.5281/zenodo.14968656
10 SwiftMHC trained network models, one for each fold. The networks were trained with both structural and BA loss. In archive: network-models/swiftmhc/	Zenodo	https://doi.org/10.5281/zenodo.14968656
10 SwiftMHC trained network models, one for each fold. The networks were trained with only BA loss. In archive: network-models/swiftmhc/	Zenodo	https://doi.org/10.5281/zenodo.14968656
10 SwiftMHC trained network models, one for each fold. The networks were trained with only structural loss. In archive: network-models/swiftmhc/	Zenodo	https://doi.org/10.5281/zenodo.14968656
10 MHCflurry 2.0 trained network model assemblies, one for each fold. The networks were first trained on the training set and then a model was selected on the validation set. In archive: network-models/mhcflurry-retrained/train-fold? network-models/mhcflurry-retrained/valid-fold?	Zenodo	https://doi.org/10.5281/zenodo.14968656
Preprocessed data for SwiftMHC (train, validation, test) for 10 folds. The file format is HDF5. In archive: preprocessed/	Zenodo	https://doi.org/10.5281/zenodo.14968656
Timing results for SwiftMHC evaluations (BA, structure with/without OpenMM). In archive: output-data/swiftmhc/model-ba-cluster?-timings.txt output-data/swiftmhc/model-xray-cluster?-with-openmm-timings.txt output-data/swiftmhc/model-xray-cluster?-without-openmm-timings.txt	Zenodo	https://doi.org/10.5281/zenodo.14968656
Predicted structures by SwiftMHC, with/without OpenMM. In archive: output-data/swiftmhc/3d-models-with-openmm/ output-data/swiftmhc/3d-models-without-openmm/	Zenodo	https://doi.org/10.5281/zenodo.14968656
BA prediction results for SwiftMHC evaluations. In archive: output-data/swiftmhc/predict-ba-cluster?csv	Zenodo	https://doi.org/10.5281/zenodo.14968656
Single point mutant results for evaluation by SwiftMHC. In archive: output-data/swiftmhc/mutants/results-wt-cluster?-evaluated-on-3MRD.csv	Zenodo	https://doi.org/10.5281/zenodo.14968656
Attention weights output by SwiftMHC models for peptides of 1HHK and 2GTW. In archive: output-data/swiftmhc/attention-weights/	Zenodo	https://doi.org/10.5281/zenodo.14968656
Results of comparing SwiftMHC predicted structures with their X-ray counterparts. In archive: output-data/swiftmhc/CA-rmsd-without-openmm.csv output-data/swiftmhc/CA-rmsd-with-openmm.csv	Zenodo	https://doi.org/10.5281/zenodo.14968656
Results of analysing SwiftMHC predicted structures. In archive: output-data/swiftmhc/clashes-with-openmm.csv output-data/swiftmhc/clashes-without-openmm.csv output-data/swiftmhc/chirality-with-openmm.csv output-data/swiftmhc/chirality-without-openmm.csv output-data/swiftmhc/omegas-with-openmm.csv output-data/swiftmhc/omegas-without-openmm.csv output-data/swiftmhc/ramachandran-with-openmm.csv output-data/swiftmhc/ramachandran-without-openmm.csv	Zenodo	https://doi.org/10.5281/zenodo.14968656
SwiftMHC predicted structures from network models trained without BA loss. In archive: output-data/swiftmhc/3d-models-with-openmm/	Zenodo	https://doi.org/10.5281/zenodo.14968656
Results of comparing SwiftMHC predicted structures to their X-ray counterparts. The structures were predicted by network models trained without BA loss. In archive: output-data/swiftmhc/CA-rmsd-with-openmm.csv	Zenodo	https://doi.org/10.5281/zenodo.14968656
Results from SwiftMHC BA prediction, from network models trained without structural loss. In archive: output-data/swiftmhc/BA-cluster?csv	Zenodo	https://doi.org/10.5281/zenodo.14968656
netMHCpan 4.1 timing results from processing 7,726 pMHC-I cases. In archive: output-data/netMHCpan/IEDB-BA-time.txt	Zenodo	https://doi.org/10.5281/zenodo.14968656
netMHCpan 4.1 BA prediction results from processing 7,726 pMHC-I cases. In archive: output-data/netMHCpan/IEDB-BA-results.tsv output-data/netMHCpan/IEDB-BA-results.txt	Zenodo	https://doi.org/10.5281/zenodo.14968656
Timings for MHCflurry 2.0 cross-validation network models to evaluate each of 10 clusters from 7,726 IEDB BA data entries. In archive: output-data/mhcflurry-crossvalidation/time_cluster?txt	Zenodo	https://doi.org/10.5281/zenodo.14968656
BA predictions for MHCflurry 2.0 cross-validation network model for each of 10 clusters from 7,726 IEDB BA data entries. In archive: output-data/mhcflurry-crossvalidation/result-cluster?csv	Zenodo	https://doi.org/10.5281/zenodo.14968656
Timings for MHCflurry 2.0 (original training) for each of 10 clusters from 7,726 IEDB BA data entries. In archive: output-data/mhcflurry-original/time_cluster?txt	Zenodo	https://doi.org/10.5281/zenodo.14968656
BA predictions for MHCflurry 2.0 (original training) for each of 10 clusters from 7,726 IEDB BA data entries. In archive: output-data/mhcflurry-original/result_cluster?csv	Zenodo	https://doi.org/10.5281/zenodo.14968656
Results of comparing the BA predictions by MHCflurry 2.0 (original training) to the BA predictions of SwiftMHC. In archive: output-data/ba-true-vs-swiftmhc-vs-mhcflurry.csv	Zenodo	https://doi.org/10.5281/zenodo.14968656
PANDORA models with best energy scores, predicting the structures of 202 pMHC-I cases, originating from PDB. In archive: output-data/pandora/best/	Zenodo	https://doi.org/10.5281/zenodo.14968656
Results of comparing 202 best PANDORA models to their PDB X-ray counterpart. In archive: output-data/pandora/best-models-CA-rmsds.csv	Zenodo	https://doi.org/10.5281/zenodo.14968656
Results of structural evaluation of 202 best PANDORA models. In archive: output-data/pandora/best-models-clashes.csv output-data/pandora/best-models-omegas.csv output-data/pandora/best-models-ramachandran.csv output-data/pandora/best-models-chirality.csv	Zenodo	https://doi.org/10.5281/zenodo.14968656
Timing result for PANDORA to process 202 cases in 32 threads. In archive: output-data/pandora/timings.txt	Zenodo	https://doi.org/10.5281/zenodo.14968656
APE-Gen 2.0 models with best energy scores, predicting the structures of 202 pMHC-I cases, originating from PDB. In archive: output-data/apegen2/best/	Zenodo	https://doi.org/10.5281/zenodo.14968656
APE-Gen 2.0 timings for processing each of 202 pMHC-I cases. In archive: output-data/apegen2/????-time.txt	Zenodo	https://doi.org/10.5281/zenodo.14968656
Results of comparing 202 APE-Gen 2.0 models to their X-ray counterparts. In archive: output-data/apegen2/best-models-CA-rmsds.csv	Zenodo	https://doi.org/10.5281/zenodo.14968656
Results of structural evaluation of 202 best APE-Gen 2.0 models. In archive: output-data/apegen2/best-models-clashes.csv output-data/apegen2/best-models-ramachandran.csv output-data/apegen2/best-models-omegas.csv output-data/apegen2/best-models-chirality.csv	Zenodo	https://doi.org/10.5281/zenodo.14968656
Results of MHCfold structural prediction of 202 pMHC-I cases, corresponding to X-ray structures from the PDB. In archive: output-data/mhcfold/results-xray/????_mhcfold_full_relaxed.pdb	Zenodo	https://doi.org/10.5281/zenodo.14968656
Timing of MHCfold to process 202 pMHC-I cases, corresponding to X-ray structures from the PDB. In archive: output-data/mhcfold/X-ray.time.txt	Zenodo	https://doi.org/10.5281/zenodo.14968656
Results of comparing 202 MHCfold structural predictions to their X-ray counterparts. In archive: output-data/mhcfold/results-xray/CA-rmsds-full-relaxed.csv	Zenodo	https://doi.org/10.5281/zenodo.14968656
Structural evaluation of 202 MHCfold pMHC-I models. In archive: output-data/mhcfold/results-xray/clashes_full_relaxed.csv output-data/mhcfold/results-xray/ramachandran_full_relaxed.csv output-data/mhcfold/results-xray/omegas_full_relaxed.csv output-data/mhcfold/results-xray/chirality_full_relaxed.csv	Zenodo	https://doi.org/10.5281/zenodo.14968656
MHCfold BA prediction results for 7,726 entries, originating from IEDB. In archive: output-data/mhcfold/ba_classification_results.csv	Zenodo	https://doi.org/10.5281/zenodo.14968656
Timing for MHCfold to predict BA for 7,726 entries, originating from IEDB. In archive: output-data/mhcfold/ba.time.txt	Zenodo	https://doi.org/10.5281/zenodo.14968656
AlphaFold2-FineTune structural predictions for 202 pMHC-I cases, originating from PDB. In archive: output-data/alphafold2-finetune/xray-models-renamed/	Zenodo	https://doi.org/10.5281/zenodo.14968656
Timing for AlphaFold2-FineTune to predict 202 pMHC-I structures. In archive: output-data/alphafold2-finetune/xray-model-timings.txt	Zenodo	https://doi.org/10.5281/zenodo.14968656
Comparison of the AlphaFold2-FineTune 202 predicted structures to their X-ray counterparts. In archive: output-data/alphafold2-finetune/xray-models-CA-rmsd.csv	Zenodo	https://doi.org/10.5281/zenodo.14968656
Structural evaluation of 202 AlphaFold2 predicted pMHC-I structures. In archive: output-data/alphafold2-finetune/xray-models-clashes.csv output-data/alphafold2-finetune/xray-models-ramachandran.csv output-data/alphafold2-finetune/xray-models-omegas.csv output-data/alphafold2-finetune/xray-models-chirality.csv	Zenodo	https://doi.org/10.5281/zenodo.14968656
AlphaFold2-FineTune BA predictions for 6,057 pMHC-I cases, representing 7,726 BA data entries from IEDB. In archive: output-data/alphafold2-finetune/ba-model-result.tsv	Zenodo	https://doi.org/10.5281/zenodo.14968656
Timing for AlphaFold2-FineTune to predict 6,057 pMHC-I cases, representing 7,726 BA data entries from IEDB. In archive: output-data/alphafold2-finetune/ba-model-timings.txt	Zenodo	https://doi.org/10.5281/zenodo.14968656

**Software and algorithms**		

SwiftMHC 1.0.0	This paper	https://doi.org/10.5281/zenodo.18001720
MHCflurry 2.0	O’Donell et al.^9^	https://github.com/openvax/mhcflurry
NetMHCpan 4.1	Reynisson et al.^10^	https://services.healthtech.dtu.dk/services/NetMHCpan-4.1/
MHCfold	Aronson et al.^20^	https://github.com/dina-lab3D/MHCfold
APE-Gen 2.0	Fasoulis et al.^15^	https://github.com/KavrakiLab/Ape-Gen2.0/
PANDORA v2.0.0	Parizi et al.^14^	https://github.com/x-lab-3D/PANDORA
AlphaFold2-FineTune	Motmaen et al.^18^	https://github.com/phbradley/alphafold_finetune
Gibbs Cluster 2.0	Ellis et al.^23^	https://services.healthtech.dtu.dk/services/GibbsCluster-2.0/
Profit 3.1	Martin and Craig^31^	https://mybiosoftware.com/profit-3-1-protein-squares-fitting.html
TM-align 1	Zhang et al.^32^	https://launchpad.net/ubuntu/resolute/amd64/tm-align/20190822+dfsg-3ubuntu1
Pymol 3.1.0 (open source)	DeLano^33^	https://anaconda.org/conda-forge/pymol-open-source
MRS 6	Hekkelman and Vriend^5^	https://mrs.cmbi.umcn.nl/

### Method details

#### Data composition

In this study, we focus on HLA-A∗02:01 alleles and 9-mer peptides. For this, 7,726 BA data points were collected from IEDB^27^ and 202 X-ray structures were collected from the PDB.^12^ OpenMM PDBfixer^29^ was used to fill in missing heavy atoms in these X-ray structures in cases where a residue was incomplete. To critically evaluate the generalizability of our predictor and to prevent data leakage, BA and X-ray data were merged together and Gibbs clustered^34^ based on their peptide sequences. This was performed using GibbsCluster 2.0, with a cluster similarity penalty (λ) set to 0.8 and a small cluster weight (σ) set to 5. The data points were separated into 10 clusters, each of which was used for testing SwiftMHC using a leave-one-cluster-out cross-validation principle (Figure 3E).

From the left-out cluster of each fold, the BA data points were used to evaluate the BA prediction quality, while the X-ray structures from that same cluster were used for evaluating structure prediction quality. The BA data points of the remaining 9 clusters were used for training (90% of data) and validation (10%). X-ray crystallographic data were excluded from the training and validation datasets to assess the network’s 3D modeling performance when trained solely on physics-based 3D model data, though including X-ray data would likely enhance our performance.

Since SwiftMHC requires structural data for training, we generated 3D models for each pMHC-I complex in the BA dataset using PANDORA. For each pMHC-I complex, the 3D model with the lowest molecular probability density function score (molpdf) energy score was selected to be used as input to SwiftMHC.

For the evaluation of BA predictions, we initially used the MHC structures from the corresponding PANDORA models as input to the network model. We later reasoned that using these PANDORA-derived MHC structures instead of the reference experimental structures could introduce a bias due to minor structural variations. However, comparative tests using reference MHC structures showed negligible differences in performance metrics. Therefore, we retained the PANDORA-derived results. For evaluating structure prediction, the MHC structures of the corresponding X-ray structures were used as input to the network model. For evaluating the mutants (Figure S2F) this inconsistency was corrected, and the reference MHC structure from PDB entry 3MRD was used as input.

#### Data preprocessing

SwiftMHC gradually updates the peptide structure from identity frames (Suppl. mat. Algorithm 4), that is, the starting positions of all peptide residues are at the origin and their N-Cα-C plane overlaps so that they all have the same orientation. This design requires all input structural data in the same orientation, so we superimposed the pMHC-I complex of all X-ray structures and PANDORA 3D models on a reference MHC structure (PDB ID: 3MRD) before training and testing. The reference structure was prepared by isolating the G-domain and placing the geometric center of the MHC binding groove at the origin. All other structures or PANDORA 3D models were structurally aligned to this reference using the PyMOL align command^33^ with its default settings: 2.0 Å as outlier rejection cutoff, 5 outlier rejection cycles and using BLOSUM62 for sequence alignment.

Additionally, some values were pre-calculated from the aligned pMHC-I structures, so that SwiftMHC can quickly access them. Those variables were: *ground truth frames*, *ground truth torsion angles*, *MHC proximity matrix*, *peptide and MHC amino acid sequences*, *residue masks* and *ground truth BA*. See Suppl. mat. Subsection 1.2 for details about these variables.

#### Software and libraries used

SwiftMHC was developed using Python 3.12 and implemented with the PyTorch 2.0.1 library^35^ for deep learning functionalities. OpenFold 1.0.0^36^ was utilized for key tasks such as FAPE and torsion loss calculations, deriving frames from atomic coordinates, reconstructing atomic coordinates from predicted frames, and performing frame operations. The design of SwiftMHC’s IPA modules was also partially inspired by OpenFold code. For handling PDB files, BioPython 1.8.4^37^ was employed for parsing and representation. Structural refinements were performed using OpenMM 8.1.1.^4^

#### Training

10 SwiftMHC network models were trained for each of the 10 peptide clusters, using 10 distinct training datasets. The models were trained in PyTorch^35^ 2.3.1 using the Adam optimizer, with a batch size of 16, float32 precision and a learning rate of 10^−3^. To avoid large loss spikes, gradient norms are clipped to 0.5 at all times. Training is done in two phases, where early stopping can end a phase when stopping criteria are met. In each phase, the early stopping conditions are reset and the model with the lowest validation loss is selected as the initial state in the next training phase.

**Figure 5:**
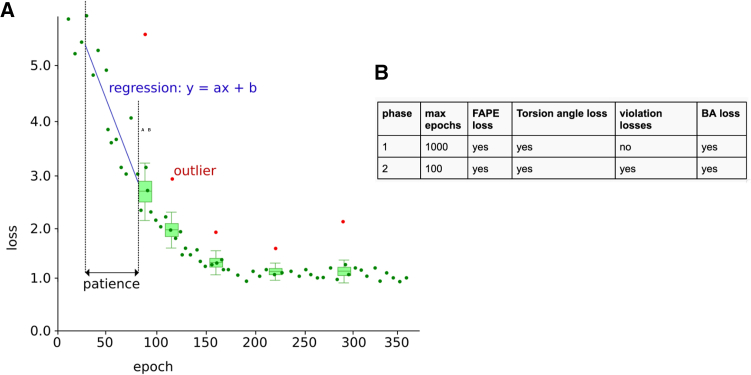
Training method (A) Early stopping criteria. An example scatter loss plot is shown. Dots (dark green) indicate loss values. Boxplots (light green) determine which dots are outliers (red) within the “frame of patience” (50 epochs). The best line (blue) is drawn between the non-outlier values by means of linear regression within the “frame of patience.” The parameters of this line are the slope (a) and offset (b). (B) Parameter settings for each training phase. Columns 3–6 indicate which of the four loss terms are included in the back propagation during each phase (supplementary algorithm 9, lines 29–43). Early stopping is turned on for both phases.

The training process consists of two distinct phases, each with specific settings (Figure 5B). The goal of the first phase is to enable the model to learn structure prediction and reduce peptide Cɑ-RMSD from approximately 8 Å to 1–2 Å. During this phase, FAPE and torsion loss are included to guide atomic positions and torsion angles toward those of the true structure. Simultaneously, the BA loss term is incorporated to train the BA prediction module and to speed up the convergence of the 3D structure training. While this phase includes a high maximum epoch count (1000 epochs), an early stopping condition is applied to conclude the phase early if no more improvement in performance is observed.

In the second phase, the model’s structure prediction is fine-tuned by introducing violation loss terms during backpropagation (Method S1, Algorithm 10). This phase resembles the fine-tuning phase in AlphaFold27 and the associated loss terms encourage interatomic distances, bond lengths and bond angles to approach standard literature values.

During training, an early stopping mechanism is applied to end a training phase and skip to the next phase or to end the training when the conditions are met in the final phase. The conditions for early stopping are based on loss values that are calculated on a validation dataset.

After each epoch the algorithm evaluates the loss values within a range of epochs (i.e., the patience, Figure 5A) unless the number of epochs passed in that training phase is less than the patience value. We configured this patience value to be 50. For example, at epoch 50 we name the range 0–50 the *Frame of Patience*. At epoch 51, this frame shifts to the range 1–51 and so on.

Sometimes the high spikes of losses interrupts the early stopping. So we need to first smooth out the loss values within each Frame of Patience. Within each Frame of Patience the algorithm searches for outliers. Whether a loss value is an outlier or not is determined by the interquartile range (IQR). A loss value is considered an outlier if it lies more than 1.5 times the IQR above the third quartile (Q3) or more than 1.5 times the IQR below the first quartile (Q1). These outliers are replaced by the median value within the Frame of Patience, so that they cannot block early stopping.

After replacing the outliers with median values, linear regression is performed to find the best straight line through the loss values within a Frame of Patience. The algorithm stops a training phase early if the slope of this line is nearly horizontal and its value lies between −1 × 10^−4^ and 1 × 10^−4^, indicating the predictive performance on the validation set is not improving any more.

#### Post processing the resulting structures

Once a pMHC-I structure is predicted and PDB to disk writing is enabled, SwiftMHC optionally runs OpenMM^29^ to minimize its energy using an amber99sb force field. To prevent chirality issues in non-glycine backbones, the hydrogen atom attached to the chiral Cα atom is added by SwiftMHC before running OpenMM. All other hydrogen atoms are added by OpenMM.

#### SOTA methods

We compared SwiftMHC against several SOTA methods (Table 1) on BA prediction and/or structure prediction qualities. Five methods were compared for their speed and BA prediction quality: SwiftMHC, AlphaFold2-FineTune,^18^ MHCflurry 2.0^9^ (both original and retrained models, see additional resources for the download URL), netMHCpan 4.1 and MHCfold.^20^ The AUC was used as a BA quality metric. We classified a peptide as binding to its MHC if IC_50_ or K_d_ was below 500 nM and as non-binding otherwise. Six methods were compared for reproducing the 202 X-ray structures: SwiftMHC, SwiftMHC-OpenMM, AlphaFold2-FineTune,^18^ PANDORA,^14^ APE-Gen 2.0^15^ and MHCfold.^20^

*AlphaFold2-FineTune* is a modified version of AlphaFold2, where the weights and biases of the network model have been fine-tuned using pMHC BA and X-ray pMHC structural data. AlphaFold2-FineTune achieves faster processing times than standard AlphaFold2 by omitting template searching and multiple sequence alignments. Instead, it requires the user to provide a query-to-template alignment. A second reason for the increased speed of AlphaFold2-FineTune compared to the standard AlphaFold2 tool is that it does not refine its output structures using an Amber forcefield in OpenMM, which is typically employed for energy minimization in the standard AlphaFold2 pipeline.

We used the 202 X-ray HLA-A∗02:01 9-mer structures as templates for AlphaFold2-FineTune. For selecting templates, the Gibbs clustering was used. This means that for each target peptide from a specific cluster, the pMHC-I X-ray structures from the other nine clusters were selected as the template set. To create MHC alignments as input for AlphaFold2-FineTune, the target G-domain MHC sequences were aligned to the template MHC sequences using the BioPython pairwise alignment tool^37^ with default settings (Needleman-Wunsch algorithm, match score 1, mismatch score 0, gap scores 0). For peptide alignment, the 9-mer peptides were aligned in a one-to-one manner (i.e., position one aligned with position one, position two with position two, and so on). Other components of the X-ray structures, such as β2-microglobulin and the ɑ3 domain, were excluded from the alignments. The time to make these alignments was not included in the computation time of AlphaFold2-FineTune. The quality of structural predictions was assessed by comparing the 202 X-ray HLA-A∗02:01 9-mer structures against 3D models predicted by AlphaFold2-FineTune. The total runtime required to process these 202 entries was documented.

To assess the quality of BA predictions, we compared 7,726 experimental BA values against predictions generated by AlphaFold2-FineTune. Due to redundancy in the pMHC-I sequences (sometimes there are multiple BA values per complex) and the computational demands of running AlphaFold2-FineTune, we ran AlphaFold2-FineTune on the 6,057 non-redundant data. The total runtime required to process these 6,057 entries was documented. This evaluation aimed to measure AlphaFold2-FineTune’s ability to differentiate true binders (experimental K_d_ or IC_50_ < 500 nM) from true non-binders, following a similar approach to that described in.^18^ For each pMHC-I entry, the negative mean of the MHC-to-peptide and peptide-to-MHC position-aligned error (PAE) values was used as a predictive score. These scores were compared with experimental BA values to calculate the AUC for each cluster. The prediction scores that correspond to the repeated sequence entries were replicated, ensuring each experimental BA value was compared to its corresponding prediction score.

*MHCfold*^20^ is a deep learning tool that first predicts the structures of both the peptide and the MHC molecule from their sequences using convolutional neural networks (CNNs). It then employs a modified version of multi-headed attention along with a structure-derived distance matrix, similar to IPA, to predict BA as either binding or non-binding. To evaluate the BA and structural prediction quality of the MHCfold algorithm, we used the MHC G-domain sequences along with corresponding peptide sequences as inputs. MHCfold’s reliance on side-chain modeling and PDB generation may slow down processing. For fair comparison with SwiftMHC-BA, these features were disabled during BA evaluation (referred to as “MHCfold-BA”). We used MHCfold to predict BA for all 7,726 data points and calculated the AUC based on the predictions. The total runtime was measured. Additionally, MHCfold was used to generate pMHC structures for all 202 X-ray complexes. For this. MHCfold was set to predict both the 3D structure and BA and to use MODELLER for reconstructing the side chains. The total runtime for creating the 202 3D models, including the time required for MODELLER, was measured. The resulting 3D models were subsequently evaluated.

*PANDORA* is a 3D modeling approach, specialized to predict the structure of pMHC complexes for both class I and II. It does so by using a database of MHC templates and a multiple sequence alignment of their conserved domains. It uses homology modeling to build the model and keeps the peptide’s anchors restrained. To evaluate the performance of PANDORA^14^ structure predictions, we made it generate pMHC-I 3D models for each of the 202 available X-ray structures. PANDORA was configured to output 20 3D models per case (the default setting) and the 3D model with the lowest molpdf score was selected. To prevent data leakage, templates containing peptides identical to those in the target 3D model were excluded from the 3D modeling process. To properly measure the process time consumption, PANDORA was executed within a multiprocessing pool configuration with 32 cores. The total processing time required for the pool to complete all 202 3D models was recorded.

*ApeGen 2.0* is a tool that generates an ensemble of peptide-MHC conformations within a chosen number of iterations. In each iteration, it first searches the Protein DataBank (PDB) for a suitable MHC template, then anchors the atoms of the first and last two residues of the given peptide as in the template. Between these anchor positions, it samples for peptide backbone conformations that geometrically fit best using Random Coordinate Descent (RCD).^38^ On the resulting backbone conformations, OpenMM PDBFixer^29^ samples for side chains to complete the structure. Finally SMINA^39^ energy minimization is performed on both the peptide and MHC to fix steric clashes. The best resulting structure may be used as input for the next iteration.

To evaluate the structure prediction performance of APE-Gen 2.0,^15^ we employed this software to 3D model the pMHC complex for each of the 202 available X-ray structures. APE-Gen 2.0 was set to output 20 3D models per case (the default setting) and the 3D model with the lowest APE-Gen 2.0 Affinity score was selected. To prevent data leakage, templates containing identical peptides to those in the target 3D model were excluded from the set. By default, an APE-Gen 2.0 process runs in a Docker container, using 8 CPU cores to pool the RCD and SMINA computations. For comparing APE-Gen 2.0’s runtime with other SOTA methods on the same software and hardware settings, we ran APE-Gen 2.0 outside a Docker container as a single process with 32 cores. The total processing time for APE-Gen 2.0 to generate all 202 3D models was recorded. These resulting 3D models were subsequently evaluated.

*MHCflurry 2.0*^9^ is a SOTA sequence-based BA predictor which consists of an ensemble of MLPs. We retrained MHCflurry 2.0 on our 7,726 BA data points by a 10-fold leave-one-cluster-out cross-validation approach for each of the 10 peptide clusters (Figure 3E). The retraining process was carried out using the allele-specific scripts, provided by MHCflurry 2.0. In each iteration, one cluster was held out as the test set, while the remaining nine clusters were used to create a training set (90%) and a network model selection set (10%). The selected network models were subsequently used to predict BA for the peptides from the remaining cluster. The predicted BA values from MHCflurry 2.0 were compared to experimental BA values (binding or non-binding) by calculating AUC. The processing time was measured.

### Quantification and statistical analysis

#### Computational efficiency

For speed purposes, several design choices were made for SwiftMHC. Since the MHC structure remains fixed, its backbone frames and distance matrix can be precalculated and reused throughout the prediction process. This avoids repeated computations. The preprocessed data was stored in an HDF5 file format to facilitate quick access and efficient data management. For an efficient cross attention computation, MHC residues that do not lie close to the peptide are masked out, meaning that they do not contribute to any operation in that module (Figure S4E). PDB structures are generally only written to disk on demand and never during training. In addition, we replaced the traditional 3 × 3 rotation matrix operations with quaternion operations, reducing numerical computations and improving efficiency.

The *time consumption* of executing a fully trained SwiftMHC network model was measured on a single NVIDIA A100 GPU (40 GiB HBM2 memory with 5 active memory stacks per GPU) and 32 Intel Xeon Platinum 8360Y CPUs and limited to using 40 GiB 3.2 GHz DDR4 memory. We made SwiftMHC allocate 16 worker processes to read input data and 16 builder processes to write out structural data in PDB format. For comparing speed, SOTA methods were executed on the same hardware configuration as SwiftMHC, except for AlphaFold2-FineTune, which required a 100 GiB memory limit instead of 40 GiB. We measured the exact time duration of every method to complete with one-hundredth of a second precision.

#### The training data overlap between AlphaFold2-FineTune, MHCfold and our test data

We did not retrain AlphaFold2-FineTune, because of limited time and computational resources. Instead, we utilized a pretrained network model obtained from the publicly available source (see additional resources for the download URL). We identified that the 202 X-ray structures in our test set represent 118 unique pMHC-I’s of which 34 were part of AlphaFold2-FineTune’s training dataset. Moreover, AlphaFold2-FineTune was fine-tuned from the AlphaFold2 model, which had been trained on PDB entries deposited before the 30th of April 2018,^18^ which dates after the deposition date of 98 of those 118 unique pMHC-I’s. Therefore it is likely that these structures were included in AlphaFold2’s training dataset. IThat means that in total 105 of the 118 unique pMHC-I’s were used to train AlphaFold2-FineTune (see also Figure 3C).

MHCfold could not be retrained for this evaluation due to the absence of a training script. Therefore, we utilized the pretrained network model available from the public source (see additional resources for the download URL) to generate MHCfold predictions for the evaluation. For structural prediction, this model was trained on 431 unique pMHC-I’s deposited before November of 2021 with resolution higher than 3.5 Å.^20^ This includes all 118 unique pMHC-I’s in our X-ray test dataset (Figure 3C) and therefore it is likely that these structures were included in MHCfold’s training dataset.

For BA prediction, both the AlphaFold2-FineTune and MHCfold models were reportedly trained on the netMHCpan 4.1 training set^18^^,^^20^ which incorporates pMHC binding data from the IEDB. Since our datasets were also derived from the IEDB, it is plausible that some, if not all, of our test data was included in the training sets of these two pretrained models.

These overlaps could have inflated the results of AlphaFold2-FineTune and MHCfold.

#### Evaluation of BA prediction

We evaluated the performance of each BA predictor using the area under the receiver operating characteristic curve (AUC) as the evaluation metric. To meet the binary classification requirements of AUC, we categorized peptides as binding to their MHC when their ground truth IC_50_ or Kd values were below 500 nanomolar (nM) and as non-binding otherwise.

#### Evaluation of structure prediction

To calculate *Cɑ-RMSD*, all the 3D models were superposed in ProFit^31^ to the MHC G-domains of the corresponding X-ray structures. To account for structural variations, the two most N-terminal residues were omitted from the G-domain superposition due to their absence in some structures. Similarly, the C-terminal residue was excluded from the superposition analysis because its orientation varies significantly across different X-ray structures. The remaining portion of the HLA-A∗02:01 sequence, encompassing the amino acids (IMGT numbering 3–179) was utilized for superposition. ProFit was used to calculate RMSD for the peptide Cα atoms to quantify the differences between the predicted 3D models and their corresponding X-ray structures. This analysis was performed for each 3D model across all methods to identify which 3D models exhibited the greatest similarity to their respective X-ray counterparts.

*Chiralities* were determined per amino acid from the positions of the N, C and Cɑ atoms around the Cα atoms using Numpy.

*Ramachandran plots* were generated by calculating the backbone ψ and φ torsion angles for each residue in the peptide using BioPython.^37^

*⍵ torsion angles* of the peptide typically approximate 180°. To verify this, the angles were calculated for each peptide bond across all 3D structures using NumPy. The distributions of ω angles from the predicted 3D models and the X-ray structures were plotted to identify any differences.

*van der Waals clashes* are considered energetically unfavorable and should be minimal. To identify overlaps between the van der Waals radii of non-bonded atom pairs in the peptide, the distances between atoms in the 3D structures were calculated using NumPy. A clash was defined as occurring when two atomic centers were closer than the sum of their van der Waals radii, with exceptions made for 1) protons for which the position is usually determined by calculation, not prediction; 2) for atoms within the same residue, as those are usually either part of a ring system or connected to each other with zero, one or two atoms in between; 3) for the backbone atoms of two connected residues; 4) two connected residues where one is proline, of which the side chain is connected to the backbone; 5) two cysteines that can be disulfide bonded. Any other pair of atoms that were positioned too close to each other was considered clashing. The number and severity of clashes were compared across all 3D models.

### Additional resources

Download URL for AlphaFold2-FineTune pretrained network model: https://files.ipd.uw.edu/pub/alphafold_finetune_motmaen_pnas_2023/datasets_alphafold_finetune_v2_2023-02-20.tgz
Download URL for MHCFold pretrained network model: https://github.com/dina-lab3D/MHCfold/blob/main/v7_date_5_9_2022.zip
Download URL for MHCflurry 2.0 pretrained network model: https://github.com/openvax/mhcflurry/releases/download/pre-2.0/models_class1_presentation.20200611.tar.bz2

## Supplemental information

Document S1. Figures S1–S7 and Method S1

Document S2. Article plus supplemental information
