# Supplementary material for: A high-speed attention network for MHC-bound peptide identification and 3D modeling
Source: Cell Rep Methods. 2026 Mar 31;6(4):101364. doi: 10.1016/j.crmeth.2026.101364 (PMC13106973; doi:10.1016/j.crmeth.2026.101364)
Supplement: Document S2. Article plus supplemental information [file mmc2.pdf]

Cell Reports
Methods

Article

A high-speed attention network for MHC-bound peptide identification and 3D modeling

Graphical abstract

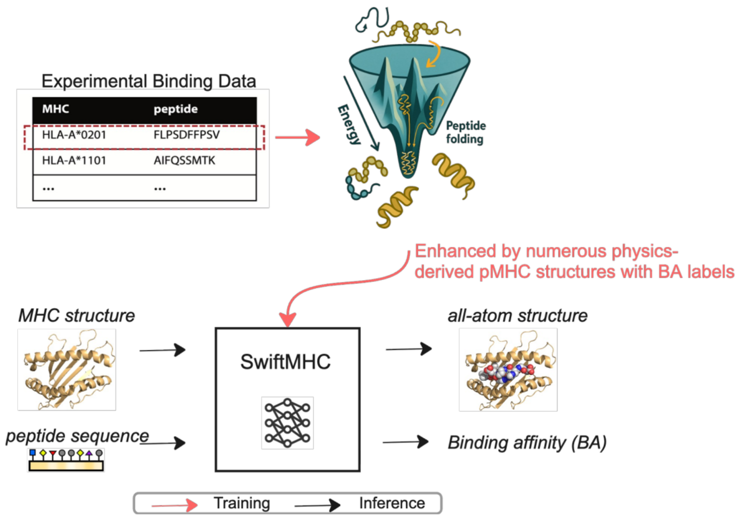

Authors

Coos A.B. Baakman, Giulia Crocioni, Cunliang Geng, Daniel T. Rademaker, David Frühbuß, Yannick J.M. Aarts, Li C. Xue

Correspondence

li.xue@radboudumc.nl

In brief

Baakman et al. introduce SwiftMHC, an ultra-fast AI method that predicts peptide-immune molecule interactions and their 3D structures. The framework enables rapid screening of potential cancer therapy targets, helping bridge the gap between computational speed and structural insight.

Highlights

- SwiftMHC predicts 3D pMHC structures and affinities in milliseconds
- A small, physics-informed model outperforms larger models in data-scarce tasks
- Potentially enables 3D TCR cross-reactivity assessment using structural models

Baakman et al., 2026, Cell Reports Methods 6, 101364
April 20, 2026 © 2026 The Author(s). Published by Elsevier Inc.
https://doi.org/10.1016/j.crmeth.2026.101364

CellPress

CellPress
OPEN ACCESS

Article

A high-speed attention network for MHC-bound peptide identification and 3D modeling

Coos A.B. Baakman,[1] Giulia Crocioni,[2] Cunliang Geng,[2] Daniel T. Rademaker,[3,4,5] David Frühbuß,[1,5] Yannick J.M. Aarts,[6] and Li C. Xue[1,7,*]

[1]Medical BioSciences Department, Radboud University Medical Center, 6525 GA Nijmegen, the Netherlands
[2]Netherlands eScience Center, 1098 XH Amsterdam, the Netherlands
[3]Biosystems Data Analysis, University of Amsterdam, 1090 GE Amsterdam, the Netherlands
[4]HIMS-Biocat, University of Amsterdam, Science Park 904, 1098 XH Amsterdam, the Netherlands
[5]Amsterdam Machine Learning Lab, University of Amsterdam, Science Park 900, 1098 XH Amsterdam, the Netherlands
[6]Adaptation Physiology Group, Department of Animal Sciences, Wageningen University and Research, PO Box 338, 6700 AH, Wageningen, the Netherlands
[7]Lead contact
*Correspondence: li.xue@radboudumc.nl
https://doi.org/10.1016/j.crmeth.2026.101364

MOTIVATION Identifying tumor-derived peptides that stably bind patient MHC molecules and elicit T cell responses is fundamental to developing effective and safe immunotherapies. However, progress in this area remains limited by two key challenges: (1) the scarcity of experimentally characterized peptide-MHC (pMHC) complexes across diverse alleles and (2) the computational cost of accurate 3D modeling. Sequence-based predictors enable large-scale screening but are limited in their ability to generalize beyond well-studied alleles or infer structural determinants of immunogenicity. Structure-based methods, by contrast, can directly capture the physicochemical features that shape TCR recognition, yet their use is restricted by slow inference times. Bridging this gap between speed and structural accuracy is essential not only for scalable peptide-MHC binding prediction but also for understanding how subtle 3D surface differences between self- and non-self-peptides influence immune recognition and therapy safety.

SUMMARY

We developed SwiftMHC, an ultra-fast and accurate structure-based framework for peptide-MHC (pMHC) modeling and binding affinity prediction. Using task-specific deep learning trained on physics-derived synthetic data, SwiftMHC predicts pMHC binding affinities in 0.009 s per case on a single A100 GPU when running in batch mode, offering improved speed compared with leading sequence-based tools such as netMHCpan and MHCflurry while maintaining competitive accuracy. In addition, SwiftMHC generates all-atom 3D pMHC structures with a median Cα-RMSD of 1.32 Å against crystallographic benchmarks, matching or exceeding state-of-the-art methods such as AlphaFold2-finetune but at a lower computational cost. Optimized for HLA-A*02:01 9-mer peptides but readily extensible to other alleles, SwiftMHC unites structural insight with high-throughput scalability to accelerate safe and effective epitope discovery in cancer immunotherapy.

INTRODUCTION

Advances in understanding T cell immunity have led to breakthroughs in cancer immunotherapy, where the immune system is trained to recognize and destroy cancer cells, offering a promising alternative to traditional treatments with fewer side effects.[1] T cells are activated when the T cell receptor (TCR) recognizes tumor-specific peptides presented on the tumor cell surface by the class I major histocompatibility complex (MHC-I) proteins (Figure 1A for molecular details). However, these therapies face challenges, including high costs, long development time, and toxicity risks.[1,2] Currently, the limited number of available target peptides is one of the major bottlenecks of cancer immunotherapies.[3]

Many predictive methods have been developed to identify MHC-binding peptides,[6,7] which have recently made significant contributions to cancer immunotherapy designs.[8] State-of-the-art (SOTA) tools, such as MHCflurry 2.0[9] and netMHCpan 4.1,[10] use both the sequence of the MHC-binding groove and the peptide sequence as input. However, these sequence-based approaches have notable limitations: they require large training datasets due to their reliance on sequence information and

© 2026 The Author(s). Published by Elsevier Inc.
This is an open access article under the CC BY license (http://creativecommons.org/licenses/by/4.0/).

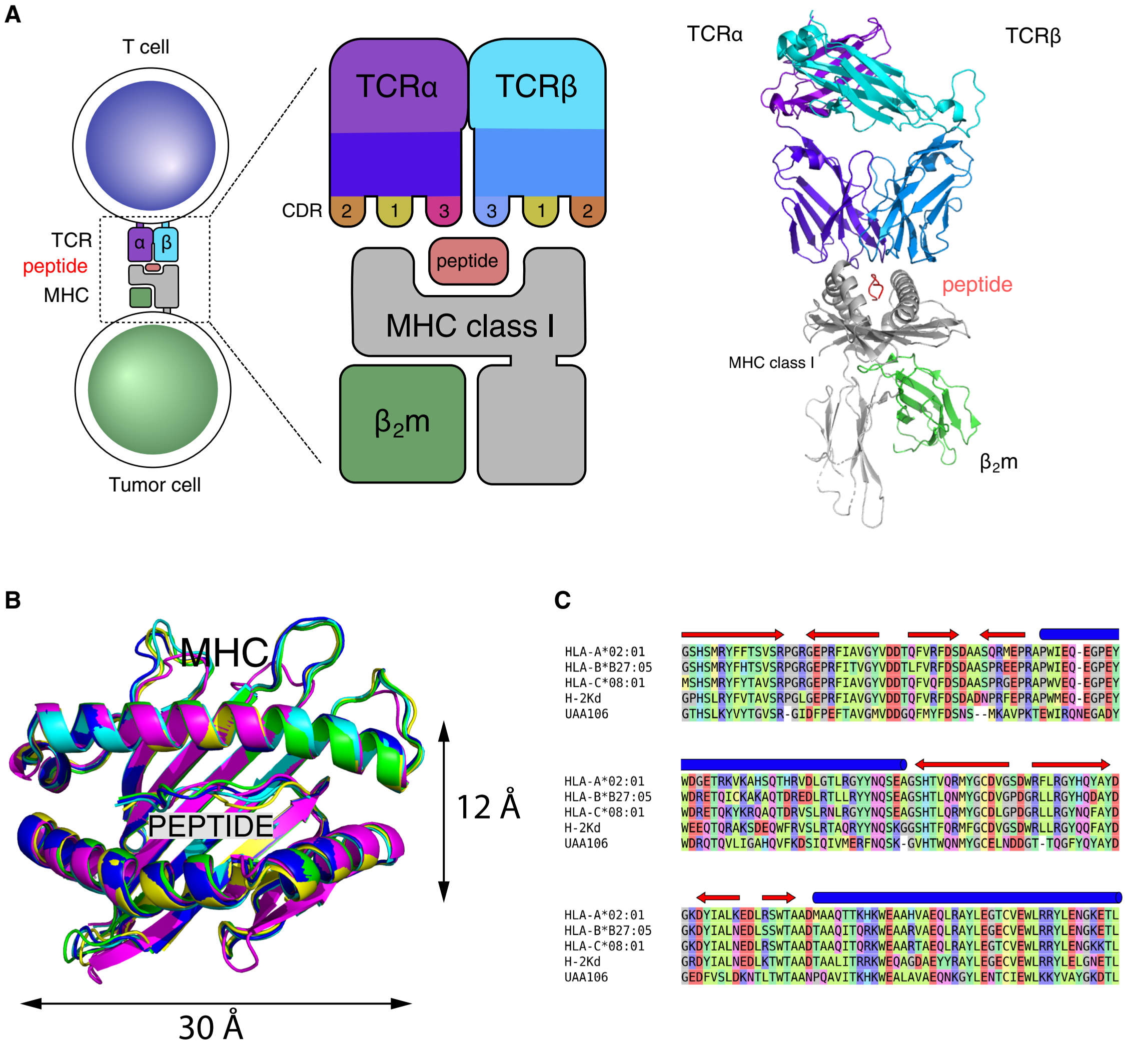

Figure 1. The TCR-peptide-MHC class I (TCR:pMHC-I) complex and neoantigens: their central role in immune surveillance and T cell-mediated immune attacks on tumor cells

(A) TCR nomenclature and the TCR:pMHC-I complex. A TCR has two chains (α and β chains), each having three loops (CDR1, CDR2, and CDR3), where CDR3 plays the primary role in interacting with the peptide. The image on the right shows the structural representation of a TCR:pMHC-I complex based on PDB: 7RTR.

(B) Structural alignment of five different MHC alleles: HLA-A*02:01 (*Homo sapiens*, PDB: 5HHN), HLA-B*27:05 (*Homo sapiens*, PDB: 5IB2), HLA-C*08:01(*Homo sapiens*, PDB: 4NT6), H-2Kd (*Mus musculus*, PDB: 1VGK), and UAA106 (*Ctenopharyngodon idella*, bony fish, PDB: 6LBE). The MHC G-domains all consist of two α-helices and a β-sheet. The peptides are bound in the MHC-binding groove. The root-mean-square deviation (RMSD) values for the structural alignment of these G-domains are all below 0.8 Å, demonstrating highly conserved MHC structures across different alleles and species.

(C) Sequence alignment of MHC G-domains. The sequence alignment of the MHC G-domains from the five alleles shown in (B) demonstrates high diversity in the MHC sequences. The alignment was generated using Clustal Omega[4] and visualized in MRS,[5] with colors representing the charge, hydrophobicity, size, and shape of amino acid side chains. Blue cylinders above the sequences indicate α-helices, while red arrows denote β-strands.

have limited generalizability on unseen alleles,[11] they overlook critical structural features of peptide-MHC (pMHC) interactions, and they struggle to handle peptides of varying lengths. Moreover, they do not provide 3D structural models, which are essential for understanding immunogenicity and guiding TCR design.

Alternatively, 3D structure-based approaches offer several compelling advantages: (1) they naturally handle peptide length variability in 3D space, (2) they are sensitive to mutations in both the spatial and energy landscapes, (3) they are potentially more robust for rare alleles due to the high conservation of MHC structures[11] (Figures 1B and C), and (4) 3D structures provide fundamental insights into the mechanisms of immunotherapies. However, experimental methods like X-ray crystallography, NMR, and cryo-EM are labor intensive and cannot keep up with the diversity of pMHCs. To date, only ~1,000 pMHC structures are available in the Protein DataBank (PDB, www.rcsb.org),[12] in contrast to the high diversity of human leukocyte

antigens (HLAs, over 40,000 variants identified).[13] Therefore, complementary 3D modeling techniques are valuable tools.

Physics-based homology modeling tools such as PANDORA[14] and APE-Gen 2.0[15] can generate multiple MHC-I/II models and provide energy scores indicative of binding affinity (BA). However, they remain computationally expensive (seconds to minutes per case). AlphaFold,[16,17] a powerful deep learning (DL) framework for 3D modeling, also faces limitations: it relies on computationally heavy modules to process multiple sequence alignments (MSAs), which are often unavailable for short peptides lacking evolutionary depth. AlphaFold2-FineTune,[18] a variant adapted for pMHC BA prediction, bypasses MSA searches by using query-to-template alignments but still depends on the costly Evoformer module—a limitation also reported by Mikhaylov et al.[19]

Other methods attempt to address these challenges. MHCfold[20] uses convolutional neural networks to predict 3D pMHC structures and then uses multi-headed attention on the predicted structures to predict BAs. However, it requires additional tools (e.g., MODELLER[21] or SCWRL4[22]) for side-chain reconstruction. Importantly, recent studies[11] have shown that 3D model-based BA predictors have better generalizability than sequence-only methods on unseen alleles (6%–16%), but they typically follow a two-step pipeline: (1) generating 3D pMHC models (seconds to minutes per case) and (2) applying geometric deep learning (GDL) networks for BA estimation. This workflow is time consuming and poorly scalable, particularly for large-scale screening of patient-derived mutations. Thus, there is a clear need for a time-efficient system capable of simultaneously predicting 3D structures and BAs in a single step.

Here, we present SwiftMHC, a transformer that simultaneously predicts pMHC-I BA and generates corresponding 3D structures in milliseconds (Figure 2A). By leveraging the conserved architecture of MHC proteins and avoiding computationally intensive MSA-based modules like AlphaFold's Evoformer, SwiftMHC ensures rapid and accurate predictions. To address the challenge of modeling peptide conformations without evolutionary constraints, we enhance the training dataset with physics-based 3D models and biochemistry-derived BA data, enabling synergistic improvements in both structural and affinity predictions through residue-to-residue attention mechanisms.

With SwiftMHC, we demonstrate the feasibility of designing task-specific AI models that can perform on par with large general-purpose AI systems such as AlphaFold, while being substantially faster. Importantly, SwiftMHC provides both the binding prediction and 3D structural models, crucial for downstream applications such as TCR design. Focusing on HLA-A*02:01 9-mers—given its clinical relevance, prevalence, and the availability of data—this study provides a robust proof-of-concept. This work highlights the promise of building specialized DL systems on physics-derived 3D models, especially in data-scarce domains such as CDR3 loop modeling for TCRs and antibody-antigen interactions, where evolutionary information is absent. Finally, we outline the current limitations of SwiftMHC and potential avenues for future improvement.

Key contributions of this work include the following:

1. Showcasing the power of task-specific small AI models trained on physics-derived synthetic data in a data-scarce domain, achieving remarkable speed and precision over general-purpose big AI systems such as AlphaFold.
2. Establishing SwiftMHC's performance for HLA-A*02:01 9-mer peptides, one of the most prevalent alleles in the human population,[23–25] enabling large-scale screening of patient tumor genomes and expanding the repertoire of candidate target peptides.
3. Overcoming the speed bottleneck of structure-based MHC-peptide AI predictors. While structure-based predictors are shown to have better generalizability than the popular sequence-base approaches, their limited speed has hindered scalability.[11,26]
4. Enabling 3D model prediction for safer therapies. By predicting pMHC 3D structures in milliseconds, SwiftMHC can help identify tumor-specific peptides distinct from self-peptides, paving the way for safer and more precise cancer immunotherapies.

RESULTS

SwiftMHC architecture

The SwiftMHC network utilizes a residue-to-residue attention mechanism to learn to predict BAs and structural features based on interactions between neighboring amino acids (Figure 2A). Specifically, we apply the Self Attention networks on MHC structures and peptide sequences, respectively, to update each residue's representations with its neighborhood information. Then, we apply cross-attention networks to learn the attention weights between MHC and peptide residues, which are expected to encode the interaction information between the peptide and the MHC. This information is used to predict the 3D models and the BAs.

Modeling flexible peptides requires efficient representation of residues and their movement in space. The bond angle between nitrogen (N), alpha carbon (Cα), and carbonyl carbon (C) is relatively fixed due to the constraints imposed by the molecular geometry and electronic structure of these atoms. Following AlphaFold2's approach,[17] we model each residue backbone as a rigid-body triangle with the bond angle N–Cα–C of 109°, allowing free rotation and translation in space while reducing the sampling space (Figure 2B). We use unit quaternions to represent the rotations of a peptide residue as they are more efficient and compact than 3×3 rotation matrices (requiring only 4 parameters vs. 9) and offer better numerical stability. Thus, the movement of each residue backbone is modeled as a local frame, which encodes the rotation (i.e., a unit quaternion represented as a vector of 4×1) and translation (a 3×1 vector) of each residue relative to a global coordinate system (supplemental information, Subsection 1.2.1). Additionally, the peptide bond torsion angle (ω) is typically 180° or 0°, adding rigidity to the backbone (Figure 2C). We take advantage of this with an auxiliary loss on ω, along with other structure losses (supplemental information, Subsection 2.4.3; Figure S4CD). See STAR Methods "Computational Efficiency" for additional design choices improving computational efficiency.

The algorithm is structured into four primary modules (Figure 2A).

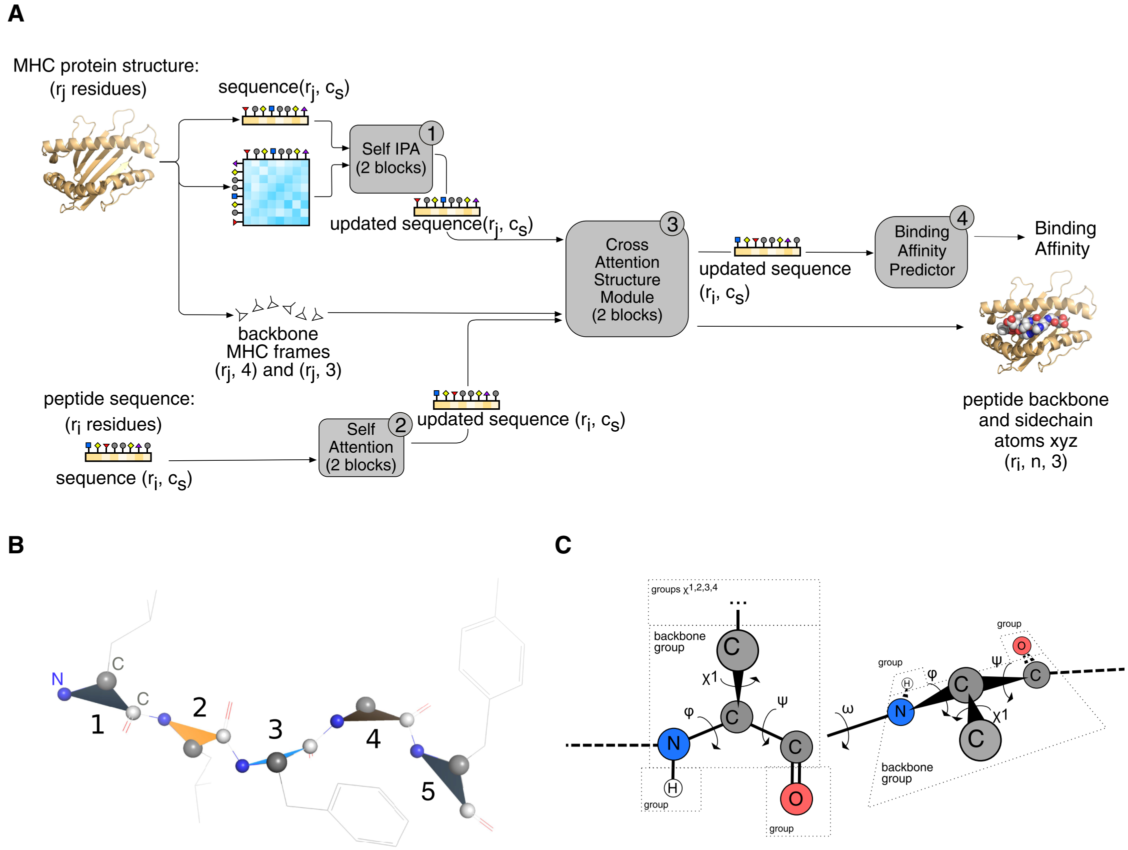

Figure 2. SwiftMHC architecture

(A) Flow diagram of SwiftMHC. The inputs are the structure of the MHC G-domain, consisting of r_j residues and a peptide sequence of r_i residues. The model comprises four key modules: (1) the MHC Self Invariant Point Attention (IPA) module, which processes the MHC structure (for details, see Figure S5); (2) the Peptide Self Attention module, which processes the peptide sequence (Figures S6A and S6B); (3) the Cross-Attention Structure module, which captures interactions between MHC and peptide residues (Figure S7); and (4) the BA Predictor module. Residue distances in the MHC structure are encoded in a proximity matrix, calculated as $1/(1 + d_{ij})$, where d_{ij} is the shortest distance between heavy atoms of residue pairs. The MHC backbone is described using local frames (Figures S4A and S4B). Both MHC and peptide sequences are represented as one-hot encoded tensors with dimensionality c_s, and the structures have maximally n atoms per residue. ($c_s = 32$ and $n = 14$ in this study).

(B) Local frames for a peptide composed of five residues. Backbone local frames are sequentially numbered. Triangles mark the atoms involved in determining the frame orientation (N, Cα, and C).

(C) The torsion angle representation of a peptide with two residues. Different peptide conformations can be derived by rotating torsion angles. Each torsion angle corresponds to a rigid group. Every residue has a backbone rigid group and several side-chain rigid groups The atoms in the rigid group are repositioned as the torsion angle is modified. The backbone torsion angles are φ, ψ, and ω. The ω torsion angles have an empty rigid group, but the rigid groups for φ and ψ both contain one atom, though hydrogens are actually only added in the end and only if OpenMM is used. The side-chain torsion angles are χ_1, χ_2, χ_3, and χ_4. Their corresponding rigid groups contain side-chain atoms.

1. MHC Self Invariant Point Attention (Algorithm 2 in the supplemental information): the purpose of this module is to encode each MHC residue based on its amino acid type and its structural neighbors. This module iteratively applies Self Attention to the MHC structure, drawing inspiration from the AlphaFold2 invariant point attention (IPA) technique to make the residues update each other. It was designed to represent and process interactions between amino acids. For attention weight calculation of each pair of the r_j MHC residues, it utilizes the one-hot-encoded sequence representation of the pairing MHC amino acid types and an $r_j \times r_j \times 1$ proximity matrix that captures their geometric distances. These attention weights are used for updating the MHC residue features; these r_j updated feature vectors are the output of this Self Attention submodule.
2. Peptide Self Attention (Algorithm 3 in the supplemental information): the purpose of this module is to encode each peptide residue based on its amino acid type and its sequence neighbors. It applies Self Attention iteratively (twice, can be configured) to the peptide sequence, allowing the r_i residues to be notified of each other by means of residue-to-residue updating. Relative positional encoding was used to provide residue positions to the attention network. Updating allows residues to know their position in the peptide, allowing the network to distinguish between

them. This peptide Self Attention submodule outputs a sequence of r_i updated peptide residue feature vectors.

3. Cross-Attention Structure (Algorithm 4 in the supplemental information): the purpose of this module is to update each peptide residue based on the interaction between it and the MHC residues. The updated peptide encoding is used to predict peptide structure and used as input of the BA prediction module. This module iteratively performs cross-attention between the r_i peptide residues and r_j MHC residues, employing a ross IPA on the previously updated residue feature vectors and their geometric distances. Throughout these iterations, the module not only updates the peptide features but also refines the peptide's structural representation from an initial starting point at the center of the MHC groove. The MHC features are kept fixed in this module. The first step in this structural refinement process is the prediction of backbone frames (residue positions + orientations), followed by side-chain torsion angle prediction and finally placement of atoms in the cartesian space.
4. BA Prediction (Algorithm 8 in the supplemental information): to allow SwiftMHC to accommodate different peptide lengths, we designed a residue-wise BA predictor. The contribution of each residue to BA was predicted by a multi-layer perceptron (MLP), which processes updated peptide residue features produced by the structural module. The end result of the BA predictor is the sum of the MLP outputs over all peptide residues. This BA value is trained to approach $1 - \log50000(\text{Kd})$ or $1 - \log50000(\text{IC50})$, where K_d and IC_{50} are experimentally determined binding constants.

The SwiftMHC network is trained by a loss function with multiple terms on BA, frame-aligned point error (FAPE), torsion angle, and other structural violations (supplemental information, Subsection 2.6). The BA loss quantifies the difference between the predicted and true BAs between the peptide and the MHC. The FAPE measures the discrepancy in the positions of peptide atoms (both backbone and side chain) between the true and predicted structures. The torsion angle loss assesses differences in torsion angles between the true and predicted structures. Structural violations, which include abnormal bond lengths, bond angles, or atomic clashes, are incorporated only during the fine-tuning phase of training (see STAR Methods, Training).

By default, SwiftMHC predicts BAs and 3D models without running OpenMM refinement. For BA predictions, users may skip writing predicted 3D models to disk to avoid performance slowdowns, referred to as SwiftMHC-BA. Alternatively, enabling short OpenMM refinement produces high-quality models, referred to as SwiftMHC-OpenMM.

SwiftMHC archives SOTA-level BA prediction at sequence-based speeds

In this study, we focused on HLA-A*02:01, one of the most prevalent MHC alleles, observed in 95.7% of Caucasians and 94.3% of Native Americans[23] and extensively studied in clinical trials.[3] Additionally, we focused on 9-mer peptides, the most frequent binding peptide length for MHC-I

We assessed the BA prediction performance of SwiftMHC by using 7,726 BA data points obtained from the Immune Epitope Database (IEDB).[27] To objectively evaluate SwiftMHC's performance on unseen peptides, we clustered the data into ten groups and conducted leave-one-cluster-out cross-validation (see STAR Methods). Additionally, we compared SwiftMHC with four SOTA prediction methods: NetMHCpan 4.1,[10] MHCflurry 2.0,[9] AlphaFold2-FineTune,[18] and MHCfold.[20]

To ensure objective evaluation, MHCflurry 2.0 was retrained on the same datasets as SwiftMHC. We noted that AlphaFold2-FineTune and MHCfold were not retrained and were evaluated using publicly available pretrained models due to computational restraints. As a result, at least some, if not all, of our test cases were included in the training data of these two methods (see STAR Methods and Figure 3C for details about the data overlap), which could lead to inflated performance results.

Our results demonstrate that SwiftMHC reliably predicts MHC-binding peptides, achieving a median AUC of 0.91, outperforming three SOTA methods: AlphaFold2-FineTune, MHCfold, and retrained MHCflurry 2.0 (Figure 3A). Beyond AUC, SwiftMHC also achieves superior results in terms of area under the precision-recall curve (AUPR) and Pearson's correlation (Figures S2A and S2B). For numerical predictions, see Figure S2C.

Compared to NetMHCpan 4.1, SwiftMHC achieved nearly comparable accuracy: NetMHCpan 4.1 attained a median AUC of 0.92, a Pearson's correlation of 0.81, and an AUPR of 0.89, whereas SwiftMHC reached 0.91, 0.80, and 0.88, respectively. Notably, NetMHCpan 4.1 and MHCflurry 2.0's reported performances might have been slightly inflated, as some of its training data overlapped with our test set.

Where SwiftMHC clearly exceled is inference speed and structural modeling. It predicted binding in only 0.009 s per case on one A100 GPU card (when predicted structures are not written to disk), even faster than NetMHCpan 4.1 (0.081 s/case; Figure 3B) and MHCflurry 2.0 (0.020 s/case; Figure 3B). While MHCfold-BA was marginally faster (0.007s) when sidechain modeling was omitted, it exhibited a lower AUC (Figure 3A) and less accurate 3D models (Figure 3G). In contrast, SwiftMHC not only predicted BAs but also generated high-quality 3D pMHC models (see below), a critical feature for downstream TCR design.

SwiftMHC accurately predicted both high- and low-affinity binders. We evaluated SwiftMHC's performance across different BA ranges by analyzing a confusion matrix (Figure S2E). The results show that SwiftMHC predicts most accurately for peptides with high (50–500 nM) and low (5,000–50,000 nM) binding affinities. This indicates that the model does not systematically overpredict binding and is capable of distinguishing non-binders effectively.

SwiftMHC is robust to peptide single-point mutations

To assess SwiftMHC's robustness to single-point mutations, we predicted BAs for 2,838 single amino acid variants derived from the original dataset of 7,726 peptides, using the network models trained on the wild-type cluster's respective folds, and the MHC structure from PDB: 3MRD. For each mutant, we calculated the true change in ΔG (ΔΔG) relative to its wild-type counterpart and

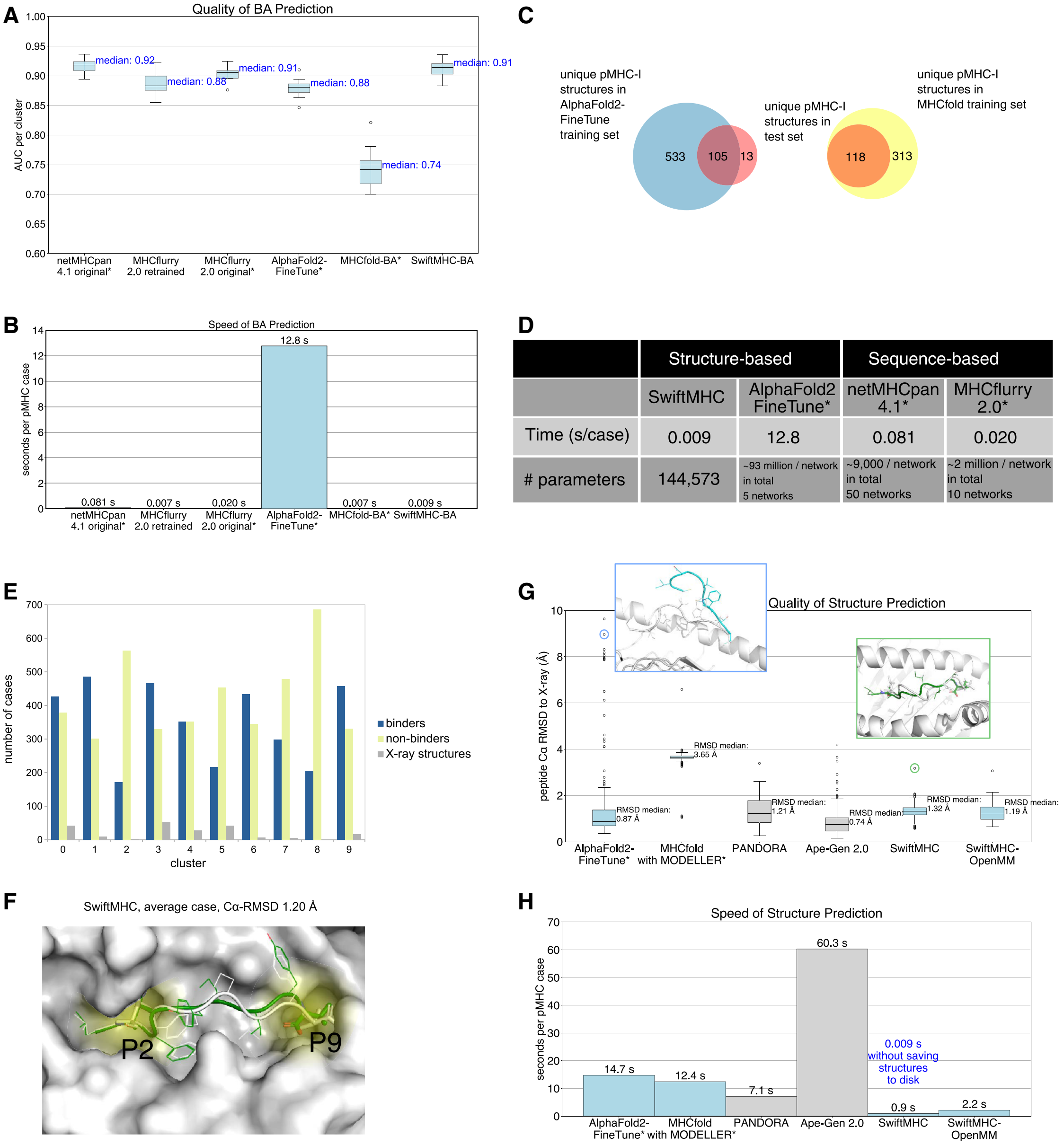

	Structure-based		Sequence-based	
	SwiftMHC	AlphaFold2 FineTune*	netMHCpan 4.1*	MHCflurry 2.0*
Time (s/case)	0.009	12.8	0.081	0.020
# parameters	144,573	~93 million / network in total 5 networks	~9,000 / network in total 50 networks	~2 million / network in total 10 networks

Figure 3. Comparison of BA prediction quality, speed, and structure prediction quality of SwiftMHC and SOTA methods (the asterisks (*) denote the methods that were not retrained and have seen the test data, potentially leading to inflated performance estimates).

(A) BA prediction quality. The boxplots display the distribution of the AUC for each of the 10 HLA-A*02:01 9-mer peptide clusters. A perfect model has an AUC of 1, while a model with no discrimination has an AUC of 0.5. Boxplots show the median (center line), interquatile range (IQR, box; 25th–75th percentiles), and the whiskers extending to 1.5 IQR; points beyond whiskers represent outliers.

(B) BA prediction speed.

(C) The overlap between the unique pMHC-I structures in the training set of AlphaFold2-FineTune and MHCfold and the X-ray structures used as the test set.

(D) Speed and estimated number of trainable parameters for two structure-based and two SOTA sequence-based methods. Note that though the number of parameters in netMHCpan 4.1 is small, no GPU usage was observed.

(E) Data distribution of binders, non-binders, and X-ray structures across clusters. Binders, K_d or IC_{50} < 500 nM; non-binders, K_d or IC_{50} > 500 nM.

(F) Example SwiftMHC-generated structure (PDB ID: 1HHK; Cα-RMSD: 1.20 Å), overlaid with its X-ray structure. P2 and P9 pockets are labeled yellow.

(G) 3D modeling accuracy. Peptide Cα-RMSDs are reported against X-ray structures. Light blue: DL-based methods. Gray: Physics-based methods. SwiftMHC (with and without OpenMM refinement) performs comparably to SOTA methods. The light blue circle points to an outlier by AlphaFold2-FineTune (PDB ID: 3MRG and RMSD; 8.96 Å). The greed circle points to an outlier by SwiftMHC (PDB ID: 2GTW) where different anchors than in the X-ray structure are predicted, achieving the highest tested Cα-RMSD for SwiftMHC (3.04 Å).

(H) Structure prediction speed. The bar plot displays the average modeling speed per case, measured in batch mode for each of the six structure prediction methods. SwiftMHC achieves 0.009 s/case without outputting structures in batch mode (batch size = 64). With file writing and OpenMM, SwiftMHC achieves 0.9–2.2 s/case.

Table 1. Methods evaluated for BA prediction and structure prediction

Methods	Type	Input	3D modeling	Numerical BA prediction	Binary BA classification
SwiftMHC	DL-based	MHC structure, peptide sequence	✔	✔	✔
AlphaFold2 -FineTune[a]	DL-based	pMHC sequence, other pMHC template structures	✔	✔	
MHCfold[a]	DL-based	pMHC sequence	✔	✔	
PANDORA	physics-based	peptide sequence, MHC allele name/sequence	✔		
Ape-Gen 2.0	physics-based	peptide sequence, MHC allele name	✔		
MHCflurry 2.0	DL-based	pMHC sequence		✔	
netMHCpan 4.1	DL-based	peptide sequence, MHC allele name		✔	

[a]These methods were used as provided without retraining on our own defined training sets, thereby risking train-test contamination and potential inflated performances of those methods.

compared it to the predicted ΔΔG (Figure S2F). The overall Pearson's correlation between predicted and true ΔΔG values was relatively low (r = 0.33). Focusing specifically on mutations that do not shift a peptide from the non-binder to binder status ($IC_{50}/K_d < 500$ nM), or vice versa, SwiftMHC incorrectly classified only 11% of either the wild type or the mutant. This indicates that while fine-grained ΔΔG prediction remains challenging, SwiftMHC reliably captures whether a mutation is functionally impactful in terms of binding.

SwiftMHC delivers angstrom-level accuracy in all-atom pMHC structure prediction with high computational efficiency

We evaluated SwiftMHC and four SOTA methods for modeling 3D pMHC-I structures: AlphaFold2-FineTune[18] (DL-based), MHCfold[20] (DL-based), PANDORA 2.0 (physics-based),[14] and Ape-Gen 2.0 (physics-based)[15] (Table 1).

To objectively evaluate the performance, we clustered 202 HLA-A*02:01 9-mer X-ray structures together with the PANDORA models for the 7,726 IEDB entries based on the peptide sequence similarity (Figure 3E). We ensured that the X-ray structures and PANDORA 3D models from the same cluster were not included in the training datasets. PANDORA and Ape-Gen 2.0 rely on homology modeling, using X-ray structures as templates to predict new pMHC-I 3D conformations. To ensure unbiased evaluation, we excluded any X-ray structures containing the same peptide to prevent their use as templates by these methods.

SwiftMHC-OpenMM produced highly accurate pMHC-I 3D models, with a median Cα-RMSD of 1.19 Å (Figure 3G), comparable to three other SOTA methods (median Cα-RMSD: 0.74–1.21 Å), significantly outperforming MHCfold (3.65 Å). Although AlphaFold-FineTune has a lower median Cα-RMSD of 0.87 Å, this advantage likely reflects a substantial overlap between its training and our test data. Importantly, SwiftMHC produced far fewer outliers than AlphaFold-FineTune (Figure 3G), highlighting the benefit of augmenting training with task-specific, physics-derived 3D models.

In terms of computational efficiency, SwiftMHC processed each case in 0.9 s (0.009 s per case when excluding PDB file writing) and 2.2 s with OpenMM energy minimization (Figure 3H). When disk writing was bypassed, SwiftMHC performed up to thousands of times faster than existing SOTA 3D modeling approaches.

Outlier analysis

The peptide-binding groove of HLA-A*02:01 has two primary deep pockets, P2 and P9, which are critical for peptide binding and stabilization.[28] With a few exceptions (e.g., 2GTW, discussed below), the P2 and P9 pockets commonly anchor the 2nd and 9th residues of the peptide. Consistently, most of the 3D models from SwiftMHC have a backbone structure similar to their X-ray counterpart, with common anchor positions 2 and 9 (Figure 3F). The top outlier for SwiftMHC and PANDORA is 2GTW, of which the X-ray structure has anchor positions 1 and 9, but it is predicted with anchor positions 2 and 9 (Figure 3G). Given that PANDORA uses netMHCpan 4.1 for anchor prediction, and SwiftMHC was trained on PANDORA models, SwiftMHC likely inherits these netMHCpan-based prediction errors, reducing accuracy in such cases.

MHCfold exhibited a notably higher median Cα-RMSD (3.65 Å) than the other methods (0.87–1.32 Å). Although AlphaFold2-FineTune achieved a low median Cα-RMSD, it produced many outliers, including at least two 3D models in which one end of the peptides was positioned outside the MHC groove (Figure 3G).

SwiftMHC-generated 3D models closely approximate the quality of X-ray structures

In addition to root-mean-square deviation (RMSD), we assessed model quality by evaluating van der Waals clashes, chirality, and backbone dihedral angles.

Number of van der Waals clashes

The OpenMM energy minimization step in SwiftMHC effectively reduces van der Waals clashes (see definition in STAR Methods), which occur when atoms are positioned too closely. Across the 202 test cases, these reductions are comparable to the levels achieved by the other methods (Figure 4A), highlighting the importance of a final molecular dynamics step to ensure the physical realism of the 3D models.

Chirality

Chirality in amino acids refers to the property where an amino acid molecule is non-superimposable on its mirror

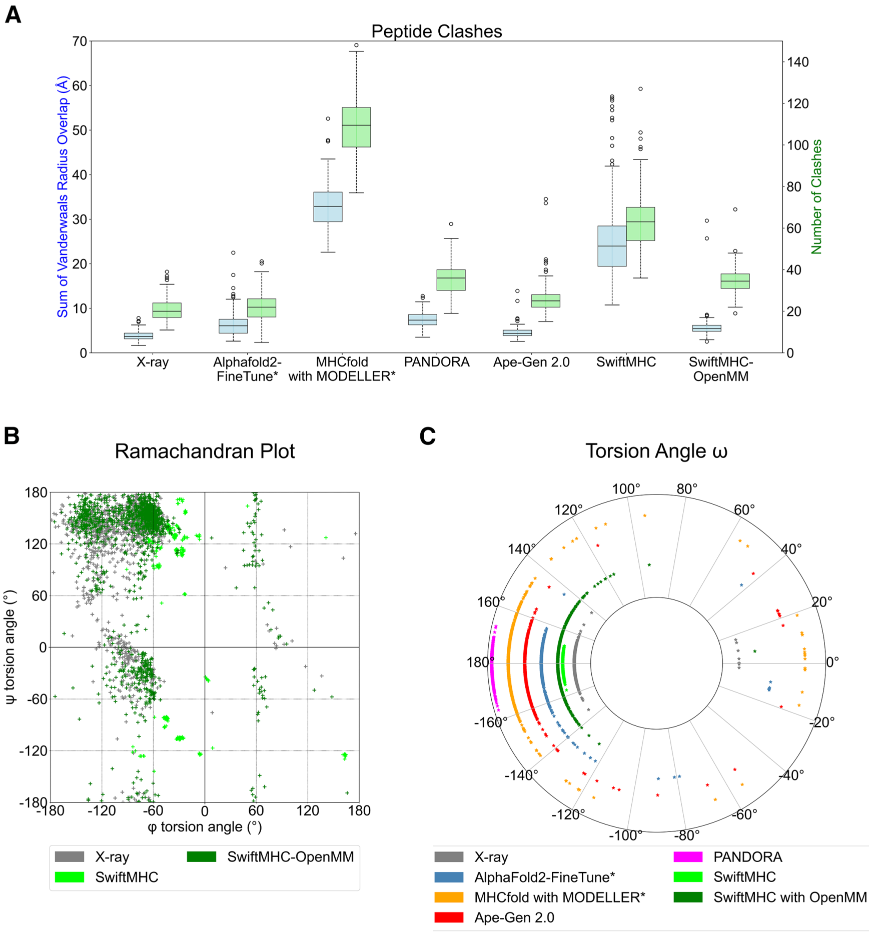

Figure 4. Structural characterizations of SwiftMHC-generated 3D models for the 202 X-ray test cases

(A) Distribution of the number of clashes and the sum of their van der Waals radius overlaps per 3D model/structure. Boxplots show the median, interquartile range (IQR), and whiskers extending to the 1.5 IQR; points beyond whiskers represent outliers.

(B) Ramachandran plot for the distribution of backbone φ and ψ torsion angles in both SwiftMHC 3D models and the X-ray structures.

(C) Distribution of the ω peptide bond torsion angle for all 3D models and X-ray structures.

image: L- and D-enantiomers. Chirality plays a crucial role in the structure and function of biomolecules, as the spatial arrangement of groups affects how amino acids interact with other molecules. Except for glycine, amino acids are chiral molecules and the vast majority of amino acids in proteins and enzymes of living organisms are in the L-form. In the 202 test X-ray cases, some amino acids lacked side-chain atoms, which were restored using OpenMM PDBfixer.[29] However, in some instances, PDBfixer introduced D-form amino acids. Since both SwiftMHC and AlphaFold2-FineTune use these structures as input for model generation, we specifically examined D-form amino acids that were introduced in the models (Table 2). SwiftMHC maintains chirality by rotating and translating local frames of amino acids, as well as placing the chiral hydrogen before executing OpenMM. As a result, it produced L-form amino acids in the generated peptide structures. However, when OpenMM was used for energy minimization, some L-form amino acids were converted to D-form. APE-Gen 2.0, which also relies on OpenMM, exhibited the same issue, and even introduced D-form amino acids in the peptide structure. In contrast, AlphaFold2-FineTune and PANDORA, which do not use OpenMM, did not introduce any D-form amino acids. MHCfold, which predicts individual atomic positions around the chiral Cα atom, introduced a D-form amino acid in one of its models.

Dihedral angles of the peptide backbone

The backbone torsion angles, ϕ (phi) and ψ (psi), define the conformation of a protein's backbone. These angles are constrained by steric clashes, such as repulsions between side-chain atoms or backbone atoms, which limit the range of energetically favorable conformations a protein can adopt. Validating computational 3D protein models includes verifying that the dihedral angles fall within the naturally observed regions of the Ramachandran plot, as deviations can signal errors in the model. To this end, we generated Ramachandran plots for the X-ray structures and the 3D models produced by all six methods (Figures 4B and S1). The φ and ψ torsion angles produced by the DL part of SwiftMHC were restricted to several small areas in the Ramachandran plot (Figure 4B). After energy minimization in OpenMM, these angles were more widespread, and the SwiftMHC distribution became more closely aligned with that of the experimentally determined X-ray structures.

ω torsion angles and cis/trans configurations

"*Trans*" and "*cis*" refer to the geometric configuration around peptide bonds, specifically involving the arrangement of the atoms adjacent to the peptide bond (the amide bond between the carbonyl carbon of one amino acid and the amide nitrogen of the next). The *trans* configuration is the most common configuration found in proteins, as it is generally more stable due to less steric hindrance between the side chains of the amino acids. In the *trans* configuration, the dihedral angle around the peptide bond is approximately 180°.

All five methods generated 3D models in which the ω torsion angles do not correspond to those observed experimentally in the X-ray structures (Figure 4C). For instance, in case of PDB: 5SWQ, all methods predicted an asparagine-glycine peptide bond with an ω angle ranging from 160.0° to 200.0° (*trans*), whereas the X-ray structure showed this angle as −0.6° (*cis*). Similarly, SwiftMHC predicted seven glutamate-proline peptide bonds with ω angles between 160.0° and 200.0° (*trans*), while

Table 2. Methods evaluated for amino acid chirality in the predicted structures

Methods	PDBfixed[a] X-ray structures used as input	OpenMM used	Average number of chiral amino acids per model[b]	Number of D-amino acids introduced in the MHC	Number of D-amino acids introduced in the peptide
MHCfold with MODELLER			174	1	0
AlphaFold2-FineTune	✔		173	0	0
APE-Gen 2.0		✔	263	22	12
PANDORA			263	0	0
SwiftMHC	✔		174	0	0
SwiftMHC-OpenMM	✔	✔	174	9	0

[a]OpenMM PDBfixer[29] was used to restore missing side-chain atoms in some of the 202 X-ray structures, which resulted in the introduction of 11 D-form amino acids.
[b]Some methods model the entire class I MHC protein structure, while others model only the G-domain, leading to differences in the total number of amino acids.

the X-ray structures indicated that these angles fall between -20.0° and 20.0° (*cis*). Notably, OpenMM appears to shift these specific angles outside the *trans* range, approaching 140°. Furthermore, SwiftMHC predicted nearly all ω angles in the *trans* conformation, although the energy minimization step in OpenMM slightly altered these angles in some cases. An extreme case is the isoleucine-proline bond in the SwiftMHC 3D model for 5ENW. This ω angle was almost flipped by OpenMM from -178° (*trans*) to 7° (*cis*).

DISCUSSION

We introduce SwiftMHC, an attention-based neural network for the rapid and accurate identification of MHC-bound peptides and generating 3D structures. SwiftMHC delivers high-resolution all-atom 3D models with a median Cα-RMSD of 1.19 Å, as well as a remarkable efficiency by processing each case in just 0.009 s (AI mode) to 2.2 s (AI + OpenMM mode) in batch mode on a single A100 GPU. SwiftMHC effectively overcomes the speed bottleneck of structure-based BA predictors while maintaining exceptional accuracy in both BA prediction and 3D structure modeling.

The speed and accuracy of SwiftMHC are attributed to two key innovations: (1) removing the computationally demanding MSA attention module, since MHC structures are conserved and peptides lack evolutionary information, and (2) incorporating physics-derived pMHC-I 3D models into training. While the AI industry often focuses on large, general-purpose models like AlphaFold3,[16] these approaches demand vast datasets, extensive compute resources, and high energy consumption, both during training and inference, while remaining difficult to optimize. In contrast, SwiftMHC demonstrates that smaller, task-specific networks—when augmented with extensive physics-based synthetic data—can achieve superior efficiency without sacrificing accuracy. This design highlights a sustainable path forward for domain-focused biomolecular modeling.

In addition, we explored whether cross-attention weights could provide interpretable signals for prediction confidence. By mapping attention scores onto the MHC surface (Figure S3), we observed distinct patterns between correct (1HHK, anchors at positions 2 and 9) and incorrect (2GTW, anchors at positions 1 and 9 but predicted as 2 and 9) anchor assignments. In the "correct" case, the model concentrated attention on the P2 pocket, while in the incorrectly predicted case, such localized focus was absent. This suggests that attention distributions may serve as indicators of anchor misprediction, although systematic validation will be required to establish their utility.

As the fastest pMHC-I structure predictor, SwiftMHC facilitates large-scale peptide screening for vaccine development

A

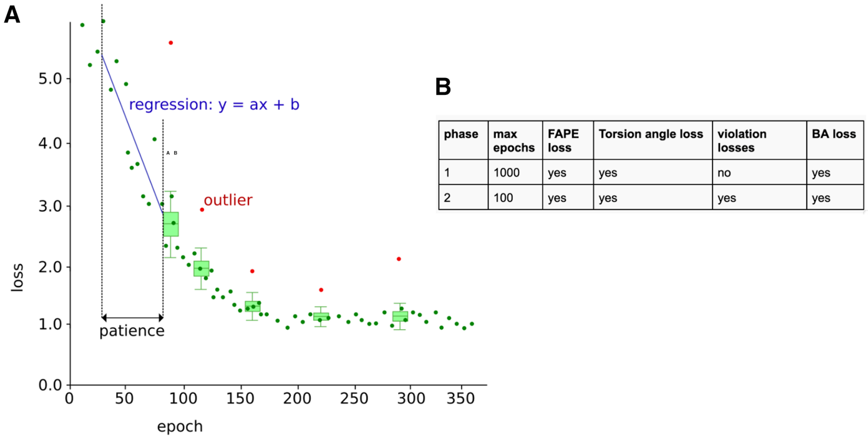

B

phase	max epochs	FAPE loss	Torsion angle loss	violation losses	BA loss
1	1000	yes	yes	no	yes
2	100	yes	yes	yes	yes

Figure 5. Training method

(A) Early stopping criteria. An example scatter loss plot is shown. Dots (dark green) indicate loss values. Boxplots (light green) determine which dots are outliers (red) within the "frame of patience" (50 epochs). The best line (blue) is drawn between the non-outlier values by means of linear regression within the "frame of patience." The parameters of this line are the slope (a) and offset (b).

(B) Parameter settings for each training phase. Columns 3–6 indicate which of the four loss terms are included in the back propagation during each phase (supplementary algorithm 9, lines 29–43). Early stopping is turned on for both phases.

and immunotherapy research. Its high efficiency enhances the ability to identify novel neoantigens from the diverse range of tumor-derived peptide fragments, while providing detailed 3D models for studying molecular interactions and T cell recognition. Furthermore, SwiftMHC's speed offers the potential to construct the first comprehensive 3D self-peptide-HLA (human MHC) library. Such a library could be pivotal for identifying TCR-therapy peptide targets that differ from self-peptides on T cell-exposed surfaces, ultimately contributing to the development of safer and more effective therapeutic strategies.

This proof-of-concept study focuses on HLA-A*02:01 9-mers, selected for their prevalence and clinical relevance. Their abundant data and fixed peptide length make it a relatively straightforward case for sequence-based predictors used in comparison. A key direction for future work is to expand SwiftMHC training set to include additional alleles and peptides of varying lengths. Such expansion is expected to enhance SwiftMHC's generalizability, aligning with the reported 6%–16% performance gains of structure-based methods over sequence-based methods.[11]

Lastly, while optimized for pMHC complexes, our SwiftMHC strategy can be adapted for broader applications, including protein-peptide interactions and CDR3 loop modeling for antibody-antigen and TCR:pMHC-I complexes. The flexibility and the lack of evolutionary signal of peptides and CDR3 loops pose significant challenges for accurate modeling, even for advanced methods like AlphaFold.[30] Capturing peptide and CDR3 conformational changes upon binding is critical for advancing therapeutic TCR and antibody design as well as our understanding of immune recognition and defense.

Limitations of the study

This proof-of-concept study has several limitations. First, SwiftMHC is currently restricted to HLA-A*02:01 9-mer peptides. However, its algorithm is designed to easily incorporate training sets for additional alleles and peptides of varying lengths.

Second, for computational efficiency, we chose to design SwiftMHC to use fixed MHC orientations, not allowing random orientation options. This limitation could be addressed by redesigning the network to incorporate rotation-translation equivariance, such as using backbone frames based on the MHC protein's intrinsic orientation rather than identity frames.

Third, SwiftMHC does not yet account for peptides with post-translational modifications (PTMs) such as phosphorylation, glycosylation, methylation, and acetylation. Addressing this would require training on datasets with sufficient examples of post-translationally modified structures. While SwiftMHC cannot currently model PTMs directly, it can generate reasonable starting conformations for downstream molecular dynamics (MD) simulations of modified peptides.

Finally, the speed evaluation of SwiftMHC in this study was conducted using a maximum batch size of 64 pMHC cases, optimized for large datasets (~8,000 cases, as in the BA test datasets), which efficiently filled most batches. However, SwiftMHC's efficiency may decline when processing very small datasets with only a handful of cases per batch. Additionally, the impact of software initialization time, which may reduce efficiency for smaller datasets, was not explored. Further investigation into time consumption for larger datasets could offer additional insights into its performance efficiency.

RESOURCE AVAILABILITY

Lead contact

Requests for further information and resources should be directed to and will be fulfilled by the lead contact, Dr. Li C. Xue (li.xue@radboudumc.nl).

Materials availability

This study did not generate new unique reagents.

Data and code availability

- Data: All input data (including PANDORA 3D models, BA data, preprocessed datasets), network models, and results (including torsion angles, clash counts, chiralities, and RMSD and BA predictions) have been deposited at Zenodo and are accessible at https://doi.org/10.5281/zenodo.14968655. This includes utility scripts that have been used to process the data.
- Code: All code for data preprocessing, evaluation, and prediction is available at https://doi.org/10.5281/zenodo.18001720
- Additional information: Any additional information required to reanalyze the data reported in this paper is available from the lead contact upon request.

ACKNOWLEDGMENTS

This project was supported by the Hanarth Fonds 2022 (NL), the Kika foundation (grant no. 454, NL), NWO-XS (OCENW.XS23.2.130, NL), and the NLeSc grant for the 3D-Vac project (grant ID: NLESC.OEC.2021.008). The computation was partly supported by the NVIDIA Academic Grant Program. We thank SurfSara in the Netherlands for their generous GPU and CPU computing resources (grant numbers EINF2380, EINF-10427, and EINF-11930). We also thank Dr. Dario Marzella for providing the dataset and helpful discussions.

AUTHOR CONTRIBUTIONS

C.A.B.B. contributed to the design and development of software, experimentation, and writing of the manuscript; G.C. performed manuscript review and editing; C.G. performed manuscript review and code optimization; D.T.R. reviewed and edited the manuscript and participated in consultation on DL-related questions; D.F. reviewed and edited the manuscript and tested the code; Y.J.MA. performed manuscript review and editing and examined possible chirality problems in AlphaFold3-generated models; L.C.X. designed and supervised the project, in addition to reviewing and editing of the manuscript. All authors have reviewed and edited this manuscript.

DECLARATION OF INTERESTS

The authors declare no conflict of interest.

DECLARATION OF GENERATIVE AI AND AI-ASSISTED TECHNOLOGIES IN THE WRITING PROCESS

During the preparation of this work, the author(s) used ChatGPT (https://chatgpt.com) in order to improve the writing style. After using this tool, the authors reviewed and edited the content as needed and take full responsibility for the content of the publication.

STAR★METHODS

Detailed methods are provided in the online version of this paper and include the following:

- KEY RESOURCES TABLE
- METHOD DETAILS
  - Data composition

- Data preprocessing
- Software and libraries used
- Training
- Post processing the resulting structures
- SOTA methods
- QUANTIFICATION AND STATISTICAL ANALYSIS
  - Computational efficiency
  - The training data overlap between AlphaFold2-FineTune, MHCfold and our test data
  - Evaluation of BA prediction
  - Evaluation of structure prediction
- ADDITIONAL RESOURCES

SUPPLEMENTAL INFORMATION

Supplemental information can be found online at https://doi.org/10.1016/j.crmeth.2026.101364.

Received: February 28, 2025
Revised: July 1, 2025
Accepted: February 19, 2026
Published: March 31, 2026

REFERENCES

1. Bagchi, S., Yuan, R., and Engleman, E.G. (2021). Immune Checkpoint Inhibitors for the Treatment of Cancer: Clinical Impact and Mechanisms of Response and Resistance. Annu. Rev. Pathol. *16*, 223–249. https://doi.org/10.1146/annurev-pathol-042020-042741.
2. Johnson, D.B., Nebhan, C.A., Moslehi, J.J., and Balko, J.M. (2022). Immune-checkpoint inhibitors: long-term implications of toxicity. Nat. Rev. Clin. Oncol. *19*, 254–267. https://doi.org/10.1038/s41571-022-00600-w.
3. Baulu, E., Gardet, C., Chuvin, N., and Depil, S. (2023). TCR-engineered T cell therapy in solid tumors: State of the art and perspectives. Sci. Adv. *9*, eadf3700. https://doi.org/10.1126/sciadv.adf3700.
4. Sievers, F., and Higgins, D.G. (2014). Clustal Omega. Curr. Protoc. Bioinforma. *48*, 3.13.1–3.13.16. https://doi.org/10.1002/0471250953.bi0313s48.
5. Hekkelman, M.L., and Vriend, G. (2005). MRS: a fast and compact retrieval system for biological data. Nucleic Acids Res. *33*, W766–W769. https://doi.org/10.1093/nar/gki422.
6. Nielsen, M., Andreatta, M., Peters, B., and Buus, S. (2020). Immunoinformatics: Predicting Peptide–MHC Binding. Annu. Rev. Biomed. Data Sci. *3*, 191–215. https://doi.org/10.1146/annurev-biodatasci-021920-100259.
7. Antunes, D.A., Abella, J.R., Devaurs, D., Rigo, M.M., and Kavraki, L.E. (2018). Structure-based Methods for Binding Mode and Binding Affinity Prediction for Peptide-MHC Complexes. Curr. Top. Med. Chem. *18*, 2239–2255. https://doi.org/10.2174/1568026619666181224101744.
8. Lin, M.J., Svensson-Arvelund, J., Lubitz, G.S., Marabelle, A., Melero, I., Brown, B.D., and Brody, J.D. (2022). Cancer vaccines: the next immunotherapy frontier. Nat. Cancer *3*, 911–926. https://doi.org/10.1038/s43018-022-00418-6.
9. O'Donnell, T.J., Rubinsteyn, A., and Laserson, U. (2020). MHCflurry 2.0: Improved Pan-Allele Prediction of MHC Class I-Presented Peptides by Incorporating Antigen Processing. Cell Syst. *11*, 42–48.e7. https://doi.org/10.1016/j.cels.2020.06.010.
10. Reynisson, B., Alvarez, B., Paul, S., Peters, B., and Nielsen, M. (2020). NetMHCpan-4.1 and NetMHCIIpan-4.0: improved predictions of MHC antigen presentation by concurrent motif deconvolution and integration of MS MHC eluted ligand data. Nucleic Acids Res. *48*, W449–W454. https://doi.org/10.1093/nar/gkaa379.
11. Marzella, D.F., Crocioni, G., Radusinović, T., Lepikhov, D., Severin, H., Bodor, D.L., Rademaker, D.T., Lin, C., Georgievska, S., Renaud, N., et al. (2024). Geometric deep learning improves generalizability of MHC-bound peptide predictions. Commun. Biol *7*, 1661. https://doi.org/10.1038/s42003-024-07292-1.
12. Burley, S.K., Bhikadiya, C., Bi, C., Bittrich, S., Chen, L., Crichlow, G.V., Duarte, J.M., Dutta, S., Fayazi, M., Feng, Z., et al. (2022). RCSB Protein Data Bank: Celebrating 50 years of the PDB with new tools for understanding and visualizing biological macromolecules in 3D. Protein Sci. *31*, 187–208. https://doi.org/10.1002/pro.4213.
13. Barker, D.J., Maccari, G., Georgiou, X., Cooper, M.A., Flicek, P., Robinson, J., and Marsh, S.G.E. (2023). The IPD-IMGT/HLA Database. Nucleic Acids Res. *51*, D1053–D1060. https://doi.org/10.1093/nar/gkac1011.
14. Parizi, F.M., Marzella, D.F., Ramakrishnan, G., T Hoen, P.A.C., Karimi-Jafari, M.H., and Xue, L.C. (2023). PANDORA v2.0: Benchmarking peptide-MHC II models and software improvements. Front. Immunol. *14*, 1285899. https://doi.org/10.3389/fimmu.2023.1285899.
15. Fasoulis, R., Rigo, M.M., Lizée, G., Antunes, D.A., and Kavraki, L.E. (2024). APE-Gen2.0: Expanding Rapid Class I Peptide–Major Histocompatibility Complex Modeling to Post-Translational Modifications and Noncanonical Peptide Geometries. J. Chem. Inf. Model. *64*, 1730–1750. https://doi.org/10.1021/acs.jcim.3c01667.
16. Abramson, J., Adler, J., Dunger, J., Evans, R., Green, T., Pritzel, A., Ronneberger, O., Willmore, L., Ballard, A.J., Bambrick, J., et al. (2024). Accurate structure prediction of biomolecular interactions with AlphaFold 3. Nature *630*, 493–500. https://doi.org/10.1038/s41586-024-07487-w.
17. Jumper, J., Evans, R., Pritzel, A., Green, T., Figurnov, M., Ronneberger, O., Tunyasuvunakool, K., Bates, R., Žídek, A., and Potapenko, A. (2021). Highly accurate protein structure prediction with AlphaFold. Nature *596*, 583–589. https://doi.org/10.1038/s41586-021-03819-2.
18. Motmaen, A., Dauparas, J., Baek, M., Abedi, M.H., Baker, D., and Bradley, P. (2023). Peptide-binding specificity prediction using fine-tuned protein structure prediction networks. Proc. Natl. Acad. Sci. *120*, e2216697120. https://doi.org/10.1073/pnas.2216697120.
19. Mikhaylov, V., Brambley, C.A., Keller, G.L.J., Arbuiso, A.G., Weiss, L.I., Baker, B.M., and Levine, A.J. (2024). Accurate modeling of peptide-MHC structures with AlphaFold. Structure *32*, 228–241.e4. https://doi.org/10.1016/j.str.2023.11.011.
20. Aronson, A., Hochner, T., Cohen, T., and Schneidman-Duhovny, D. (2022). Structure modeling and specificity of peptide-MHC class I interactions using geometric deep learning. Preprint at bioRxiv. https://doi.org/10.1101/2022.12.15.520566.
21. Webb, B., and Sali, A. (2017). Protein structure modeling with MODELLER. In Functional genomics: Methods and protocols (New York, NY: Springer New York), pp. 39–54.
22. Krivov, G.G., Shapovalov, M.V., and Dunbrack, R.L. (2009). Improved prediction of protein side-chain conformations with SCWRL4. Proteins *77*, 778–795. https://doi.org/10.1002/prot.22488.
23. Ellis, J.M., Henson, V., Slack, R., Ng, J., Hartzman, R.J., and Katovich Hurley, C. (2000). Frequencies of HLA-A2 alleles in five U.S. population groups. Hum. Immunol. *61*, 334–340. https://doi.org/10.1016/S0198-8859(99)00155-X.
24. Cao, K., Hollenbach, J., Shi, X., Shi, W., Chopek, M., and Fernández-Viña, M.A. (2001). Analysis of the frequencies of HLA-A, B, and C alleles and haplotypes in the five major ethnic groups of the United States reveals high levels of diversity in these loci and contrasting distribution patterns in these populations. Hum. Immunol. *62*, 1009–1030. https://doi.org/10.1016/S0198-8859(01)00298-1.
25. Ayed, K., Ayed-Jendoubi, S., Sfar, I., Labonne, M.-P., and Gebuhrer, L. (2004). HLA class-I and HLA class-II phenotypic, gene and haplotypic frequencies in Tunisians by using molecular typing data. Tissue Antigens *64*, 520–532. https://doi.org/10.1111/j.1399-0039.2004.00313.x.
26. Moon, S., Zhung, W., and Kim, W.Y. (2024). Toward generalizable structure-based deep learning models for protein–ligand interaction

prediction: Challenges and strategies. WIREs Comput. Mol. Sci. *14*, e1705. https://doi.org/10.1002/wcms.1705.

27. Vita, R., Mahajan, S., Overton, J.A., Dhanda, S.K., Martini, S., Cantrell, J.R., Wheeler, D.K., Sette, A., and Peters, B. (2019). The Immune Epitope Database (IEDB): 2018 update. Nucleic Acids Res. *47*, D339–D343. https://doi.org/10.1093/nar/gky1006.
28. Zhang, C., Anderson, A., and DeLisi, C. (1998). Structural principles that govern the peptide-binding motifs of class I MHC molecules. J. Mol. Biol. *281*, 929–947. https://doi.org/10.1006/jmbi.1998.1982.
29. Eastman, P., Swails, J., Chodera, J.D., McGibbon, R.T., Zhao, Y., Beauchamp, K.A., Wang, L.-P., Simmonett, A.C., Harrigan, M.P., Stern, C.D., et al. (2017). OpenMM 7: Rapid development of high performance algorithms for molecular dynamics. PLoS Comput. Biol. *13*, e1005659. https://doi.org/10.1371/journal.pcbi.1005659.
30. Yin, R., and Pierce, B.G. (2024). Evaluation of AlphaFold antibody–antigen modeling with implications for improving predictive accuracy. Protein Sci. *33*, e4865. https://doi.org/10.1002/pro.4865.
31. Martin, A.C.R., and Craig, T.P. (2009). ProFit. Version 3.1 (University College London; Prof Andrew C. R. Martin's group).
32. Zhang, Y., and Skolnick, J. (2005). TM-align: a protein structure alignment algorithm based on the TM-score. Nucleic acids research *33*, 2302–2309.
33. DeLano, W.L. (2002). Pymol: An open-source molecular graphics tool*40.1* (CCP4 Newsl. protein crystallogr), pp. 82–92.
34. Andreatta, M., Alvarez, B., and Nielsen, M. (2017). GibbsCluster: unsupervised clustering and alignment of peptide sequences. Nucleic Acids Res. *45*, W458–W463. https://doi.org/10.1093/nar/gkx248.
35. Imambi, S., Prakash, K.B., and Kanagachidambaresan, G.R. (2021). PyTorch. In Programming with TensorFlow EAI/Springer Innovations in Communication and Computing, K.B. Prakash and G.R. Kanagachidambaresan, eds. (Springer International Publishing), pp. 87–104. https://doi.org/10.1007/978-3-030-57077-4_10.
36. Ahdritz, G., Bouatta, N., Floristean, C., Kadyan, S., Xia, Q., Gerecke, W., O'Donnell, T.J., Berenberg, D., Fisk, I., Zanichelli, N., et al. (2022). OpenFold: Retraining AlphaFold2 yields new insights into its learning mechanisms and capacity for generalization. Preprint at bioRxiv. https://doi.org/10.1101/2022.11.20.517210.
37. Chapman, B., and Chang, J. (2000). Biopython: Python tools for computational biology. SIGBIO Newsl. *20*, 15–19. https://doi.org/10.1145/360262.360268.
38. Chys, P., and Chacón, P. (2013). Random Coordinate Descent with Spinor-matrices and Geometric Filters for Efficient Loop Closure. J. Chem. Theory Comput. *9*, 1821–1829. https://doi.org/10.1021/ct300977f.
39. Koes, D.R., Baumgartner, M.P., and Camacho, C.J. (2013). Lessons Learned in Empirical Scoring with smina from the CSAR 2011 Benchmarking Exercise. J. Chem. Inf. Model. *53*, 1893–1904. https://doi.org/10.1021/ci300604z.

STAR★METHODS

KEY RESOURCES TABLE

REAGENT or RESOURCE	SOURCE	IDENTIFIER
Deposited data		
7,726 BA data entries, originating from IEDB with cluster numbers added.<br>In archive: input_data/IEDB-BA-with-clusters.csv	Zenodo	https://doi.org/10.5281/zenodo.14968656
202 X-ray structures originating from PDB, chain identifiers modified, missing atoms added, cluster numbers added.<br>In archive:<br>input_data/PDB-xray-clusters.csv<br>input_data/xray-pdbfixed/	Zenodo	https://doi.org/10.5281/zenodo.14968656
Results of structural analysis of 202 X-ray structures, originating from PDB, chain identifiers modified, missing atoms added, cluster numbers added.<br>In archive:<br>input-data/xray-pdbfixed/clashes.csv<br>input-data/xray-pdbfixed/ramachandran.csv<br>input-data/xray-pdbfixed/omegas.csv<br>input-data/xray-pdbfixed/chirality.csv	Zenodo	https://doi.org/10.5281/zenodo.14968656
Results of comparing all 202 X-ray structures against each other in terms of structural variation.<br>In archive: input-data/xray-pdbfixed/TMalign-all-vs-all-rmsd.csv	Zenodo	https://doi.org/10.5281/zenodo.14968656
The result from Gibbs clustering the 7,726 BA data entries from IEDB into ten clusters.<br>In archive: input_data/cluster-data/	Zenodo	https://doi.org/10.5281/zenodo.14968656
The results from checking the overlap between the 202 X-ray structures in the test set and the training data from AlphaFold2-FineTune and MHCfold.<br>In archive: input-data/overlap/	Zenodo	https://doi.org/10.5281/zenodo.14968656
2,838 single point mutants, found by comparing 7,726 BA data entries from IEDB.<br>In archive: input_data/mutants/	Zenodo	https://doi.org/10.5281/zenodo.14968656
SwiftMHC masks and reference structure, used for preprocessing.<br>In archive:<br>input-data/swiftmhc/HLA-A0201-CROSS.mask<br>input-data/swiftmhc/HLA-A0201-GDOMAIN.mask<br>input-data/swiftmhc/reference-3MRD.pdb	Zenodo	https://doi.org/10.5281/zenodo.14968656
7,726 PANDORA models used for training the 10 SwiftMHC models.<br>In archive: input-data/swiftmhc/<br>pandora-models-for-training-swiftmhc/	Zenodo	https://doi.org/10.5281/zenodo.14968656
7,726 BA data entries, originating from IEDB split by 10 folds (train, validation, test) according to 10 clusters. This was the input data for MHCflurry 2.0 10-fold cross validation.<br>In archive:<br>input-data/mhcflurry/train-fold?csv<br>input-data/mhcflurry/valid-fold?csv<br>input-data/mhcflurry/BA-cluster?csv (test sets)	Zenodo	https://doi.org/10.5281/zenodo.14968656

(*Continued on next page*)

Continued

REAGENT or RESOURCE	SOURCE	IDENTIFIER
MHCflurry 2.0 retraining hyperparameters<br>In archive: input-data/mhcflurry/mhcflurry-hyperparameters.yaml	Zenodo	https://doi.org/10.5281/zenodo.14968656
7,726 BA data entries, originating from IEDB, converted to fasta format, representing the peptide sequences. This was the input to netMHCpan 4.1 for evaluating BA prediction quality.<br>In archive: input-data/netmhcpan/IEDB-BA.fasta	Zenodo	https://doi.org/10.5281/zenodo.14968656
7,726 BA data entries, originating from IEDB, in fasta format. This is the input data to MHCfold for evaluating BA prediction quality.<br>In archive: input-data/mhcfold/ba.fa	Zenodo	https://doi.org/10.5281/zenodo.14968656
202 X-ray structures, originating from PDB, in fasta format. This is the input data to MHCfold for evaluating structure prediction quality.<br>In archive: input-data/mhcfold/X-ray.fa	Zenodo	https://doi.org/10.5281/zenodo.14968656
6,057 pMHC-I complexes, preprocessed in a format for AlphaFold2-FineTune to work with, representing 7,726 BA data entries from IEDB.<br>In archive:<br>input_data/alphafold2-finetune/alignments-BA/<br>input_data/alphafold2-finetune/targets-BA.tsv)	Zenodo	https://doi.org/10.5281/zenodo.14968656
202 pMHC-I complexes preprocessed in a format for AlphaFold2-FineTune to work with, representing 202 X-ray structures from PDB.<br>In archive:<br>alphafold2-finetune/alignments-xray/<br>alphafold2-finetune/targets-xray.tsv	Zenodo	https://doi.org/10.5281/zenodo.14968656
10 SwiftMHC trained network models, one for each fold. The networks were trained with both structural and BA loss.<br>In archive: network-models/swiftmhc/	Zenodo	https://doi.org/10.5281/zenodo.14968656
10 SwiftMHC trained network models, one for each fold. The networks were trained with only BA loss.<br>In archive: network-models/swiftmhc/	Zenodo	https://doi.org/10.5281/zenodo.14968656
10 SwiftMHC trained network models, one for each fold. The networks were trained with only structural loss.<br>In archive: network-models/swiftmhc/	Zenodo	https://doi.org/10.5281/zenodo.14968656
10 MHCflurry 2.0 trained network model assemblies, one for each fold. The networks were first trained on the training set and then a model was selected on the validation set.<br>In archive:<br>network-models/mhcflurry-retrained/train-fold?<br>network-models/mhcflurry-retrained/valid-fold?	Zenodo	https://doi.org/10.5281/zenodo.14968656
Preprocessed data for SwiftMHC (train, validation, test) for 10 folds. The file format is HDF5.<br>In archive: preprocessed/	Zenodo	https://doi.org/10.5281/zenodo.14968656
Timing results for SwiftMHC evaluations (BA, structure with/without OpenMM).<br>In archive:<br>output-data/swiftmhc/model-ba-cluster?-timings.txt<br>output-data/swiftmhc/model-xray-cluster?-with-openmm-timings.txt<br>output-data/swiftmhc/model-xray-cluster?-without-openmm-timings.txt	Zenodo	https://doi.org/10.5281/zenodo.14968656

(*Continued on next page*)

Continued

REAGENT or RESOURCE	SOURCE	IDENTIFIER
Predicted structures by SwiftMHC, with/without OpenMM.<br>In archive:<br>output-data/swiftmhc/3d-models-with-openmm/<br>output-data/swiftmhc/3d-models-without-openmm/	Zenodo	https://doi.org/10.5281/zenodo.14968656
BA prediction results for SwiftMHC evaluations.<br>In archive: output-data/swiftmhc/predict-ba-cluster?csv	Zenodo	https://doi.org/10.5281/zenodo.14968656
Single point mutant results for evaluation by SwiftMHC.<br>In archive: output-data/swiftmhc/mutants/results-wt-cluster?-evaluated-on-3MRD.csv	Zenodo	https://doi.org/10.5281/zenodo.14968656
Attention weights output by SwiftMHC models for peptides of 1HHK and 2GTW.<br>In archive: output-data/swiftmhc/attention-weights/	Zenodo	https://doi.org/10.5281/zenodo.14968656
Results of comparing SwiftMHC predicted structures with their X-ray counterparts.<br>In archive:<br>output-data/swiftmhc/CA-rmsd-without-openmm.csv<br>output-data/swiftmhc/CA-rmsd-with-openmm.csv	Zenodo	https://doi.org/10.5281/zenodo.14968656
Results of analysing SwiftMHC predicted structures.<br>In archive:<br>output-data/swiftmhc/clashes-with-openmm.csv<br>output-data/swiftmhc/clashes-without-openmm.csv<br>output-data/swiftmhc/chirality-with-openmm.csv<br>output-data/swiftmhc/chirality-without-openmm.csv<br>output-data/swiftmhc/omegas-with-openmm.csv<br>output-data/swiftmhc/omegas-without-openmm.csv<br>output-data/swiftmhc/ramachandran-with-openmm.csv<br>output-data/swiftmhc/ramachandran-without-openmm.csv	Zenodo	https://doi.org/10.5281/zenodo.14968656
SwiftMHC predicted structures from network models trained without BA loss.<br>In archive: output-data/swiftmhc/3d-models-with-openmm/	Zenodo	https://doi.org/10.5281/zenodo.14968656
Results of comparing SwiftMHC predicted structures to their X-ray counterparts. The structures were predicted by network models trained without BA loss.<br>In archive: output-data/swiftmhc/CA-rmsd-with-openmm.csv	Zenodo	https://doi.org/10.5281/zenodo.14968656
Results from SwiftMHC BA prediction, from network models trained without structural loss.<br>In archive: output-data/swiftmhc/BA-cluster?csv	Zenodo	https://doi.org/10.5281/zenodo.14968656
netMHCpan 4.1 timing results from processing 7,726 pMHC-I cases.<br>In archive: output-data/netMHCpan/IEDB-BA-time.txt	Zenodo	https://doi.org/10.5281/zenodo.14968656
netMHCpan 4.1 BA prediction results from processing 7,726 pMHC-I cases.<br>In archive:<br>output-data/netMHCpan/IEDB-BA-results.tsv<br>output-data/netMHCpan/IEDB-BA-results.txt	Zenodo	https://doi.org/10.5281/zenodo.14968656

(*Continued on next page*)

Continued

REAGENT or RESOURCE	SOURCE	IDENTIFIER
Timings for MHCflurry 2.0 cross-validation network models to evaluate each of 10 clusters from 7,726 IEDB BA data entries.<br>In archive: output-data/mhcflurry-crossvalidation/time_cluster?txt	Zenodo	https://doi.org/10.5281/zenodo.14968656
BA predictions for MHCflurry 2.0 cross-validation network model for each of 10 clusters from 7,726 IEDB BA data entries.<br>In archive: output-data/mhcflurry-crossvalidation/result-cluster?csv	Zenodo	https://doi.org/10.5281/zenodo.14968656
Timings for MHCflurry 2.0 (original training) for each of 10 clusters from 7,726 IEDB BA data entries.<br>In archive: output-data/mhcflurry-original/time_cluster?txt	Zenodo	https://doi.org/10.5281/zenodo.14968656
BA predictions for MHCflurry 2.0 (original training) for each of 10 clusters from 7,726 IEDB BA data entries.<br>In archive: output-data/mhcflurry-original/result_cluster?csv	Zenodo	https://doi.org/10.5281/zenodo.14968656
Results of comparing the BA predictions by MHCflurry 2.0 (original training) to the BA predictions of SwiftMHC.<br>In archive:<br>output-data/ba-true-vs-swiftmhc-vs-mhcflurry.csv	Zenodo	https://doi.org/10.5281/zenodo.14968656
PANDORA models with best energy scores, predicting the structures of 202 pMHC-I cases, originating from PDB.<br>In archive: output-data/pandora/best/	Zenodo	https://doi.org/10.5281/zenodo.14968656
Results of comparing 202 best PANDORA models to their PDB X-ray counterpart.<br>In archive:<br>output-data/pandora/best-models-CA-rmsds.csv	Zenodo	https://doi.org/10.5281/zenodo.14968656
Results of structural evaluation of 202 best PANDORA models.<br>In archive:<br>output-data/pandora/best-models-clashes.csv<br>output-data/pandora/best-models-omegas.csv<br>output-data/pandora/best-models-ramachandran.csv<br>output-data/pandora/best-models-chirality.csv	Zenodo	https://doi.org/10.5281/zenodo.14968656
Timing result for PANDORA to process 202 cases in 32 threads.<br>In archive: output-data/pandora/timings.txt	Zenodo	https://doi.org/10.5281/zenodo.14968656
APE-Gen 2.0 models with best energy scores, predicting the structures of 202 pMHC-I cases, originating from PDB.<br>In archive: output-data/apegen2/best/	Zenodo	https://doi.org/10.5281/zenodo.14968656
APE-Gen 2.0 timings for processing each of 202 pMHC-I cases.<br>In archive: output-data/apegen2/????-time.txt	Zenodo	https://doi.org/10.5281/zenodo.14968656
Results of comparing 202 APE-Gen 2.0 models to their X-ray counterparts.<br>In archive:<br>output-data/apegen2/best-models-CA-rmsds.csv	Zenodo	https://doi.org/10.5281/zenodo.14968656

(*Continued on next page*)

CellPress
OPEN ACCESS

Continued

REAGENT or RESOURCE	SOURCE	IDENTIFIER
Results of structural evaluation of 202 best APE-Gen 2.0 models.<br>In archive:<br>output-data/apegen2/best-models-clashes.csv<br>output-data/apegen2/best-models-ramachandran.csv<br>output-data/apegen2/best-models-omegas.csv<br>output-data/apegen2/best-models-chirality.csv	Zenodo	https://doi.org/10.5281/zenodo.14968656
Results of MHCfold structural prediction of 202 pMHC-I cases, corresponding to X-ray structures from the PDB.<br>In archive: output-data/mhcfold/results-xray/????_mhcfold_full_relaxed.pdb	Zenodo	https://doi.org/10.5281/zenodo.14968656
Timing of MHCfold to process 202 pMHC-I cases, corresponding to X-ray structures from the PDB.<br>In archive: output-data/mhcfold/X-ray.time.txt	Zenodo	https://doi.org/10.5281/zenodo.14968656
Results of comparing 202 MHCfold structural predictions to their X-ray counterparts.<br>In archive: output-data/mhcfold/results-xray/CA-rmsds-full-relaxed.csv	Zenodo	https://doi.org/10.5281/zenodo.14968656
Structural evaluation of 202 MHCfold pMHC-I models.<br>In archive:<br>output-data/mhcfold/results-xray/clashes_full_relaxed.csv<br>output-data/mhcfold/results-xray/ramachandran_full_relaxed.csv<br>output-data/mhcfold/results-xray/omegas_full_relaxed.csv<br>output-data/mhcfold/results-xray/chirality_full_relaxed.csv	Zenodo	https://doi.org/10.5281/zenodo.14968656
MHCfold BA prediction results for 7,726 entries, originating from IEDB.<br>In archive: output-data/mhcfold/ba_classification_results.csv	Zenodo	https://doi.org/10.5281/zenodo.14968656
Timing for MHCfold to predict BA for 7,726 entries, originating from IEDB.<br>In archive: output-data/mhcfold/ba.time.txt	Zenodo	https://doi.org/10.5281/zenodo.14968656
AlphaFold2-FineTune structural predictions for 202 pMHC-I cases, originating from PDB.<br>In archive:<br>output-data/alphafold2-finetune/xray-models-renamed/	Zenodo	https://doi.org/10.5281/zenodo.14968656
Timing for AlphaFold2-FineTune to predict 202 pMHC-I structures.<br>In archive:<br>output-data/alphafold2-finetune/xray-model-timings.txt	Zenodo	https://doi.org/10.5281/zenodo.14968656
Comparison of the AlphaFold2-FineTune 202 predicted structures to their X-ray counterparts.<br>In archive:<br>output-data/alphafold2-finetune/xray-models-CA-rmsd.csv	Zenodo	https://doi.org/10.5281/zenodo.14968656

(*Continued on next page*)

Continued		
REAGENT or RESOURCE	SOURCE	IDENTIFIER
Structural evaluation of 202 AlphaFold2 predicted pMHC-I structures. In archive: output-data/alphafold2-finetune/xray-models-clashes.csv output-data/alphafold2-finetune/xray-models-ramachandran.csv output-data/alphafold2-finetune/xray-models-omegas.csv output-data/alphafold2-finetune/xray-models-chirality.csv	Zenodo	https://doi.org/10.5281/zenodo.14968656
AlphaFold2-FineTune BA predictions for 6,057 pMHC-I cases, representing 7,726 BA data entries from IEDB. In archive: output-data/alphafold2-finetune/ba-model-result.tsv	Zenodo	https://doi.org/10.5281/zenodo.14968656
Timing for AlphaFold2-FineTune to predict 6,057 pMHC-I cases, representing 7,726 BA data entries from IEDB. In archive: output-data/alphafold2-finetune/ba-model-timings.txt	Zenodo	https://doi.org/10.5281/zenodo.14968656
Software and algorithms		
SwiftMHC 1.0.0	This paper	https://doi.org/10.5281/zenodo.18001720
MHCflurry 2.0	O'Donell et al.[9]	https://github.com/openvax/mhcflurry
NetMHCpan 4.1	Reynisson et al.[10]	https://services.healthtech.dtu.dk/services/NetMHCpan-4.1/
MHCfold	Aronson et al.[20]	https://github.com/dina-lab3D/MHCfold
APE-Gen 2.0	Fasoulis et al.[15]	https://github.com/KavrakiLab/Ape-Gen2.0/
PANDORA v2.0.0	Parizi et al.[14]	https://github.com/x-lab-3D/PANDORA
AlphaFold2-FineTune	Motmaen et al.[18]	https://github.com/phbradley/alphafold_finetune
Gibbs Cluster 2.0	Ellis et al.[23]	https://services.healthtech.dtu.dk/services/GibbsCluster-2.0/
Profit 3.1	Martin and Craig[31]	https://mybiosoftware.com/profit-3-1-protein-squares-fitting.html
TM-align 1	Zhang et al.[32]	https://launchpad.net/ubuntu/resolute/amd64/tm-align/20190822+dfsg-3ubuntu1
Pymol 3.1.0 (open source)	DeLano[33]	https://anaconda.org/conda-forge/pymol-open-source
MRS 6	Hekkelman and Vriend[5]	https://mrs.cmbi.umcn.nl/

METHOD DETAILS

Data composition

In this study, we focus on HLA-A*02:01 alleles and 9-mer peptides. For this, 7,726 BA data points were collected from IEDB[27] and 202 X-ray structures were collected from the PDB.[12] OpenMM PDBfixer[29] was used to fill in missing heavy atoms in these X-ray structures in cases where a residue was incomplete. To critically evaluate the generalizability of our predictor and to prevent data leakage, BA and X-ray data were merged together and Gibbs clustered[34] based on their peptide sequences. This was performed using GibbsCluster 2.0, with a cluster similarity penalty (λ) set to 0.8 and a small cluster weight (σ) set to 5. The data points were separated into 10 clusters, each of which was used for testing SwiftMHC using a leave-one-cluster-out cross-validation principle (Figure 3E).

From the left-out cluster of each fold, the BA data points were used to evaluate the BA prediction quality, while the X-ray structures from that same cluster were used for evaluating structure prediction quality. The BA data points of the remaining 9 clusters were used for training (90% of data) and validation (10%). X-ray crystallographic data were excluded from the training and validation datasets to assess the network's 3D modeling performance when trained solely on physics-based 3D model data, though including X-ray data would likely enhance our performance.

Since SwiftMHC requires structural data for training, we generated 3D models for each pMHC-I complex in the BA dataset using PANDORA. For each pMHC-I complex, the 3D model with the lowest molecular probability density function score (molpdf) energy score was selected to be used as input to SwiftMHC.

For the evaluation of BA predictions, we initially used the MHC structures from the corresponding PANDORA models as input to the network model. We later reasoned that using these PANDORA-derived MHC structures instead of the reference experimental structures could introduce a bias due to minor structural variations. However, comparative tests using reference MHC structures showed negligible differences in performance metrics. Therefore, we retained the PANDORA-derived results. For evaluating structure prediction, the MHC structures of the corresponding X-ray structures were used as input to the network model. For evaluating the mutants (Figure S2F) this inconsistency was corrected, and the reference MHC structure from PDB entry 3MRD was used as input.

Data preprocessing

SwiftMHC gradually updates the peptide structure from identity frames (Suppl. mat. Algorithm 4), that is, the starting positions of all peptide residues are at the origin and their N-Cα-C plane overlaps so that they all have the same orientation. This design requires all input structural data in the same orientation, so we superimposed the pMHC-I complex of all X-ray structures and PANDORA 3D models on a reference MHC structure (PDB ID: 3MRD) before training and testing. The reference structure was prepared by isolating the G-domain and placing the geometric center of the MHC binding groove at the origin. All other structures or PANDORA 3D models were structurally aligned to this reference using the PyMOL align command[33] with its default settings: 2.0 Å as outlier rejection cutoff, 5 outlier rejection cycles and using BLOSUM62 for sequence alignment.

Additionally, some values were pre-calculated from the aligned pMHC-I structures, so that SwiftMHC can quickly access them. Those variables were: *ground truth frames*, *ground truth torsion angles*, *MHC proximity matrix*, *peptide and MHC amino acid sequences*, *residue masks* and *ground truth BA*. See Suppl. mat. Subsection 1.2 for details about these variables.

Software and libraries used

SwiftMHC was developed using Python 3.12 and implemented with the PyTorch 2.0.1 library[35] for deep learning functionalities. OpenFold 1.0.0[36] was utilized for key tasks such as FAPE and torsion loss calculations, deriving frames from atomic coordinates, reconstructing atomic coordinates from predicted frames, and performing frame operations. The design of SwiftMHC's IPA modules was also partially inspired by OpenFold code. For handling PDB files, BioPython 1.8.4[37] was employed for parsing and representation. Structural refinements were performed using OpenMM 8.1.1.[4]

Training

10 SwiftMHC network models were trained for each of the 10 peptide clusters, using 10 distinct training datasets. The models were trained in PyTorch[35] 2.3.1 using the Adam optimizer, with a batch size of 16, float32 precision and a learning rate of 10^{-3}. To avoid large loss spikes, gradient norms are clipped to 0.5 at all times. Training is done in two phases, where early stopping can end a phase when stopping criteria are met. In each phase, the early stopping conditions are reset and the model with the lowest validation loss is selected as the initial state in the next training phase.

The training process consists of two distinct phases, each with specific settings (Figure 5B). The goal of the first phase is to enable the model to learn structure prediction and reduce peptide Cα-RMSD from approximately 8 Å to 1–2 Å. During this phase, FAPE and torsion loss are included to guide atomic positions and torsion angles toward those of the true structure. Simultaneously, the BA loss term is incorporated to train the BA prediction module and to speed up the convergence of the 3D structure training. While this phase includes a high maximum epoch count (1000 epochs), an early stopping condition is applied to conclude the phase early if no more improvement in performance is observed.

In the second phase, the model's structure prediction is fine-tuned by introducing violation loss terms during backpropagation (Method S1, Algorithm 10). This phase resembles the fine-tuning phase in AlphaFold2[7] and the associated loss terms encourage interatomic distances, bond lengths and bond angles to approach standard literature values.

During training, an early stopping mechanism is applied to end a training phase and skip to the next phase or to end the training when the conditions are met in the final phase. The conditions for early stopping are based on loss values that are calculated on a validation dataset.

After each epoch the algorithm evaluates the loss values within a range of epochs (i.e., the patience, Figure 5A) unless the number of epochs passed in that training phase is less than the patience value. We configured this patience value to be 50. For example, at epoch 50 we name the range 0–50 the *Frame of Patience*. At epoch 51, this frame shifts to the range 1–51 and so on.

Sometimes the high spikes of losses interrupts the early stopping. So we need to first smooth out the loss values within each Frame of Patience. Within each Frame of Patience the algorithm searches for outliers. Whether a loss value is an outlier or not is determined by the interquartile range (IQR). A loss value is considered an outlier if it lies more than 1.5 times the IQR above the third quartile (Q3) or more than 1.5 times the IQR below the first quartile (Q1). These outliers are replaced by the median value within the Frame of Patience, so that they cannot block early stopping.

After replacing the outliers with median values, linear regression is performed to find the best straight line through the loss values within a Frame of Patience. The algorithm stops a training phase early if the slope of this line is nearly horizontal and its value lies between -1×10^{-4} and 1×10^{-4}, indicating the predictive performance on the validation set is not improving any more.

Post processing the resulting structures

Once a pMHC-I structure is predicted and PDB to disk writing is enabled, SwiftMHC optionally runs OpenMM[29] to minimize its energy using an amber99sb force field. To prevent chirality issues in non-glycine backbones, the hydrogen atom attached to the chiral Cα atom is added by SwiftMHC before running OpenMM. All other hydrogen atoms are added by OpenMM.

SOTA methods

We compared SwiftMHC against several SOTA methods (Table 1) on BA prediction and/or structure prediction qualities. Five methods were compared for their speed and BA prediction quality: SwiftMHC, AlphaFold2-FineTune,[18] MHCflurry 2.0[9] (both original and retrained models, see additional resources for the download URL), netMHCpan 4.1 and MHCfold.[20] The AUC was used as a BA quality metric. We classified a peptide as binding to its MHC if IC_{50} or K_d was below 500 nM and as non-binding otherwise. Six methods were compared for reproducing the 202 X-ray structures: SwiftMHC, SwiftMHC-OpenMM, AlphaFold2-FineTune,[18] PANDORA,[14] APE-Gen 2.0[15] and MHCfold.[20]

AlphaFold2-FineTune is a modified version of AlphaFold2, where the weights and biases of the network model have been fine-tuned using pMHC BA and X-ray pMHC structural data. AlphaFold2-FineTune achieves faster processing times than standard AlphaFold2 by omitting template searching and multiple sequence alignments. Instead, it requires the user to provide a query-to-template alignment. A second reason for the increased speed of AlphaFold2-FineTune compared to the standard AlphaFold2 tool is that it does not refine its output structures using an Amber forcefield in OpenMM, which is typically employed for energy minimization in the standard AlphaFold2 pipeline.

We used the 202 X-ray HLA-A*02:01 9-mer structures as templates for AlphaFold2-FineTune. For selecting templates, the Gibbs clustering was used. This means that for each target peptide from a specific cluster, the pMHC-I X-ray structures from the other nine clusters were selected as the template set. To create MHC alignments as input for AlphaFold2-FineTune, the target G-domain MHC sequences were aligned to the template MHC sequences using the BioPython pairwise alignment tool[37] with default settings (Needleman-Wunsch algorithm, match score 1, mismatch score 0, gap scores 0). For peptide alignment, the 9-mer peptides were aligned in a one-to-one manner (i.e., position one aligned with position one, position two with position two, and so on). Other components of the X-ray structures, such as β2-microglobulin and the α3 domain, were excluded from the alignments. The time to make these alignments was not included in the computation time of AlphaFold2-FineTune. The quality of structural predictions was assessed by comparing the 202 X-ray HLA-A*02:01 9-mer structures against 3D models predicted by AlphaFold2-FineTune. The total runtime required to process these 202 entries was documented.

To assess the quality of BA predictions, we compared 7,726 experimental BA values against predictions generated by AlphaFold2-FineTune. Due to redundancy in the pMHC-I sequences (sometimes there are multiple BA values per complex) and the computational demands of running AlphaFold2-FineTune, we ran AlphaFold2-FineTune on the 6,057 non-redundant data. The total runtime required to process these 6,057 entries was documented. This evaluation aimed to measure AlphaFold2-FineTune's ability to differentiate true binders (experimental K_d or IC_{50} < 500 nM) from true non-binders, following a similar approach to that described in.[18] For each pMHC-I entry, the negative mean of the MHC-to-peptide and peptide-to-MHC position-aligned error (PAE) values was used as a predictive score. These scores were compared with experimental BA values to calculate the AUC for each cluster. The prediction scores that correspond to the repeated sequence entries were replicated, ensuring each experimental BA value was compared to its corresponding prediction score.

MHCfold[20] is a deep learning tool that first predicts the structures of both the peptide and the MHC molecule from their sequences using convolutional neural networks (CNNs). It then employs a modified version of multi-headed attention along with a structure-derived distance matrix, similar to IPA, to predict BA as either binding or non-binding. To evaluate the BA and structural prediction quality of the MHCfold algorithm, we used the MHC G-domain sequences along with corresponding peptide sequences as inputs. MHCfold's reliance on side-chain modeling and PDB generation may slow down processing. For fair comparison with SwiftMHC-BA, these features were disabled during BA evaluation (referred to as "MHCfold-BA"). We used MHCfold to predict BA for all 7,726 data points and calculated the AUC based on the predictions. The total runtime was measured. Additionally, MHCfold was used to generate pMHC structures for all 202 X-ray complexes. For this. MHCfold was set to predict both the 3D structure and BA and to use MODELLER for reconstructing the side chains. The total runtime for creating the 202 3D models, including the time required for MODELLER, was measured. The resulting 3D models were subsequently evaluated.

PANDORA is a 3D modeling approach, specialized to predict the structure of pMHC complexes for both class I and II. It does so by using a database of MHC templates and a multiple sequence alignment of their conserved domains. It uses homology modeling to build the model and keeps the peptide's anchors restrained. To evaluate the performance of PANDORA[14] structure predictions, we made it generate pMHC-I 3D models for each of the 202 available X-ray structures. PANDORA was configured to output 20 3D models per case (the default setting) and the 3D model with the lowest molpdf score was selected. To prevent data leakage, templates containing peptides identical to those in the target 3D model were excluded from the 3D modeling process. To properly measure the process time consumption, PANDORA was executed within a multiprocessing pool configuration with 32 cores. The total processing time required for the pool to complete all 202 3D models was recorded.

ApeGen 2.0 is a tool that generates an ensemble of peptide-MHC conformations within a chosen number of iterations. In each iteration, it first searches the Protein DataBank (PDB) for a suitable MHC template, then anchors the atoms of the first and last two residues of the given peptide as in the template. Between these anchor positions, it samples for peptide backbone conformations

that geometrically fit best using Random Coordinate Descent (RCD).[38] On the resulting backbone conformations, OpenMM PDBFixer[29] samples for side chains to complete the structure. Finally SMINA[39] energy minimization is performed on both the peptide and MHC to fix steric clashes. The best resulting structure may be used as input for the next iteration.

To evaluate the structure prediction performance of APE-Gen 2.0,[15] we employed this software to 3D model the pMHC complex for each of the 202 available X-ray structures. APE-Gen 2.0 was set to output 20 3D models per case (the default setting) and the 3D model with the lowest APE-Gen 2.0 Affinity score was selected. To prevent data leakage, templates containing identical peptides to those in the target 3D model were excluded from the set. By default, an APE-Gen 2.0 process runs in a Docker container, using 8 CPU cores to pool the RCD and SMINA computations. For comparing APE-Gen 2.0's runtime with other SOTA methods on the same software and hardware settings, we ran APE-Gen 2.0 outside a Docker container as a single process with 32 cores. The total processing time for APE-Gen 2.0 to generate all 202 3D models was recorded. These resulting 3D models were subsequently evaluated.

MHCflurry 2.0[9] is a SOTA sequence-based BA predictor which consists of an ensemble of MLPs. We retrained MHCflurry 2.0 on our 7,726 BA data points by a 10-fold leave-one-cluster-out cross-validation approach for each of the 10 peptide clusters (Figure 3E). The retraining process was carried out using the allele-specific scripts, provided by MHCflurry 2.0. In each iteration, one cluster was held out as the test set, while the remaining nine clusters were used to create a training set (90%) and a network model selection set (10%). The selected network models were subsequently used to predict BA for the peptides from the remaining cluster. The predicted BA values from MHCflurry 2.0 were compared to experimental BA values (binding or non-binding) by calculating AUC. The processing time was measured.

QUANTIFICATION AND STATISTICAL ANALYSIS

Computational efficiency

For speed purposes, several design choices were made for SwiftMHC. Since the MHC structure remains fixed, its backbone frames and distance matrix can be precalculated and reused throughout the prediction process. This avoids repeated computations. The preprocessed data was stored in an HDF5 file format to facilitate quick access and efficient data management. For an efficient cross attention computation, MHC residues that do not lie close to the peptide are masked out, meaning that they do not contribute to any operation in that module (Figure S4E). PDB structures are generally only written to disk on demand and never during training. In addition, we replaced the traditional 3×3 rotation matrix operations with quaternion operations, reducing numerical computations and improving efficiency.

The *time consumption* of executing a fully trained SwiftMHC network model was measured on a single NVIDIA A100 GPU (40 GiB HBM2 memory with 5 active memory stacks per GPU) and 32 Intel Xeon Platinum 8360Y CPUs and limited to using 40 GiB 3.2 GHz DDR4 memory. We made SwiftMHC allocate 16 worker processes to read input data and 16 builder processes to write out structural data in PDB format. For comparing speed, SOTA methods were executed on the same hardware configuration as SwiftMHC, except for AlphaFold2-FineTune, which required a 100 GiB memory limit instead of 40 GiB. We measured the exact time duration of every method to complete with one-hundredth of a second precision.

The training data overlap between AlphaFold2-FineTune, MHCfold and our test data

We did not retrain AlphaFold2-FineTune, because of limited time and computational resources. Instead, we utilized a pretrained network model obtained from the publicly available source (see additional resources for the download URL). We identified that the 202 X-ray structures in our test set represent 118 unique pMHC-I's of which 34 were part of AlphaFold2-FineTune's training dataset. Moreover, AlphaFold2-FineTune was fine-tuned from the AlphaFold2 model, which had been trained on PDB entries deposited before the 30th of April 2018,[18] which dates after the deposition date of 98 of those 118 unique pMHC-I's. Therefore it is likely that these structures were included in AlphaFold2's training dataset. IThat means that in total 105 of the 118 unique pMHC-I's were used to train AlphaFold2-FineTune (see also Figure 3C).

MHCfold could not be retrained for this evaluation due to the absence of a training script. Therefore, we utilized the pretrained network model available from the public source (see additional resources for the download URL) to generate MHCfold predictions for the evaluation. For structural prediction, this model was trained on 431 unique pMHC-I's deposited before November of 2021 with resolution higher than 3.5 Å.[20] This includes all 118 unique pMHC-I's in our X-ray test dataset (Figure 3C) and therefore it is likely that these structures were included in MHCfold's training dataset.

For BA prediction, both the AlphaFold2-FineTune and MHCfold models were reportedly trained on the netMHCpan 4.1 training set[18,20] which incorporates pMHC binding data from the IEDB. Since our datasets were also derived from the IEDB, it is plausible that some, if not all, of our test data was included in the training sets of these two pretrained models.

These overlaps could have inflated the results of AlphaFold2-FineTune and MHCfold.

Evaluation of BA prediction

We evaluated the performance of each BA predictor using the area under the receiver operating characteristic curve (AUC) as the evaluation metric. To meet the binary classification requirements of AUC, we categorized peptides as binding to their MHC when their ground truth IC_{50} or Kd values were below 500 nanomolar (nM) and as non-binding otherwise.

Evaluation of structure prediction

To calculate *Cα-RMSD*, all the 3D models were superposed in ProFit[31] to the MHC G-domains of the corresponding X-ray structures. To account for structural variations, the two most N-terminal residues were omitted from the G-domain superposition due to their absence in some structures. Similarly, the C-terminal residue was excluded from the superposition analysis because its orientation varies significantly across different X-ray structures. The remaining portion of the HLA-A*02:01 sequence, encompassing the amino acids (IMGT numbering 3–179) was utilized for superposition. ProFit was used to calculate RMSD for the peptide Cα atoms to quantify the differences between the predicted 3D models and their corresponding X-ray structures. This analysis was performed for each 3D model across all methods to identify which 3D models exhibited the greatest similarity to their respective X-ray counterparts.

Chiralities were determined per amino acid from the positions of the N, C and Cα atoms around the Cα atoms using Numpy.

Ramachandran plots were generated by calculating the backbone ψ and φ torsion angles for each residue in the peptide using BioPython.[37]

ω torsion angles of the peptide typically approximate 180°. To verify this, the angles were calculated for each peptide bond across all 3D structures using NumPy. The distributions of ω angles from the predicted 3D models and the X-ray structures were plotted to identify any differences.

van der Waals clashes are considered energetically unfavorable and should be minimal. To identify overlaps between the van der Waals radii of non-bonded atom pairs in the peptide, the distances between atoms in the 3D structures were calculated using NumPy. A clash was defined as occurring when two atomic centers were closer than the sum of their van der Waals radii, with exceptions made for 1) protons for which the position is usually determined by calculation, not prediction; 2) for atoms within the same residue, as those are usually either part of a ring system or connected to each other with zero, one or two atoms in between; 3) for the backbone atoms of two connected residues; 4) two connected residues where one is proline, of which the side chain is connected to the backbone; 5) two cysteines that can be disulfide bonded. Any other pair of atoms that were positioned too close to each other was considered clashing. The number and severity of clashes were compared across all 3D models.

ADDITIONAL RESOURCES

Download URL for AlphaFold2-FineTune pretrained network model: https://files.ipd.uw.edu/pub/alphafold_finetune_motmaen_pnas_2023/datasets_alphafold_finetune_v2_2023-02-20.tgz

Download URL for MHCFold pretrained network model: https://github.com/dina-lab3D/MHCfold/blob/main/v7_date_5_9_2022.zip

Download URL for MHCflurry 2.0 pretrained network model: https://github.com/openvax/mhcflurry/releases/download/pre-2.0/models_class1_presentation.20200611.tar.bz2

Cell Reports Methods, Volume *6*

Supplemental information

A high-speed attention network for MHC-bound peptide identification and 3D modeling

Coos A.B. Baakman, Giulia Crocioni, Cunliang Geng, Daniel T. Rademaker, David Frühbuß, Yannick J.M. Aarts, and Li C. Xue

Supplementary information

Contents

Figure S1: Distribution of backbone torsion angles of 3D models generated by SOTA approaches, related to Fig. 4 2
Figure S2: BA prediction metrics, correlation and training/test set overlap, related to Fig. 3A 4
Figure S3. Visualization of cross-attention weights in SwiftMHC, related to the Discussion 6
Method S1 - related STAR Methods 8
1. Data 8
1.1 Data Composition 8
1.2 Data Preprocessing 8
1.2.1 Ground Truth Frames for Backbone and Side Chain Conformations 8
1.2.2 The MHC Proximity Matrix 10
1.2.3 Amino Acid Sequences 10
1.2.4 Sequence Masks 11
1.3 Data Representation 11
2 SwiftMHC Algorithm Design 12
2.1 The Main module 12
2.2 Module 1: MHC Self Invariant Point Attention 14
2.3 Module 2: Peptide Self Attention 15
2.4 Module 3: Cross Attention Structure module 18
2.4.1 Cross Invariant Point Attention 21
2.4.2 Backbone Updating 23
2.4.3 Torsion Angles and Rigid Groups 24
2.5 Module 4: Residue-wise Binding Affinity Predictor 26
2.6. Loss Terms 26
Result S1 - SwiftMHC's robustness to structural variation in MHC molecules, related to Fig. 3 30
3. References 31

Figure S1: Distribution of backbone torsion angles of 3D models generated by SOTA approaches, related to Fig. 4.

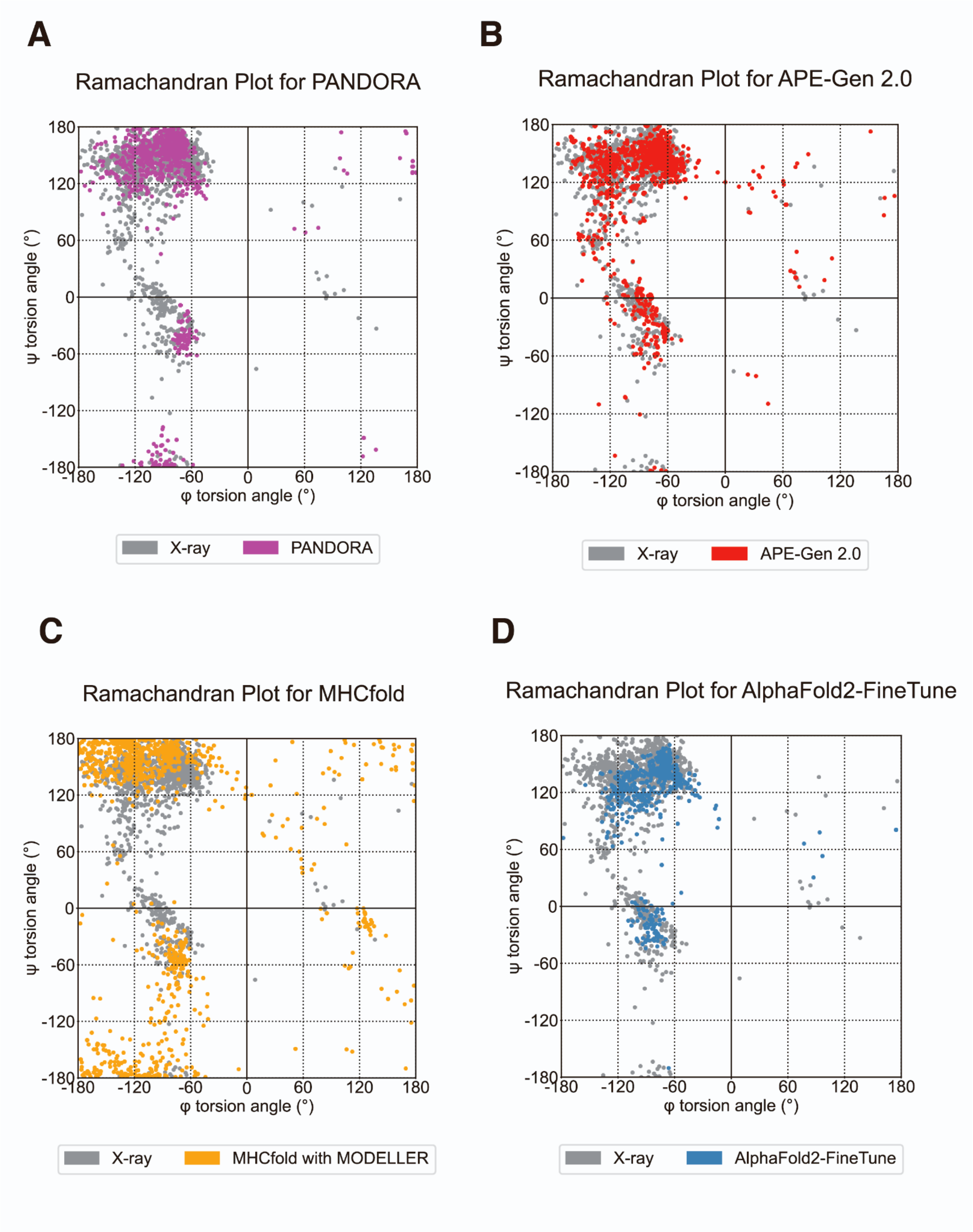

Figure S1. Distribution of backbone φ and ψ torsion angles of 3D models generated by SOTA approaches for the 202 X-ray test cases, related to Fig. 4. The plots have been overlaid with φ and ψ torsion angle data from the 202 X-ray structures. **A.** PANDORA models **B.** APE-Gen 2.0 models **C.** MHCfold models **D.** AlphaFold2-FineTune models

Figure S2: BA prediction metrics, correlation and training/test set overlap, related to Fig. 3A.

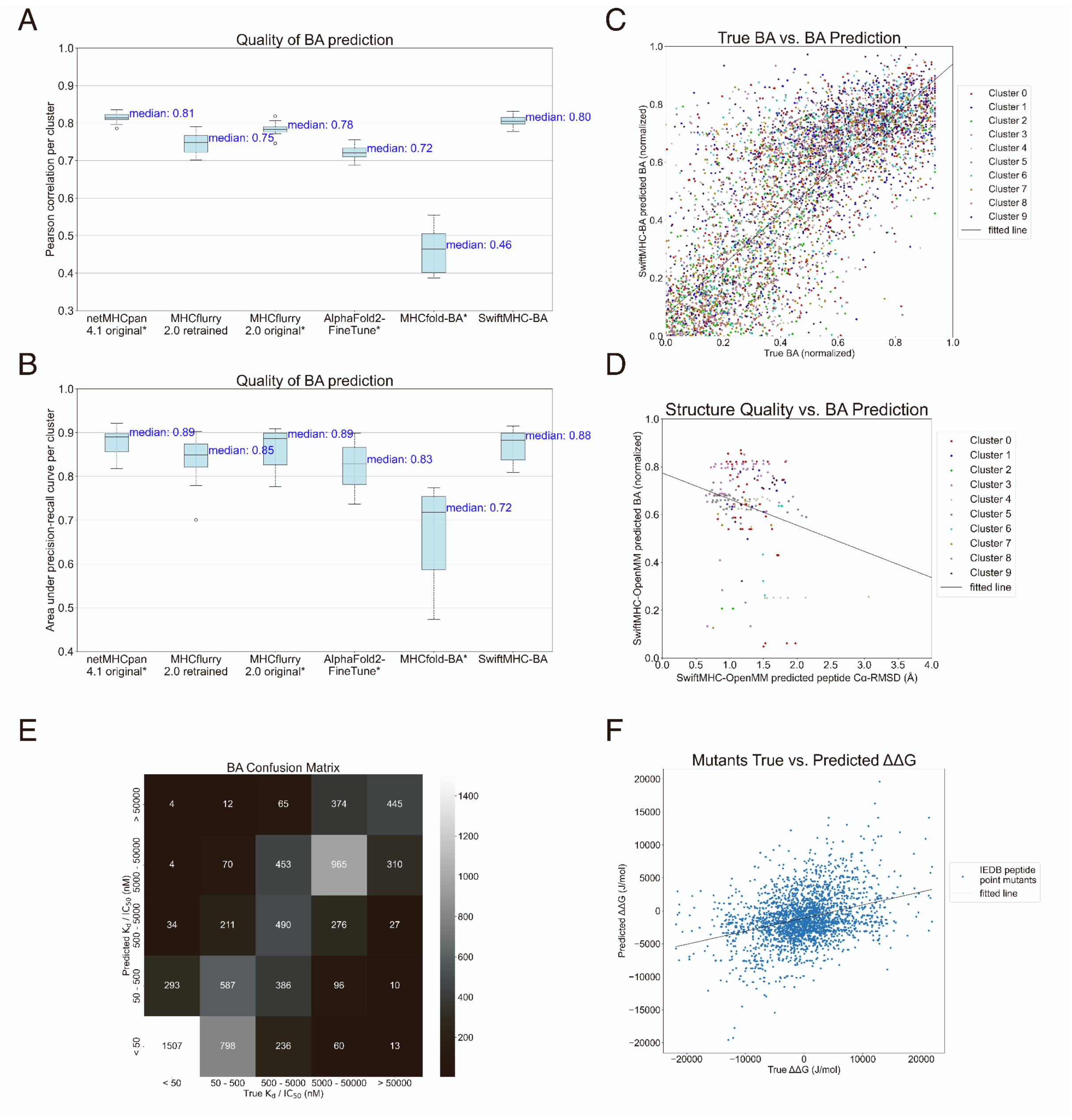

Figure S2. BA prediction metrics, correlation and training/test set overlap, related to Fig. 3A. BA prediction performance measured by Pearson correlation. Box plots show the distribution of Pearson correlation coefficients across 10 clusters of HLA-A*02:01 9-mer peptides. **B.** BA prediction performance measured by area under the precision-recall curve (AUPR). Box plots show the distribution of AUPR across 10 clusters of HLA-A*02:01 9-mer peptides. **C.** Scatter plot between SwiftMHC predicted BA and true BA for the 7,726 IEDB benchmark cases. The line of best fit was computed using least squares regression, with a pearson correlation of 0.82. BA values on the both the horizontal and vertical axes represent BA values, calculated as $1.0 - \log_{50000}(K_d)$ or $1.0 - \log_{50000}(IC_{50})$. **D.** Scatter plot between predicted BA and backbone RMSD for the 202 X-ray benchmark cases. The line of best fit was computed using least squares regression, with a pearson correlation of 0.22, indicating that predicted structure quality is weakly related with BA prediction. BA values on the vertical axis represent SwiftMHC

predictions, calculated as $1.0 - \log_{50000}(K_d)$ or $1.0 - \log_{50000}(IC_{50})$. **E.** Confusion matrix showing the 10-fold test set BA prediction distribution in the low and high affinity ranges. **F.** Correlation between true and predicted ΔΔG for 2,838 single-point mutants of 9-mer peptides. Each data point represents the change in ΔG when the peptide binds to MHC, calculated as RT $\log(K_d)$ or RT $\log(IC_{50})$, with R=8.314 J K^{-1} mol^{-1} and T=298.15 K. The change in ΔG (ΔΔG) is displayed per mutant, relative to its wild-type peptide. True K_d or IC_{50} data originates from the 7,726 data points taken from the IEDB. Predictions were made using SwiftMHC models trained on the same data fold as the corresponding wild-type. The Pearson correlation between true and predicted ΔΔG values is 0.33.

Figure S3. Visualization of cross-attention weights in SwiftMHC, related to the Discussion.

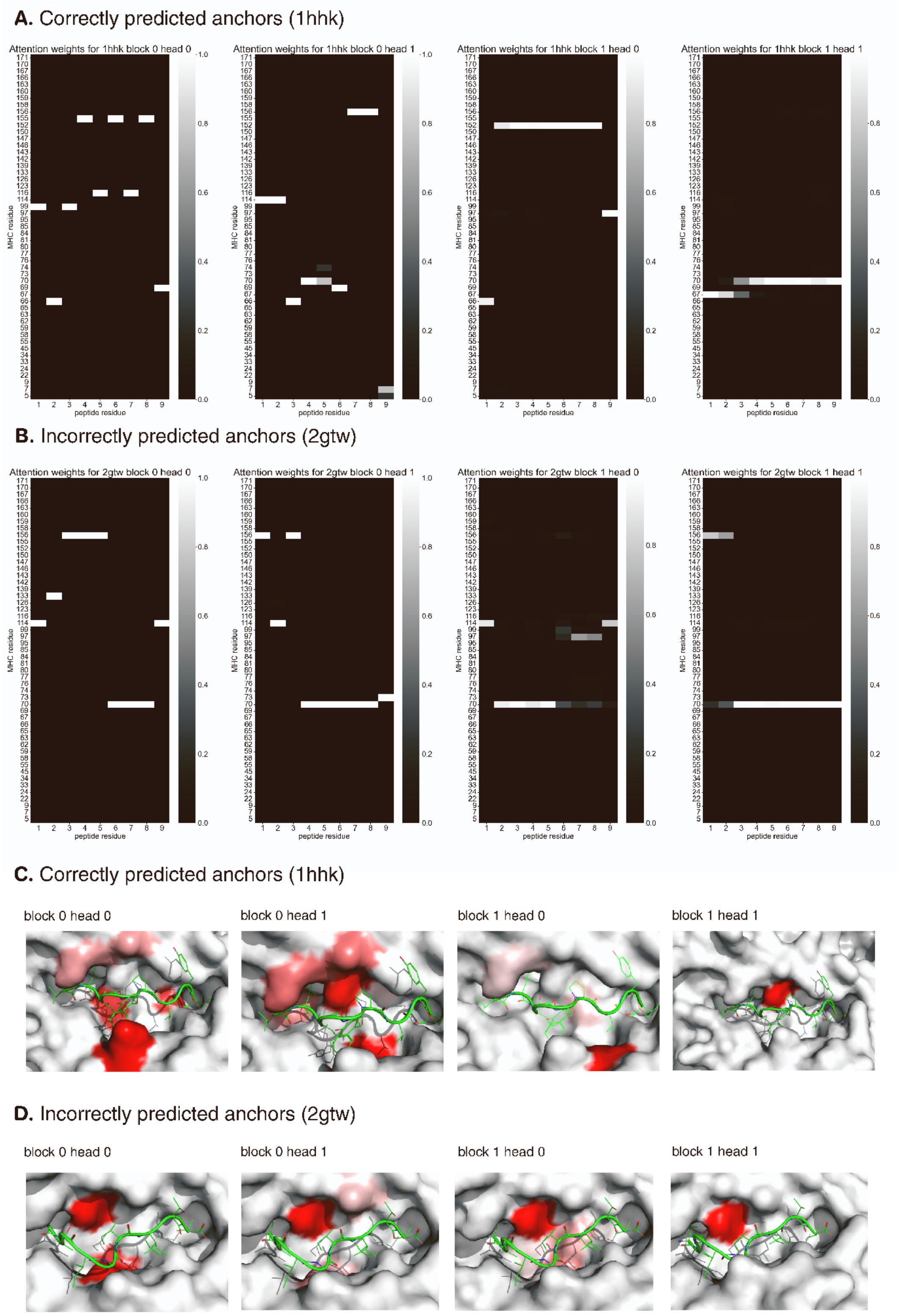

Figure S3. Visualization of cross-attention weights in SwiftMHC, related to the Discussion. A. Attention weight matrices from two transformer blocks (two heads each) for the 1HHK structure (peptide: LLFGYPVYV; allele: HLA-A*02:01; X-ray anchor residues: positions 2 and 9), for which SwiftMHC correctly predicted the anchors **B.** Attention weight matrices for the 2GTW structure (peptide: LAGIGILTV; allele: HLA-A*02:01; anchor residues: positions 1 and 9, for which SwiftMHC wrongly predicted the anchors **C and D.** Attention weights on the surface of the MHC structure of 1HHK and 2GTW, with regions colored in red according to the normalized sum of attention weights (each value was divided by the max sum of all MHC residues, darker red indicates higher attention).

The peptide has been colored green. Notably, the network correctly focused on the P2 pocket in 1HHK when the anchor residues are accurately predicted (**C**), whereas such focus on anchor pockets is absent in 2GTW, whose anchor was wrongly predicted (**D**). This indicates that attention patterns may provide a signal for whether the anchors are correctly predicted, although systematic analysis is needed to confirm this.

Method S1 - related STAR Methods

1. Data

1.1 Data Composition

To examine the distribution of peptide lengths in pMHC class I (pMHC-I) complexes, we downloaded 3,064,594 MHC-I BA data entries from the IEDB[1]. These entries encompass complexes of various peptide lengths and MHC-I alleles, beyond just 9-mer peptides and the HLA-A*02:01 allele. 9-mer peptides occur most frequently in this database.

SwiftMHC is trained, validated and tested on a subset of these 3,064,594 entries. This subset consists of 7,726 HLA-A*02:01 9-mer BA data from the IEDB[1]. Because SwiftMHC requires structural data to train on, these entries were represented by 7,726 PANDORA models. Additionally SwiftMHC was validated on 202 X-ray structures. To avoid data leakage, this data was combined and Gibbs clustered[2] based on peptide sequence similarities into 10 clusters of roughly the same size. The distribution of clustered data is shown in **Fig. 3E.**

1.2 Data Preprocessing

Before SwiftMHC can be trained from the input data (**Subsection 1.1**) it is preprocessed. During preprocessing, several variables are precalculated and stored in HDF5 (https://www.hdfgroup.org/solutions/hdf5/) format with LZMA compression (http://www.7zip.org/7z.html) for every pMHC complex: *ground truth frames, ground truth torsion angles, a MHC proximity matrix, amino acid sequences, sequence masks* and *ground truth BA*. This section describes each of these in detail. For ground truth structural data, either X-ray 3D structures from the RCSB PDB[3] or PANDORA[4] 3D models were used. Ground truth BA data were extracted from the IEDB[1].

1.2.1 Ground Truth Frames for Backbone and Side Chain Conformations

The *geometry* of backbone and side chains of both the MHC and peptide are represented by local frames (or local coordinate frames). Local frames are attached to objects (amino acids here) to describe their orientation and position relative to a global frame (**Fig. S4A**). This global frame represents the xyz-coordinate system of the pMHC structure. The local frames allow us to describe the movement of residues

or side-chains. Each local frame T_i holds a translation vector $\vec{t}_i$ and a unit quaternion q_i that describes a rotation: so $T_i = (q_i, \vec{t}_i)$.

Applying a transformation to global space means that the point vector is first rotated by the quaternion and then translated by the corresponding translation vector. In this document, such a frame transformation is indicated by the ∘ operator.

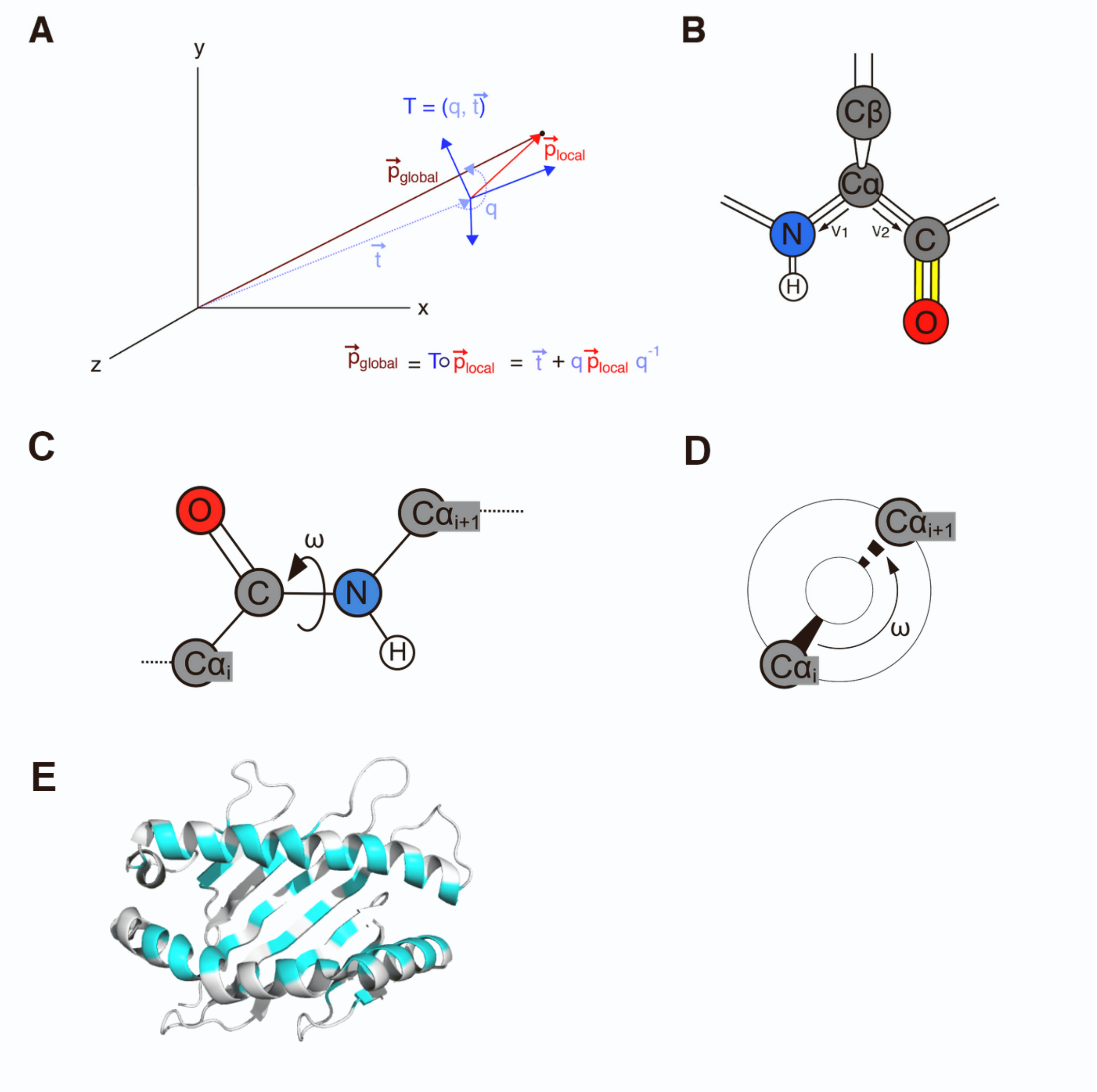

Figure S4: Explanation of Geometry, related to Fig. 2A. Local frames are coordinate systems attached to each amino acid to define their position and orientation relative to the global reference coordinate system. A point $\vec{p}$ in the local frame space (blue axes) can be converted to the global space (black axes) by transforming it by the frame T, consisting of a rotation quaternion q and a translation vector $\vec{t}$. Such a transformation is indicated by the ∘ operator. **B.** Explanation how a backbone local frame for one residue is defined from the atomic positions of backbone N, Cα and C. The bond between atoms N and Cα ($\vec{v}_1$) determines the direction of the x-axis and the three atoms together lie in the xy-plane ($\vec{v}_1$ and $\vec{v}_2$), from which the y-axis is derived. The z-axis is the normalized cross product between the x and y axis and is perpendicular to this xy-plane, $\vec{v}_1$ and $\vec{v}_2$. **C.** The ω torsion angle for

the peptide bond. A peptide bond between amino acid residue i and i+1 is shown. The ω torsion angle is measured from the positions of $Cα_i$ and $Cα_{i+1}$ around the C-N bond. **D.** A Newman projection of the ω torsion angle. **E.** HLA-A*02:01 residues that participate in Cross IPA (cyan). PDB entry 3MRD is used here and the peptide is not shown for easy visualisation.

In order to predict structure, SwiftMHC predicts frames, but it also takes frames as ground truth during training. These *ground truth frames* are derived from 3D protein structures and include translations relative to a defined origin. To ensure consistency across all processed structures, the origin was set to the center of the MHC groove, and all structures were superposed accordingly. Additionally, the superposed structures were aligned to share the same orientation.

During preprocessing, the 7 ground truth frames and torsion angles are calculated from the atomic positions of each residue. These frames are always defined from three atomic positions, where $\vec{t}_i$ is the position of one of the atoms and q_i is calculated to represent the orientation of the other two atoms around this central atom (**Fig. S4B**). For the backbone truth frames $\{T_i^{true}\}$, we use the N,Cα,C atoms. The seven side chain ground truth frames $\{T_i^{true, sidechain}\}$ are based on rotatable covalent bonds, that define the conformation of an amino acid by at most seven torsion angles φ, ψ, ω, χ_1, χ_2, χ_3, χ_4. Specifically, we use the two atoms of each torsion bond and the next atom after that. Because for some torsion angles in some amino acids there is a 180° symmetry in the side chain, we also compute a series of alternative torsion angles and frames to be stored in separate arrays: $\{T_i^{alt\ truth}\}$ and $\{T_i^{alt\ truth, sidechain}\}$. For smaller amino acids with less than 7 torsion angles, the nonexistent frames and angles are masked out to be ignored by the algorithm.

1.2.2 The MHC Proximity Matrix

For every residue pair in the MHC, the distance between the closest two heavy atoms is determined. These distance values $d_{j\hat{j}}$ are converted to proximity values $z_{j\hat{j}}$ by the formula: $z_{j\hat{j}} = \frac{1}{1 + d_{j\hat{j}}}$. These values are combined into an $r_j \times r_j \times 1$ proximity matrix, where r_j is the number of residues in the MHC G-domain. This matrix $z_{j\hat{j}}$ is input to the MHC Self IPA module (**Subsection 2.2**).

1.2.3 Amino Acid Sequences

Peptide and MHC sequences are represented as one-hot encodings, where the corresponding number of the amino acid is set to 1 and the other numbers are set to 0. Each of the 20 amino acid types is represented by a one-hot encoded $c_s \times 1$

vector with $c_s = 32$ and therefore a sequence of r residues is represented by a $r \times c_s$ array of vectors. For the dimension c_s, the encoding scheme uses a number larger than 20 to allow flexibility for representing non-canonical amino acids. This approach ensures compatibility with potential future extensions involving non-standard amino acid residues, such as post-translationally modified variants.

Additionally each amino acid in a sequence is associated with an atomic mask and a list of atom names. Atomic masks are essential for structural loss functions, as they specify which xyz positions should be included in the calculations. Atom names are necessary for converting arrays of xyz positions into actual atoms when generating PDB-formatted 3D structures.

1.2.4 Sequence Masks

For computational efficiency, SwiftMHC works with masked MHC structures, meaning that a selection is made for residues to be included in the attention modules. For MHC Self IPA calculation (**Subsection 2.2**), only residues within the G-domain (IMGT[5] numbers 2-181) are used. For Cross IPA between peptide and MHC (**Subsection 2.4.1**), only MHC residues within 10 Å of the peptide (as observed in any X-ray structure) are included (**Fig. S4E**).

1.2.5 Ground Truth BA

From the IEDB[1] experimental BA data, both IC_{50} and K_d values are converted to 1 - $log_{50000}(IC_{50})$ or $1 - log_{50000}(K_d)$ respectively as was done in [6]. The resulting values serve as the ground truth in the BA loss function in regression-based BA training and validation.

The class label (binding or non-binding) is also derived from this BA value: pMHC complexes are considered binding if their IC_{50} or K_d is below 500 nM. This class label is used for calculating the receiver operating characteristic area under the curve (AUC).

1.3 Data Representation

SwiftMHC's main module predicts peptide 3D structures and BA values (**Subsection 2.1**). The module processes preprocessed input data, including the MHC and peptide *sequences*, the *local backbone frames* of the MHC, and a *proximity matrix* specific to the MHC.

Initially the peptide and MHC are represented by two *sequences* of one-hot encoded amino acids (**Subsection 1.2.3**). We denote the peptide sequence as $\{s_i\}$ and the MHC sequence as $\{s_j\}$. These vectors serve as input to the Self Attention modules (**Algorithms 2** and **3**) that will output updated versions of them. We denote the array

of *local backbone frames* as $\{T_i\}$ for the peptide and $\{T_j\}$ for the MHC. The MHC frames $\{T_j\}$ are set to be equal to the ground truth (**Subsection 1.2.1**) and they are kept fixed during inference. The backbone frames for the peptide $\{T_i\}$ are initially all placed at the center of the MHC groove with uniform orientation, but they are iteratively refined during inference to approach their ground truth. Specifically to the MHC an $r_j \times r_j \times 1$ *proximity matrix* $\{z_{j\hat{j}}\}$ (**Subsection 1.2.2**) is used. This matrix serves as input to the MHC Self Invariant Point Attention Module (**Subsection 2.2**).

The output of the Main module consists of a numerical BA value and a peptide structure. This peptide structure is represented by the xyz positions of the peptide atoms themselves. These are represented by a $r_i \times n \times 3$ array of vectors $\{\vec{x}_i^{all}\}$, where r_i represents the number of residues in the peptide sequence and n represents the maximum number of atoms per residue ($n = 14$). For small amino acids like alanine, not all of these vectors are used. Atomic masks are used in the loss function to make sure that the positions of nonexistent atoms do not count.

2 SwiftMHC Algorithm Design

2.1 The Main module

The neural network of SwiftMHC consists of a Main module (**Algorithm 1**). This module takes as input a major histocompatibility complex (MHC) structure and a peptide sequence. Its outputs are the *BA* value and an all-atom pMHC 3D model.

Algorithm 1 MainModule

procedure MainModule ($\{s_i\}, \{s_j\}, \{z_{j\hat{j}}\}, N_{blocks} = 2,$
$\{T_j\}, \{T_i^{true,all}\}, \{T_i^{alt\ truth,all}\},$
$\{\vec{x}_j^{all}\}, \{\vec{x}_i^{true,all}\}, \{\vec{x}_i^{alt\ truth,all}\},$
$\{\vec{\alpha}_i^{true,all}\}, \{\vec{\alpha}_i^{alt\ truth,all}\}, \mathrm{BA}^{true}$):

    # This layer applies weighted normalization over all pairs
    # of MHC residues. It is likely to be removed in future updates.
    $\{z_{j\hat{j}}\} \leftarrow \mathrm{LayerNorm}(\{z_{j\hat{j}}\})$

    # MHC self attention (module 1).
    # SelfIPA and LayerNorm weights are shared across blocks.
    # j and $\hat{j}$ correspond to MHC residues.
    for all $l \in [1, ..., N_{blocks}]$ **do**
        # For SelfIPA see Algorithm 2.
        $\{s_j\} += \mathrm{SelfIPA}(\{s_j\}, \{z_{j\hat{j}}\})$
        $s_j \leftarrow \mathrm{LayerNorm}(\mathrm{Dropout}_{0.1}(s_j))$
    end for

    # Peptide self attention (module 2).
    # Each block has its own set of PeptideSelfAttention weights and biases.
    # LayerNorm weights are shared across blocks.
    # i corresponds to a peptide residue.
    for all $l \in [1, ..., N_{blocks}]$ **do**
        # For PeptideSelfAttention see Algorithm 3.
        $\{s_i\} += \mathrm{PeptideSelfAttention}(\{s_i\})$
        $s_i \leftarrow \mathrm{LayerNorm}(\mathrm{Dropout}_{0.1}(s_i))$
    end for

    # Perform Cross Attention (module 3) and predict structure.
    # For CrossAttentionStructureModule see Algorithm 4.
    $\{\vec{x}_i^{all}\}, \{T_i^{all}\}, \{\vec{\alpha}_i^{all}\}, \{s_i\} =$
        CrossAttentionStructureModule ($\{s_i\}, \{s_j\}, \{T_j\}$)

    # Normalize before BA prediction
    $s_i \leftarrow \mathrm{LayerNorm}(\mathrm{Dropout}_{0.1}(s_i))$

37: # Predict BA (module 4).
38: # BA $\in \mathbb{R}$
39: # For BindingAffinityPredictor see Algorithm 8.
40: $\text{BA} = \text{BindingAffinityPredictor}\ (\ \{s_i\})$
41:
42: # Loss computation is optional.
43: # For CalculateLoss, see Algorithm 9
44: $\mathcal{L}_{\text{tot}} = \text{CalculateLoss}(\text{BA}, \text{BA}^{true},$
45: $\{\vec{x}_i^{all}\}, \{\vec{x}_j^{all}\}, \{\vec{x}_i^{true,all}\}, \{\vec{x}_i^{alt\ truth,all}\},$
46: $\{T_i^{all}\}, \{T_i^{true,all}\}, \{T_i^{alt\ truth,all}\},$
47: $\{\vec{\alpha}_i^{all}\}, \{\vec{\alpha}_i^{true,all}\}, \{\vec{\alpha}_i^{alt\ truth,all}\}\ )$
48:
49: **return** BA, $\{\vec{x}_i^{all}\}$, $\mathcal{L}_{tot}$
50: **end procedure**

2.2 Module 1: MHC Self Invariant Point Attention

Within the Main module, the MHC Self Invariant Point Attention (Self IPA) module operates on the MHC structural data (**Fig. S5, Algorithm 2**). The purpose of this module is to encode each MHC residue-based on its amino acid type and its structurally neighboring amino acids. This module draws inspiration from **AlphaFold2[7] Suppl. Algorithm 22 - Invariant Point Attention,** which enables amino acid residues in a sequence to update each other based on their features and structural geometry. The code for this module is a modified version of the Openfold[8] IPA implementation. It was designed to represent and process interactions between amino acids. The inputs of the module are the one-hot encoded MHC amino acid sequence $\{s_j\}$ and the proximity matrix $\{z_{j\hat{j}}\}$. For computational efficiency purposes, the MHC structure is masked so that only residues of the G-domain are included in the computation. The computed attention weights $a^h_{j\hat{j}}$ are used for updating the MHC residue features. The output of this submodule is a sequence of updated feature vectors $\{s_j\}$ that is passed on to the next block. This Self IPA submodule is called in two iterative blocks so that the output of the previous block is the input to the next. A skip connection was added in each block (**Algorithm 1, line 15**) to overcome potential degradation problems. The learned Self IPA weights are shared across blocks. The output of the final block is one of the input variables in the Cross Attention Structure module (**Algorithm 1, line 31-32**).

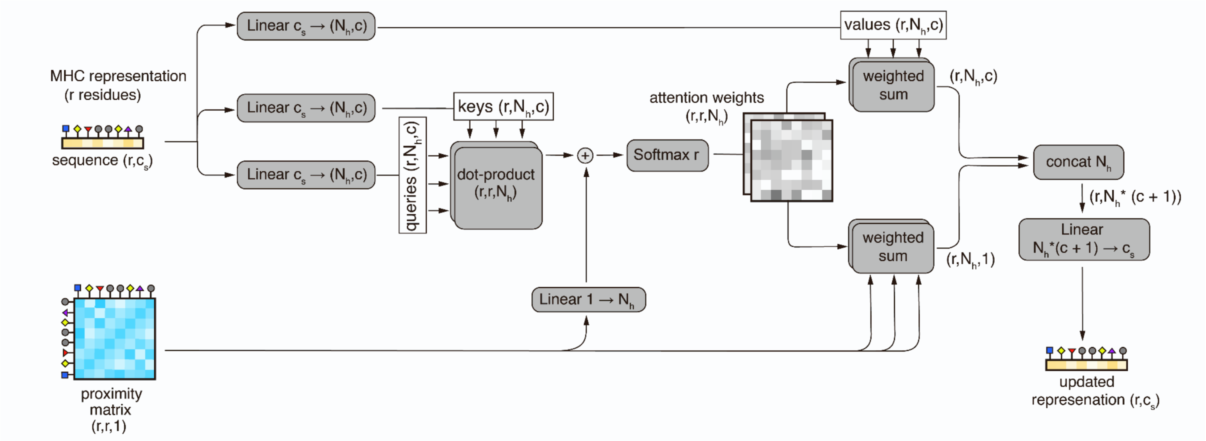

Figure S5: **Flow chart of the MHC Self IPA module, related to Fig. 2.** Dimensions: r: MHC length (r ≤ 200), c_s: amino acid input channels (c_s = 32), c: hidden channels (c = 16), N_h: number of heads (N_h = 2). We use ⊕ for element-wise addition.

Algorithm 2 Self Invariant Point Attention

procedure SelfIPA ($\{s_j\}, \{z_{j\hat{j}}\}, c = 16, N_h = 2$) :

`# Here,` j `and` $\hat{j}$ `correspond to residues in the MHC.`
`#` $q_j^h, k_j^h, v_j^h \in \mathbb{R}^c, h \in \{1, ..., N_h\}$
$q_j^h, k_j^h, v_j^h = \mathrm{LinearNoBias}(s_j)$
$b_{j\hat{j}}^h = \mathrm{LinearNoBias}(z_{j\hat{j}})$

`# Calculate attention weights` $\{a_{j\hat{j}}^h\}$.
$w_L = \sqrt{\frac{1}{2}}$
$a_{j\hat{j}}^h = \mathrm{softmax}_{\hat{j}}(w_L(\frac{1}{\sqrt{c}} q_j^{h\top} k_{\hat{j}}^h + b_{j\hat{j}}^h))$

`# Apply weighted attention.`
$o_j^h = \sum_{\hat{j}} a_{j\hat{j}}^h v_{\hat{j}}^h$
$\tilde{o}_j^h = \sum_{\hat{j}} a_{j\hat{j}}^h z_{j\hat{j}}$

$s_j \leftarrow \mathrm{Linear}(\mathrm{concat}_h(o_j^h, \tilde{o}_j^h))$
return s_j
end procedure

2.3 Module 2: Peptide Self Attention

Within the Main module, the Peptide Self Attention module operates on peptide sequential data (**Algorithm 3** and **Figure S6**). The peptide structure is unknown at this point and this module updates the feature representation of each peptide residue based on its own amino acid type and its sequential neighbors. We intend to design an algorithm that can eventually model peptides with variable lengths other than just 9. For this reason we employed relative position encoding (**Algorithm 3, lines 2-7,**

Fig. S6B). Using the outer differences between residue positions $f_i^{residue\_index}$, we construct a relative positions matrix, where each element $d_{i\hat{i}}$ represents the distance between two residues i and $\hat{i}$, measured as negative for N-terminal distances and positive for C-terminal distances (**Algorithm 3, line 6**). These differences are calculated and one-hot encoded into $c_z = 33$ different bins ($v_{bins} = [-16, -15, ...0...15, 16]$), forming the relative position encodings $\{z_{i\hat{i}}\}$ in **Algorithm 3.** Those relative position encodings are used in the peptide Self Attention computation.

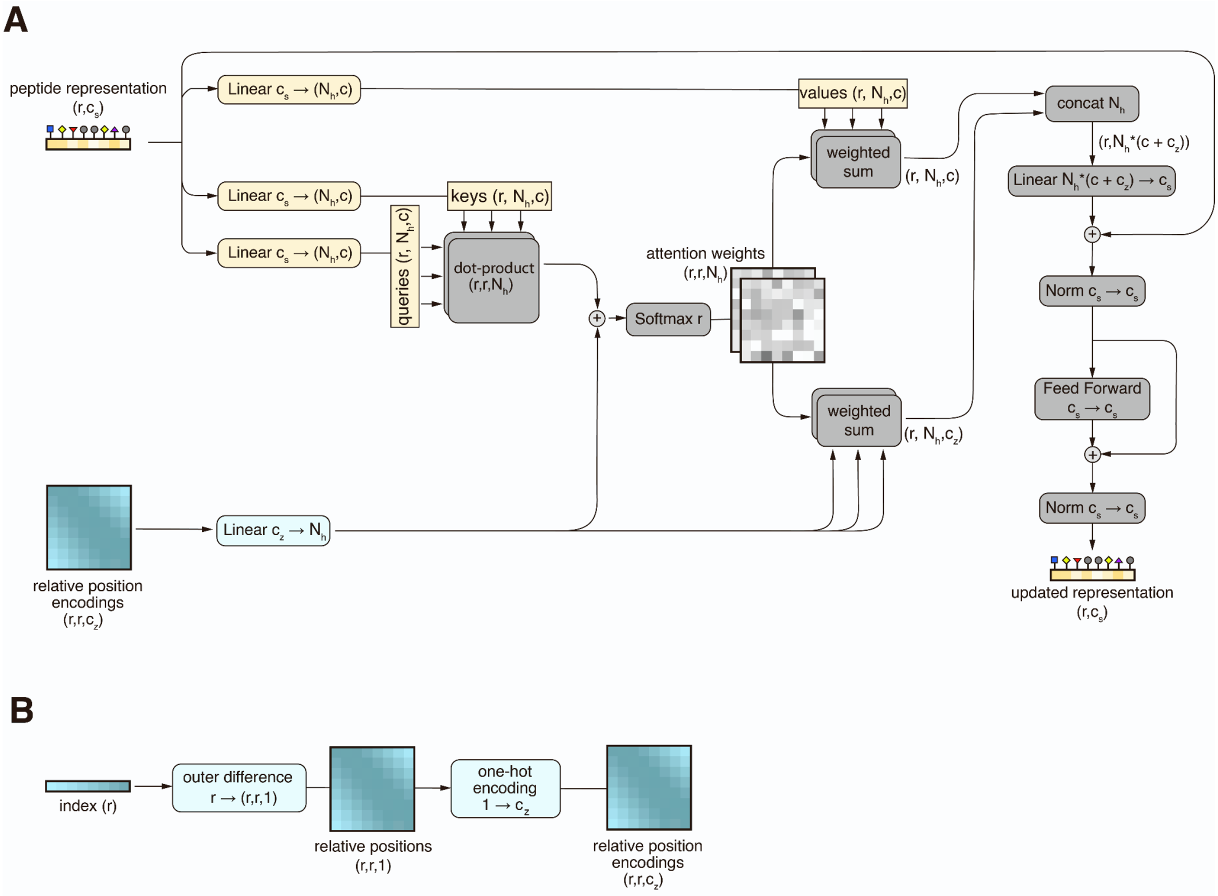

Figure S6: **Flow chart of the Peptide Self Attention module, related to Fig. 2.** Dimensions: r: peptide length (r ≤ 16), c_s: amino acid input channels (c_s = 32), c_z: relative position bins (c_z=33), c: hidden channels (c=16), N_h: heads (N_h=2). We use ⊕ for element-wise addition. **A.** Full module. **B.** Relative position encoding.

The peptide Self Attention is computed similarly to regular multi-headed attention[9]. From the the one-hot encoded input sequence $\{s_i\}$, the queries(q_i^h), keys(k_i^h) and values(v_i^h). The queries and keys are dot multiplied, normalized and then used as the first term for the multi-headed attention weights. The key difference from regular multi-headed attention is that the formula for attention weights includes a second term, additionally to the normalized dot product (**Algorithm 3, line 17**).

This second attention term $\{b^h_{i\hat{\imath}}\}$ is derived from the relative position encodings $\{z_{i\hat{\imath}}\}$. A trainable linear weight matrix converts them into $\{b^h_{i\hat{\imath}}\}$. Softmax is used to convert the sum of the two terms into actual attention weights $\{a^h_{i\hat{\imath}}\}$, summing up to 1.0 over each peptide residue. (**Algorithm 3, line 17**).

The weights in $\{a^h_{i\hat{\imath}}\}$ are multiplied by the values $\{v^h_i\}$ and by one-hot encoded relative position encodings $\{z_{i\hat{\imath}}\}$. The results of those multiplications are concatenated, embedded and then inserted into a feed forward module (**Algorithm 3, lines 24,25**). This feed forward module was placed here from inspiration of the PyTorch transformer encoder[10].

The Peptide Self Attention module is called in two iterative blocks (**Algorithm 1, lines 23-27**). The result of one block altogether is an updated version of the sequence embedding: $\{s_i\}$ (**Algorithm 3, line 31**). The output of the previous block is the input to the next block. A skip connection was added (**Algorithm 1, lines 25**) in each block to overcome potential degradation problems. The output of the final block is used as input to the Cross Attention Structure module (**Algorithm 1, line 31-32**). The learned Peptide Self Attention module's weights are not shared across blocks. Instead, each block maintains its own distinct set of weights.

Algorithm 3 Peptide Self Attention

1: **procedure** PeptideSelfAttention($\{s_i\}, c = 16, c_z = 33, N_h = 2$):
2: # Relative Position Encoding:
3: # i and $\hat{\imath}$ correspond to peptide residues.
4: $f_i^{residue\_index} = i$
5: $v_{bins} = [-16, -15, ..., 16]$
6: $d_{i\hat{\imath}} = f_i^{residue\_index} - f_{\hat{\imath}}^{residue\_index}$
7: $z_{i\hat{\imath}} = \text{one\_hot}(\text{argmin}(|d_{i\hat{\imath}} - v_{bins}|))$
8:
9: # $z_{i\hat{\imath}} \in \mathbb{R}^{c_z}, b^h_{i\hat{\imath}} \in \mathbb{R}, h \in \{1, ..., N_h\}$
10: $b^h_{i\hat{\imath}} = \text{LinearNoBias}(z_{i\hat{\imath}})$
11:
12: # $q_i^h, k_i^h, v_i^h \in \mathbb{R}^{c_h}$
13: $q_i^h, k_i^h, v_i^h = \text{LinearNoBias}(s_i)$
14:
15: # Calculate attention weights $\{a^h_{i\hat{\imath}}\}$.
16: $w_L = \sqrt{\frac{1}{2}}$
17: $a^h_{ij} = \text{softmax}_{\hat{\imath}}(w_L(\frac{1}{\sqrt{c}} q_i^h k_{\hat{\imath}}^h + b^h_{i\hat{\imath}}))$

\# Apply weighted attention.

$\tilde{o}_i^h = \sum_{\hat{i}} a_{i\hat{i}}^h z_{i\hat{i}}$

$o_i^h = \sum_{\hat{i}} a_{i\hat{i}}^h v_{\hat{i}}^h$

\# Skip connection before feed-forward module.

$s_i \mathrel{+}= \mathrm{Linear}(\mathrm{concat}_h(\tilde{o}_i^h, o_i^h))$

$s_i \leftarrow \mathrm{LayerNorm}(\mathrm{Dropout}_{0.1}(s_i))$

\# Feed-forward:

$s_i \mathrel{+}= \mathrm{Linear}(\mathrm{relu}(\mathrm{Linear}(s_i)))$

$s_i \leftarrow \mathrm{LayerNorm}(\mathrm{Dropout}_{0.1}(s_i))$

return $\{s_i\}$

end procedure

2.4 Module 3: Cross Attention Structure module

The Cross Attention Structure module (**Algorithm 4**) is called from inside the Main module, after executing the two Self Attention modules. The Cross Attention Structure module was named after the Structure module in AlphaFold2[7], which inspired its design. It is called *Cross Attention* because it applies a form of multi-headed attention between the MHC and the peptide. The purpose of this module is to predict a structure for the peptide, given peptide sequence features $\{s_i\}$ and an MHC protein, represented by its sequence features $\{s_j\}$ and backbone structure frames $\{T_j\}$ (**Subsection 1.2.1**). This module also returns an updated peptide representation $\{s_i\}$ that contains sequence and structural information of the predicted pMHC structure. This updated representation is subsequently passed to the BA module for BA predictions. Two iterative blocks are used (**Algorithm 4, lines 15-53**) to consider a longer range of residue attentions. Each block contains multiple skip connections to overcome potential gradient degradation problems.

In each block, the module updates both the peptide's feature representation $\{s_i\}$ and its backbone frames $\{T_i\}$. Cross Invariant Point Attention (*Cross IPA*) is applied first (**Subsection 2.4.1**), using MHC information to update $\{s_i\}$. Cross IPA is followed by a transitional module, a dropout operation and a normalization layer (**Algorithm 4, lines 28,29**), the purpose of which are to make s_i-encoded information more expressive. Next, the $\{s_i\}$ variable is used as input for updating $\{T_i\}$ (**Subsection 2.4.2**). From $\{T_i\}$, the positions of the Cα atom and positions of its directly connected atoms N, C, Cβ will be calculated to be saved in the atomic position array $\{\vec{x}_i^{all}\}$. The

next step is to predict the side chain torsion angles χ_1, χ_2, χ_3, χ_4 from the updated $\{s_i\}$ as input. The side chain frames $\{T_i^{sidechain}\}$ are calculated from $\{T_i\}$ and the predicted side chain torsion angles. From $\{T_i^{sidechain}\}$ and the corresponding rigid groups (**AlphaFold2**[7] **Suppl. Table 2**), all other atom positions are calculated. Finally, the ω torsion angles are calculated from the resulting $C\alpha_i$-C_i-N_{i+1}-$C\alpha_{i+1}$ positions (**Algorithm 7**). The loss function will compare those ω angles with the ground truth later (**Fig. S4CD**). The resulting loss values will be used for feedback in training the network. This additional computation step was added to boost accuracy of the predicted ω angle output.

Once a Cross Attention Structure module block is completed, the resulting $\{s_i\}$ and $\{T_i\}$ are used as input for the next block. The output of the Cross Attention Structure module are the updated peptide features $\{s_i\}$, the local frames $\{T_i^{all}\}$, atomic positions $\{\vec{x}_i^{all}\}$ and torsion angles $\{\vec{\alpha}_i^{all}\}$ from the final block. The output peptide features $\{s_i\}$ will be used as input for the SwiftMHC BA Predictor (**Subsection 2.5**).

Algorithm 4 Cross Attention Structure module

```
procedure CrossAttentionStructureModule ({s_i^initial}, {s_j^initial},
                                         {T_j}, N_blocks = 2):
    # i corresponds to a peptide residue.
    # j corresponds to a MHC residue.
    s_i^initial ← LayerNorm(s_i^initial)
    s_j^initial ← LayerNorm(s_j^initial)

    # T_i is initialized as a series of identity frames, this works well
    # only when the preprocessing superimposed
    # all input structures in the same orientation.
    q_I = (1,0,0,0) # quaternion definition:  q = (w,x,y,z)
    t_I = (0,0,0)
    T_i = (q_I, t_I)

```

for all $l \in [1, ..., N_{blocks}]$ **do**

`# Cross Invariant Point Attention (CrossIPA)`
`# between peptide and MHC:`

`# The weights of the cross IPA are shared across blocks.`
`# For CrossIPA see Algorithm 5.`
$\{s_i\} += \text{CrossIPA}(\{s_i\}, \{s_j\}, \{T_i\}, \{T_j\})$
$s_i \leftarrow \text{LayerNorm}(\text{Dropout}_{0.1}(s_i))$

`# Structure module transition:`

`# The weights of the Linears and LayerNorm`
`# are shared across blocks.`
$s_i \leftarrow s_i + \text{Linear}(\text{relu}(\text{Linear}(\text{relu}(\text{Linear}(s_i)))))$
$s_i \leftarrow \text{LayerNorm}(\text{Dropout}_{0.1}(s_i))$

`# BackboneUpdate is similar to AlphaFold2`[1] `Suppl. Algorithm 23.`
`# It updates the backbone frames by transformation as`
`# described in AlphaFold2`[1] `Suppl. Subsection 1.1`

`# All weights of BackboneUpdate are shared across blocks.`
`# For BackboneUpdate, see Algorithm 6.`
$T_i \leftarrow T_i \circ \text{BackboneUpdate}(s_i)$

`# Predict side chain and backbone angles` $\phi, \psi, \omega, \chi_1, \chi_2, \chi_3, \chi_4$
`# as sin,cos vectors.`
`# All weights of side chain angle prediction`
`# are shared across blocks.`
$a_i = \text{Linear}(\text{relu}(s_i)) + \text{Linear}(\text{relu}(s_i^{initial}))$
$a_i \leftarrow a_i + \text{Linear}(\text{relu}(\text{Linear}(\text{relu}(a_i))))$
$a_i \leftarrow a_i + \text{Linear}(\text{relu}(\text{Linear}(\text{relu}(a_i))))$
$\vec{\alpha}_i^{all} = \text{normalize\_vec}(\text{Linear}(\text{relu}(a_i)))$

47: # Rotation gradients are disabled between blocks,
48: # to stabilize training.
49: **if** $l < N_{block}$ **then**
50: $q_i, \vec{t}_i \leftarrow T_i$
51: $T_i \leftarrow (\text{stopgrad}(q_i), \vec{t}_i)$
52: **end if**
53: **end for**
54:
55: # The backbone translation is scaled by a factor 10.
56: $q_i, \vec{t}_i \leftarrow T_i$
57: $\vec{t}_i \leftarrow 10\vec{t}_i$
58: $T_i \leftarrow (q_i, \vec{t}_i)$
59:
60: # ComputeAllAtomCoordinates is from AlphaFold2[1] Suppl. Algorithm 24.
61: $T_i^{sidechain}, \vec{x}_i^{all} = \text{ComputeAllAtomCoordinates}(T_i, \vec{\alpha}_i^{all})$
62: $T_i^{all} = \text{concat}(T_i, T_i^{sidechain})$
63:
64: # Update the peptide ω angles from the computed backbone
65: # coordinates.
66: # For CalculateTorsion, see Algorithm 7.
67: $(\vec{\omega}_i, \vec{\phi}_i, \vec{\psi}_i, \vec{\chi_1}_i, ...) = \vec{\alpha}_i^{all}$
68: $\{\vec{x}_i^N, \vec{x}_i^{C\alpha}, \vec{x}_i^C, ...\} = \vec{x}_i^{all}$
69: $\{\vec{x}_{i+1}^N, \vec{x}_{i+1}^{C\alpha}, \vec{x}_{i+1}^C, ...\} = \vec{x}_{i+1}^{all}$
70: $\vec{\omega}_i = \text{CalculateTorsion}(\vec{x}_i^{C\alpha}, \vec{x}_i^C, \vec{x}_{i+1}^N, \vec{x}_{i+1}^{C\alpha})$
71: $\vec{\alpha}_i^{all} = (\vec{\omega}_i, \vec{\phi}_i, \vec{\psi}_i, \vec{\chi_1}_i, ...)$
72:
73: **return** $\{\vec{x}_i^{all}\}, \{T_i^{all}\}, \{\vec{\alpha}_i^{all}\}, \{s_i\}$
74: **end procedure**

2.4.1 Cross Invariant Point Attention

The Cross Invariant Point Attention (Cross IPA) module (**Algorithm 5** and **Fig. S7**) is part of the Cross Attention Structure module (**Algorithm 4, line 21**). SwiftMHC uses the Cross IPA module to update the peptide representation $\{s_i\}$ using the predicted $\{T_i\}$ and information from the MHC groove, namely $\{s_j\}$ and the invariable $\{T_j\}$. Peptide residue representations serve as queries, and MHC representations as keys and values in the attention mechanism. The updated peptide representation $\{s_i\}$ will be used to update the coordinates of peptide residues $\{T_i\}$ and to be passed to the BA module to predict BA.

Cross IPA is a multi-headed Cross Attention module that takes into account both the sequence features and geometry. Thus the attention weights have two components:

sequence-level $q_i^{h\top} k_j^h$ and geometric level $||T_i \circ \vec{q}_i^{hp} - T_j \circ \vec{k}_j^{hp}||$ (**Algorithm 5**, **line 22**). The first term $q_i^{h\top} k_j^h$ calculates the attention weights between each peptide residue and each MHC residue in terms of amino acid similarities. To calculate the geometric attention, 4 virtual points are created for each peptide residue and 4 virtual points are created for each MHC residue (**Algorithm 5, lines 11-13**). The distance $||T_i \circ \vec{q}_i^{hp} - T_j \circ \vec{k}_j^{hp}||$ is calculated between a virtual point of a peptide residue and a virtual point of a MHC residue. The virtual points are initially placed in the backbone local frame space (see **Subsection 1.2.1** for explanation) and the peptide $\{T_i\}$ and MHC $\{T_j\}$ are used to transform those virtual points to global space. The global space coordinates of those virtual points are used to calculate the squared distances that are summed up to form the second term in the attention weights calculation (**Algorithm 5, line 22**).

The Cross Invariant Point Attention module does not operate on the entire MHC G-domain. For speed purposes, only a masked selection of MHC residues is used. We selected all residues that are within a 10 Å radius from the peptide in any of the known pMHC-I structures.

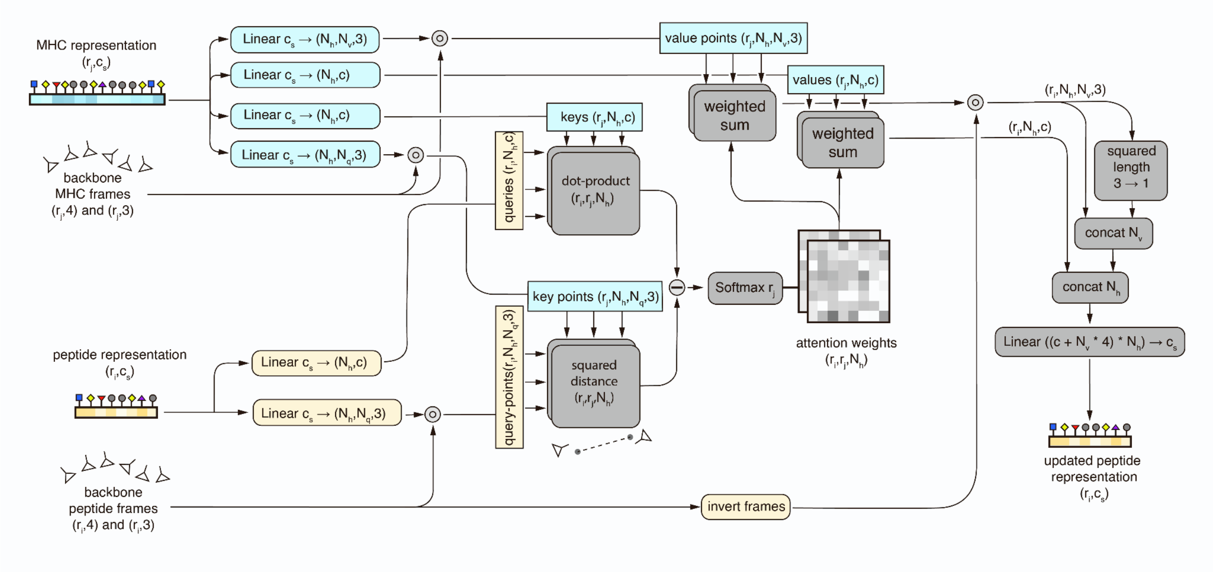

Figure S7: Flow chart of the Cross IPA module, related to Fig. 2. Dimensions: r_i: peptide length ($r_i \leq 16$), r_j: MHC length ($r_j \leq 200$), c_s: amino acid input channels ($c_s = 32$), N_q : number of generated query and key points($N_q = 4$), N_v: number of generated value points ($N_v = 8$) , c: hidden channels (c = 16), N_h: heads ($N_h = 2$). We use ◎ for transforming vectors by frames and ⊖ for element-wise subtraction. The two triangles represent two local frames: one frame represents a peptide residue and transforms query points; the other frame represents a MHC residue and transforms key points. A dashed line indicates distance measured between those two invariant points predicted within the frame-local space.

Algorithm 5 Cross Invariant Point Attention

procedure CrossIPA ($\{s_i\}, \{s_j\}, \{T_i\}, \{T_j\}, \{T_i\},$
$c = 16, N_h = 2, N_q = 4, N_v = 8$):
  # i corresponds to a peptide residue.
  # j corresponds to a MHC residue.

  # $q_i^h, k_j^h, v_j^h \in \mathbb{R}^c, h \in \{1, ..., N_h\}$
  $q_i^h = \text{LinearNoBias}(s_i)$
  $k_j^h = \text{LinearNoBias}(s_j)$
  $v_j^h = \text{LinearNoBias}(s_j)$

  # $\vec{q}_i^{hp}, \vec{k}_j^{hp} \in \mathbb{R}^3, p \in \{1, ..., N_q\}$
  $\vec{q}_i^{hp} = \text{LinearNoBias}(s_i)$
  $\vec{k}_j^{hp} = \text{LinearNoBias}(s_j)$

  # $\vec{v}_j^{hp} \in \mathbb{R}^3, p \in \{1, ..., N_v\}$
  $\vec{v}_j^{hp} = \text{LinearNoBias}(s_j)$

  # Calculate attention weights $\{a_{ij}^h\}$.
  # The head weights $\{\gamma^h\}$ are trainable.
  $w_C = \sqrt{\frac{2}{9N_q}}$
  $w_L = \sqrt{\frac{1}{2}}$
  $a_{ij}^h = \text{softmax}_j(w_L(\frac{1}{\sqrt{c}} q_i^{h\top} k_j^h - \frac{\gamma^h w_C}{2} \sum_p ||T_i \circ \vec{q}_i^{hp} - T_j \circ \vec{k}_j^{hp}||^2))$

  # Apply weighted attention.
  $o_i^h = \sum_j a_{ij}^h v_j^h$
  $\vec{o}_i^{hp} = T_i^{-1} \circ \sum_j a_{ij}^h (T_j \circ \vec{v}_j^{hp})$

  $s_i = \text{Linear}(\text{concat}_{h,p}(o_i^h, \vec{o}_i^{hp}, ||\vec{o}_i^{hp}||))$
  return s_i
end procedure

2.4.2 Backbone Updating

The SwiftMHC Cross Structure module updates the backbone structure in two iterative blocks (**Algorithm 4, line 37**), taking the peptide representation s_i as input. The backbone structure is initialized as identity frames, meaning the backbones of all peptide residues are overlapped at the origin and have the same orientations. Each block calls the *Backbone Update* module (**Algorithm 6**), that predicts an orientation

quaternion q_i and a translation vector $\vec{t}_i$. The two make up a frame T_i that is to be transformed by the previous backbone frame, resulting in the next backbone frame: $T_i \leftarrow T_i \circ \mathrm{BackboneUpdate}(s_i)$. This procedure is almost equal to **AlphaFold2**[7] **Suppl. Algorithm 23**, except that a quaternion is used for rotation, rather than a rotation matrix. The choice to use quaternions was made in order to reduce the number of floating point multiplications and additions to speed up the procedure.

Algorithm 6 BackboneUpdate

procedure BackboneUpdate(s_i):
  # $b_i, c_i, d_i \in \mathbb{R}, \vec{t}_i \in \mathbb{R}^3$
  $b_i, c_i, d_i, \vec{t}_i = \mathrm{Linear}(s_i)$

  # Normalize the quaternion.
  $q_i = (1, b_i, c_i, d_i)/\sqrt{1 + b_i^2 + c_i^2 + d_i^2}$

  $T_i = (q_i, \vec{t}_i)$
  return T_i
end procedure

2.4.3 Torsion Angles and Rigid Groups

SwiftMHC uses 8 frames to describe the conformation of every amino acid. The first frame is reserved for the orientation and the position of the backbone atoms: Cα and N, O and Cβ. The 7 remaining frames have been assigned to torsion angles and the corresponding rigid groups (**Fig. 2C**). The selection of atoms for those rigid groups is the same as displayed in **AlphaFold2**[7] **Suppl. Table 2**.

The ω torsion angle is a special case. In proteins, the bond between the carbonyl carbon (C) and the nitrogen (N) of the next amino acid is typically planar due to resonance, and the ω angle around this bond is often restricted to 0° (cis) or 180° (trans), with the trans configuration being much more common. After the backbone updating is complete and the positioning and orientation of atoms Cα, C and N in the backbone rigid groups is known, the ω angle is calculated using **Algorithm 7**. This ω angle is combined with the other predicted torsion angles (ϕ, ψ, χ_1, χ_2, χ_3, χ_4) to serve as input to the torsion loss function. This allows the network model to learn to avoid incorrect ω conformations.

Algorithm 7 CalculateTorsion

procedure CalculateTorsion ($\vec{x}_1, \vec{x}_2, \vec{x}_3, \vec{x}_4$):

`# Calculate 3 interatomic bond vectors from 4 atomic positions.`
$\vec{b} = \vec{x}_3 - \vec{x}_2$
$\vec{n} = \frac{\vec{b}}{||\vec{b}||}$

$\vec{v}_1 = \vec{x}_2 - \vec{x}_1$
$\vec{v}_2 = \vec{x}_3 - \vec{x}_4$

`# Convert the bond vectors to Newman projections`
`# along a plane perpendicular to the central (2,3) bond.`
$\vec{p}_1 = \vec{v}_1 - (\vec{n}^\top \vec{v}_1)\vec{n}$
$\vec{e}_1 = \frac{\vec{p}_1}{||\vec{p}_1||}$

$\vec{p}_2 = \vec{v}_2 - (\vec{n}^\top \vec{v}_2)\vec{n}$
$\vec{e}_2 = \frac{\vec{p}_2}{||\vec{p}_2||}$

`# Calculate the torsion angle sin,cos from the Newman projections.`
$\alpha^{cos} = \vec{e}_1^\top \vec{e}_2$

if $(\vec{e}_1 \times \vec{e}_2)^\top \vec{n} < 0$ **then**
  $\alpha^{sin} = -||\vec{e}_1 \times \vec{e}_2||$
else
  $\alpha^{sin} = ||\vec{e}_1 \times \vec{e}_2||$
end if

$\alpha = (\alpha^{sin}, \alpha^{cos})$
return α
end procedure

Newman projections represent the conformation of a molecule, by projecting the positions of the atoms around a specific bond in a plane, perpendicular to that bond (see **Fig. S4D** for an example). Such a projection provides insights into the torsion angle. The ω calculation function in **Algorithm 7** creates a Newman projection of the Cα atoms around each peptide bond: C-N. This is the type of bond that connects every pair of adjacent residues (**Fig. S4CD**). From the resulting Newman projections, the *sin(ω)* and *cos(ω)* are calculated to be used in the loss function (**Subsection 2.6**).

We chose to calculate the cosines and sines of the torsion angles instead of the actual angles for two key reasons. First, this approach simplifies the torsion angle loss function, allowing the use of a straightforward normalization and squared error

function when working with sin, cos vectors. Second, it improves computational efficiency, as cosines and sines can be directly computed using dot and cross products, eliminating the need for additional computationally expensive arcsin and arccos operations.

2.5 Module 4: Residue-wise Binding Affinity Predictor

To enable SwiftMHC to handle peptides of varying lengths, we implemented a BA predictor that processes input sequences residue by residue (**Algorithm 8**). This BA predictor is called within the main module (**Algorithm 1, line 40**). Numerical BA is predicted by a multi-layer perceptron (MLP), that individually processes each residue of the updated sequence $\{s_i\}$. The BA prediction is the sum of the MLP outputs over all peptide residues (**Algorithm 8, line 8**). This BA value is trained to approach *1 - $log_{50000}(K_d)$* or *1 - $log_{50000}(IC_{50})$*, depending on which of the two data is available from the IEDB, where K_d and IC_{50} are experimentally determined parameters that reflect binding interactions and functional inhibition, respectively.

Algorithm 8 Binding Affininty Predictor

1: **procedure** BindingAffinintyPredictor($\{s_i\}$):
2:     # i corresponds to a peptide residue.
3:     # r is the peptide length
4:     # $i \in \{0, 1, 2, .., r\}$
5:     $p_i = \text{Linear}(\text{relu}(\text{Linear}(s_i)))$
6:
7:     # BA corresponds to $1 - \log_{50000}(\text{IC}_{50})$ or $1 - \log_{50000}(\text{K}_\text{d})$
8:     $\text{BA} = \sum_i^r p_i$
9:     **return** BA
10: **end procedure**

2.6. Loss Terms

We train the modules in SwiftMHC from the following loss terms: *BA loss, backbone and side chain Frame Aligned Point Error (*FAPE*), Torsion angle loss* and *Structural Violations*. This subsection will explain each of these loss terms in detail.

BA loss ($\mathcal{L}_{BA}$ in **Algorithm 9**) is calculated on numerical output BA values that are compared in a MSE function against either the *1 - $log_{50000}(IC_{50})$* or *1 - $log_{50000}(K_d)$*. The BA loss value expresses how much the BA output deviates from the experimental value.

We also calculated FAPE loss as in AlphaFold2. FAPE measures the predictive error of the position of each atom i relative to the local frame of residue j (see **Suppl. Algorithm 28, computeFAPE** and **Fig. 3f** in the AlphaFold2 paper for details). For example, if we hope to measure how well the CA of a peptide residue i is predicted

relative to a MHC residue j, we can calculate FAPE using: $d_{ij} = \sqrt{\epsilon + || T_j^{-1} \circ \vec{x}_i - T_j^{-1} \circ \vec{x}_i^{true} ||^2}$, where $\vec{x}_i$ is the predicted position for the CA atom of MHC residue i, $\vec{x}_i^{true}$ is the true position of the CA atom of peptide residue i, T_j is the local frame of MHC residue j, T_j^{-1} is the global to local frame transformation of residue j and ϵ = 10^{-4} $Å^2$. The distance d_{ij} is also clamped here using the same 10.0 Å distance. Using the local frames, FAPE also allows us to measure the chirality errors. Our FAPE loss contains a Backbone FAPE term and a side-chain FAPE term.

Backbone FAPE ($\mathcal{L}_{FAPE}^{backbone}$ in **Algorithm 9**) is calculated on predicted vs. true peptide Cα positions relative to the MHC structure. The MHC input frames T_j and the true peptide positions $\vec{x}_i^{true}$ are derived from the target pMHC structure, while the input positions $\vec{x}_i$ are predicted from the peptide. This approach allows FAPE to measure the peptide's positions relative to the MHC it binds to. The resulting FAPE value expresses how different the output peptide frame positions are from the true peptide frames, as seen from inside each of the MHC frame-local coordinate systems.

Side chain FAPE ($\mathcal{L}_{FAPE}^{sidechain}$ in **Algorithm 9**) is calculated on the peptide side chain atoms, as described in **AlphaFold2**[7] **Suppl. Algorithm 28**. For this side chain loss term, both the input frames T_i and input positions $\vec{x}_i$ are solely derived from the peptide, with no involvement from the MHC. This FAPE value expresses how different the atoms from the predicted peptide side chain are from their true positions, as seen from inside each of the peptide backbone-local coordinate systems. The true structural data originates from X-ray crystallography, where N, C, and O atoms are often indistinguishable. As a result, some amino acids exhibit 180° symmetry in their side chains, leading to the possibility of two valid frames. We use an implementation of the *renameSymmetricGroundTruthAtoms* algorithm (**AlphaFold2**[7] **Suppl. Algorithm 26**) to address this. Similar to AlphaFold2, a clamped L1-loss is applied to both backbone and sidechain FAPE (**AlphaFold2**[7] **Suppl. Algorithm 28, Line 4**).

Torsion angle loss ($\mathcal{L}_{torsion}^{all}$ in **Algorithm 9**) is calculated as described in **AlphaFold2**[7] **Suppl. Algorithm 27**. Only torsion angles (ϕ, ψ, ω, χ_1, χ_2, χ_3, χ_4) from the peptide are used in the equation, while MHC is ignored. The errors express how much these angles deviate from the angles in the true structure. For the side chain torsion angles in some amino acids there is a 180° symmetry, meaning that there can be two true angles. The torsion angle loss algorithm is designed to take both possibilities into consideration and select the best match.

Algorithm 9 Calculate Loss

procedure CalculateLoss (BA, BA^{true},
$\{\vec{x}_i^{all}\}, \{\vec{x}_j^{all}\}, \{\vec{x}_i^{true,all}\}, \{\vec{x}_i^{alt\ truth,all}\},$
$\{T_i^{all}\}, \{T_j\}, \{T_i^{true,all}\}, \{T_i^{alt\ truth,all}\},$
$\{\vec{\alpha}_i^{all}\}, \{\vec{\alpha}_i^{true,all}\}, \{\vec{\alpha}_i^{alt\ truth,all}\})$:

`# `i` corresponds to a peptide residue.`

`# `j` corresponds to a MHC residue.`

`# For ComputeFAPE, see Alphafold2`[1]` Suppl. Algorithm 28`

$\{\vec{x}_i^{N}, \vec{x}_i^{C\alpha}, \vec{x}_i^{C}, ..\} = \vec{x}_i^{all}$

$\{\vec{x}_i^{true,N}, \vec{x}_i^{true,C\alpha}, \vec{x}_i^{true,C}, ..\} = \vec{x}_i^{true,all}$

$\mathcal{L}_{\text{FAPE}}^{backbone} = \text{ComputeFAPE}(\{T_j\}, \{\vec{x}_i^{C\alpha}\}, \{T_j\}, \{\vec{x}_i^{true,C\alpha}\})$

$\mathcal{L}_{FAPE}^{sidechain} = \text{ComputeFAPE}(\{T_i^{sidechain}\}, \{\vec{x}_i^{all}\}, \{T_i^{true,sidechain}\}, \{\vec{x}_i^{true,all}\})$

```
# For renameSymmetricGroundTruthAtoms, see
# Alphafold2[1] Suppl. Algorithm 26
```
$\{T_i^{true,sidechain}\}, \{\vec{x}_i^{true,all}\} \leftarrow \text{renameSymmetricGroundTruthAtoms}(\{T_i^{sidechain}\}, \{\vec{x}_i^{all}\}, \{T_i^{true,sidechain}\}, \{T_i^{alt\ truth,sidechain}\}, \{\vec{x}_i^{true,all}\}, \{\vec{x}_i^{alt\ truth,all}\})$

```
# For TorsionAngleLoss, see Alphafold2[1] Suppl. Algorithm 27
```
$\mathcal{L}_{torsion}^{all} = \text{TorsionAngleLoss}(\{\vec{\alpha}_i^{all}\}, \{\vec{\alpha}_i^{true,all}\}, \{\vec{\alpha}_i^{alt\ truth,all}\})$

```
# For StructuralViolations, see Algorithm 10
```
$\mathcal{L}_{\text{viol}} = \text{StructuralViolations}(\{x_i^{all}\}, \{x_j^{all}\})$

```
# BA^true ∈ ℝ
```
$\mathcal{L}_{\text{BA}} = \text{MeanSquareError}(\text{BA}, \text{BA}^{\text{true}})$

```
# Add on losses, according to tuning settings.
```
$\mathcal{L}_{\text{tot}} = 0$
if baTune **then**
  $\mathcal{L}_{tot} += \mathcal{L}_{\text{BA}}$
end if
if fapeTune **then**
  $\mathcal{L}_{\text{tot}} += \mathcal{L}_{\text{FAPE}}^{backbone} + \mathcal{L}_{\text{FAPE}}^{sidechain}$
end if
if torsionTune **then**
  $\mathcal{L}_{\text{tot}} += \mathcal{L}_{\text{torsion}}^{all}$
end if
if fineTune **then**
  $\mathcal{L}_{\text{tot}} += \mathcal{L}_{\text{viol}}$
end if
return $\mathcal{L}_{\text{tot}}$
end procedure

Structural violations ($\mathcal{L}_{viol}$ in **Algorithm 9 & 10**) are calculated by comparing the predicted bond angles, bond lengths and non-bonded distances to literature values as described in **AlphaFold2**[7] **Suppl. Subsection 1.9.11**. For calculating bond angle and bond distance violations, only the peptide atoms are used. For clash violations, atoms from both the peptide and the MHC are used. The structural violation loss terms express how much the bond angles, bond lengths and non-bonded distances deviate from their literature values. Such loss terms are only included during the final *fine tuning* phase of training (**Subsection 3.1**).

Algorithm 10 Structural Violations

procedure StructuralViolations ($\{x_i^{all}\}, \{x_j^{all}\}$):

Structural violations, computed in a similar way to
AlphaFold2[1] Suppl. Subsection 1.9.11

i corresponds to a peptide residue.
j corresponds to a MHC residue.

Bond length loss is calculated over backbone atoms only.
$l_{lit}^{general} = 1.329\text{Å}, l_{lit}^{proline} = 1.341\text{Å}$
$\tau^{bondlength} = 12\sigma, \sigma^{general} = 0.014\text{Å}, \sigma^{proline} = 0.016\text{Å}$

$$\mathcal{L}_{viol}^{bondlength} = \frac{1}{N_{bonds}} \sum_{i=1}^{N_{bonds}} max(\mid l_{pred}^{i} - l_{lit}^{i} \mid - \tau^{bondlength}, 0)$$

Angle loss is calculated over backbone atoms only.
$\alpha_{lit}^{CNC\alpha} = 121.352°, \alpha_{lit}^{C\alpha CN} = 116.568°$,
$\tau^{bondangle} = 12\sigma, \sigma^{CNC\alpha} = 0.0311°, \sigma^{C\alpha CN} = 0.0353°$

$$\mathcal{L}_{viol}^{bondangle} = \frac{1}{N_{angles}} \sum_{i=1}^{N_{angles}} max(\mid cos\ \alpha_{pred}^{i} - cos\ \alpha_{lit}^{i} \mid - \tau^{bondangle}, 0)$$

Clash loss is calculated between residues only
$\tau^{clash} = 1.5\text{Å}$
$\mathrm{N_{nbpairs}}$ counts the total number of non-bonded atom-atom interactions

$$\mathcal{L}_{\mathrm{viol}}^{clash} = \frac{1}{\mathrm{N_{nbpairs}}} \sum_{i=1}^{\mathrm{N_{nbpairs}}} \max(d_{lit}^{i} - d_{pred}^{i} - \tau^{clash}, 0)$$

$$\mathcal{L}_{\mathrm{viol}} = \mathcal{L}_{\mathrm{viol}}^{bondlength} + \mathcal{L}_{\mathrm{viol}}^{bondangle} + \mathcal{L}_{\mathrm{viol}}^{clash}$$

return $\mathcal{L}_{\mathrm{viol}}$

end procedure

Result S1 - SwiftMHC's robustness to structural variation in MHC molecules, related to Fig. 3

We considered how best to evaluate SwiftMHC's robustness to structural variation in MHC molecules. Although the reviewer suggested assessing homology modeling variance, we reasoned that X-ray structures are already used as templates in such models and therefore directly analyzed X-ray structures instead. Specifically, we performed an all-against-all TM-align comparison of the G-domains (IMGT residues 2–180) from 202 HLA-A*02:01 X-ray structures. The pairwise RMSD values showed a mean of **0.53 Å** and a maximum of **1.19 Å**, indicating minimal structural variability within this allele. Given this limited range, and considering that the training models were themselves constructed using the homology models from X-ray templates, we did not pursue additional robustness experiments. We therefore believe SwiftMHC should be reasonably robust to homology-modeled MHC structures.

3. References

1. Vita, R., Mahajan, S., Overton, J.A., Dhanda, S.K., Martini, S., Cantrell, J.R., Wheeler, D.K., Sette, A., and Peters, B. (2019). The Immune Epitope Database (IEDB): 2018 update. Nucleic Acids Res. *47*, D339–D343. https://doi.org/10.1093/nar/gky1006.

2. Andreatta, M., Alvarez, B., and Nielsen, M. (2017). GibbsCluster: unsupervised clustering and alignment of peptide sequences. Nucleic Acids Res. *45*, W458–W463. https://doi.org/10.1093/nar/gkx248.

3. Burley, S.K., Bhikadiya, C., Bi, C., Bittrich, S., Chen, L., Crichlow, G.V., Duarte, J.M., Dutta, S., Fayazi, M., Feng, Z., et al. (2022). RCSB Protein Data Bank: Celebrating 50 years of the PDB with new tools for understanding and visualizing biological macromolecules in 3D. Protein Sci. *31*, 187–208. https://doi.org/10.1002/pro.4213.

4. Parizi, F.M., Marzella, D.F., Ramakrishnan, G., ‘T Hoen, P.A.C., Karimi-Jafari, M.H., and Xue, L.C. (2023). PANDORA v2.0: Benchmarking peptide-MHC II models and software improvements. Front. Immunol. *14*, 1285899. https://doi.org/10.3389/fimmu.2023.1285899.

5. Barker, D.J., Maccari, G., Georgiou, X., Cooper, M.A., Flicek, P., Robinson, J., and Marsh, S.G.E. (2023). The IPD-IMGT/HLA Database. Nucleic Acids Res. *51*, D1053–D1060. https://doi.org/10.1093/nar/gkac1011.

6. O’Donnell, T.J., Rubinsteyn, A., and Laserson, U. (2020). MHCflurry 2.0: Improved Pan-Allele Prediction of MHC Class I-Presented Peptides by Incorporating Antigen Processing. Cell Syst. *11*, 42-48.e7. https://doi.org/10.1016/j.cels.2020.06.010.

7. Jumper, John (2021). Highly accurate protein structure prediction with AlphaFold. nature.

8. Ahdritz, G., Bouatta, N., Floristean, C., Kadyan, S., Xia, Q., Gerecke, W., O’Donnell, T.J., Berenberg, D., Fisk, I., Zanichelli, N., et al. (2022). OpenFold: Retraining AlphaFold2 yields new insights into its learning mechanisms and capacity for generalization. Preprint, https://doi.org/10.1101/2022.11.20.517210 https://doi.org/10.1101/2022.11.20.517210.

9. Vaswani, A., Shazeer, N., Parmar, N., Uszkoreit, J., Jones, L., Gomez, A.N., Kaiser, L., and Polosukhin, I. (2023). Attention Is All You Need. Preprint at arXiv, https://doi.org/10.48550/arXiv.1706.03762 https://doi.org/10.48550/arXiv.1706.03762.

10. Imambi, S., Prakash, K.B., and Kanagachidambaresan, G.R. (2021). PyTorch. In Programming with TensorFlow EAI/Springer Innovations in Communication and Computing., K. B. Prakash and G. R. Kanagachidambaresan, eds. (Springer International Publishing), pp. 87–104. https://doi.org/10.1007/978-3-030-57077-4_10.
